# Prediction of compressive strength of concrete based on improved artificial bee colony-multilayer perceptron algorithm

**DOI:** 10.1038/s41598-024-57131-w

**Published:** 2024-03-17

**Authors:** Ping Li, Yanru Zhang, Jiming Gu, Shiwei Duan

**Affiliations:** School of Mechanical Engineering, Anhui University of Technology, Ma’anshan, 243032 China

**Keywords:** Concrete, Compressive strength prediction, Improved artificial bee colony algorithm, Multilayer perceptron, Engineering, Civil engineering

## Abstract

There are many factors that affect the compressive strength of concrete. The relationship between compressive strength and these factors is a complex nonlinear problem. Empirical formulas commonly used to predict the compressive strength of concrete are based on summarizing experimental data of several different mix proportions and curing periods, and their generality is poor. This article proposes an improved artificial bee colony algorithm (IABC) and a multilayer perceptron (MLP) coupled model for predicting the compressive strength of concrete. To address the shortcomings of the basic artificial bee colony algorithm, such as easily falling into local optima and slow convergence speed, this article introduces a Gaussian mutation operator into the basic artificial bee colony algorithm to optimize the initial honey source position and designs an MLP neural network model based on the improved artificial bee colony algorithm (IABC-MLP). Compared with traditional strength prediction models, the ABC-MLP model can better capture the nonlinear relationship of the compressive strength of concrete and achieve higher prediction accuracy when considering the compound effect of multiple factors. The IABC-MLP model built in this study is compared with the ABC-MLP and particle swarm optimization (PSO) coupling algorithms. The research shows that IABC can significantly improve the training and prediction accuracy of MLP. Compared with the ABC-MLP and PSO-MLP coupling models, the training accuracy of the IABC-MLP model is increased by 1.6% and 4.5%, respectively. This model is also compared with common individual learning algorithms such as MLP, decision tree (DT), support vector machine regression (SVR), and random forest algorithms (RF). Based on the comparison of prediction results, the proposed method shows excellent performance in all indicators and demonstrates the superiority of heuristic algorithms in predicting the compressive strength of concrete.

## Introduction

Concrete is one of the most commonly used materials in construction engineering, and its compressive strength is an important reference index for structural design and construction. As a multiphase composite material, the complex and diverse composition of concrete, differences in curing time and environment, and nonlinear coupling relationships between its constituent materials pose many challenges to the establishment of compression strength prediction models. Currently, in engineering applications, engineers mostly conduct mechanical performance tests on concrete materials and establish empirical-type compression strength prediction models based on the test data. However, such models are often based on the empirical summaries of test data for one or two types of concrete. When changing one of the constituent materials or curing time, the accuracy of this prediction model will greatly reduce. Therefore, these concrete strength prediction models based on specific constituent materials and curing conditions are often one-sided.

With the development of artificial intelligence, machine learning has drawn the attention of civil engineers. Machine learning methods, due to their ability to identify patterns or judgment rules hidden in extensive data sets and construct models with the aid of manual experience adjustment, are a new path for overcoming the limitations of empirical regression models in traditional mix design and predicting the relationship between concrete mix and performance^1–5^. Artificial neural networks (ANNs) are an important algorithm in machine learning, and more and more scholars are using neural networks for engineering prediction and damage identification^6–12^, especially multilayer perceptron (MLP) networks. Nguyen et al.^13^ used four machine learning algorithms, SVR, MLP, GBR, and XGBoost, to predict the compressive and tensile strength of HPC. Comparative studies showed that based on the GBR and XGBoost training models, the performance was better than that of the models based on SVR and MLP for this specific prediction problem. Imran et al.^14^ compared MLR, DT, SVM, BR, and GPR, and found that the GPR model had the highest performance and the smallest prediction error in compressive strength prediction. Wang et al.^15^ trained the artificial neural network through the beetle antenna search algorithm to establish the BAS-MLP concrete strength prediction model, and compared and analyzed it with the SCE-MLP, MVO-MLP coupled algorithm, ANN, and SVM individual learning algorithms. The results showed that combining artificial neural networks with metaheuristic algorithms could better solve such problems. Kovačević et al.^16^ comprehensively reviewed machine learning methods that could be used to estimate SCRC compressive strength, including MLP-ANN, ensemble of MLP-ANN, regression tree ensemble (random forest, boosting, and bagging regression trees), SVR, and GPR. Moodi et al.^17^ studied the effectiveness of three different machine learning methods, RBNN, MLP, and SVR, in predicting the ultimate strength of square and rectangular concrete columns. The results showed that MLP and RBNN obtained higher accuracy and best model prediction performance. Ghunimat et al.^18^ selected three models, MLP, Random Forest Regression, and k-nearest neighbor regression, to estimate the compressive strength of concrete mixtures. By comparing the accuracy and stability of the three methods in predicting compressive strength, it was found that compared with KNN, RFR and MLP had better performance and were the closest to the results. Although the above research has made certain achievements, the initial parameter setting of the neural network has a significant impact on the result. When facing a large or complex nonlinear dataset, the neural network algorithm still has shortcomings such as overfitting and dependence on initial values, and often needs to use optimization algorithms to further improve the accuracy of the prediction results.

Artificial intelligence optimization algorithms are popular parameter optimization algorithms, such as genetic algorithm, particle swarm optimization algorithm, ant colony optimization algorithm, artificial bee colony algorithm, etc. In the research by Karaboga D et al.^19–21^, it has been shown through numerous experiments that the optimization performance of the artificial bee colony algorithm is better than the other aforementioned algorithms, with the characteristics of less control parameters, strong local optimization ability, and fast convergence speed. Currently, scholars have applied this modeling method of using the artificial bee colony algorithm to optimize neural network parameters to predict the mechanical behavior of civil engineering materials and components. Zhou Hao et al.^22^ applied the ABC algorithm and SVM algorithm to optimize the concrete mix proportion, establishing a concrete mix proportion optimization model and verified the rationality and applicability of the model through concrete slump and strength tests. Imran, M et al.^23^ used artificial bee colony (ABC) and cascade forward neural network (CFNN) to optimize and develop a new hybrid model for predicting concrete compressive strength, and model validation using performance indicators showed that the proposed hybrid model was superior to other models in all performance indicators. However, as a random optimization algorithm, the ABC algorithm, similar to other evolutionary algorithms, also has defects of slow convergence speed and easy to fall into local optima. In recent years, various improvement strategies have been proposed for the basic ABC algorithm. Shao Guangcheng et al.^24^ introduced a Gaussian mutation operator in the basic artificial bee colony algorithm to optimize the initial honey source position, designing and establishing an RBF neural network model (IABC-RBF) based on the improved artificial bee colony algorithm, comprehensively improving the prediction ability of neural networks. Leng Xin et al.^25^ improved the artificial bee colony algorithm by introducing global optimal solutions and adaptive judgment factors in two aspects of search method and following bee selection probability. The improved algorithm showed that its convergence speed and accuracy were improved compared to the previous version in three function test results. Yao G et al.^26^ improved the algorithm from three aspects of bee colony initialization, fitness function, and position update formula, overcoming the randomness and easy local optimal solution defects of the initial algorithm. Zhu G et al.^27^, inspired by the particle swarm optimization algorithm, introduced the global optimal solution (gbest) information into the solution search equation, proposing an improved ABC algorithm—GABC algorithm, which improves the algorithm's development rate. Gao W et al.^28^ first used the chaos-based oppositional population initialization method to improve the global convergence, and then combined DE with GABC through evaluation strategies, attempting to use more prior search experience and designed a new method called DGABC to improve the performance of the ABC. These improved ABC algorithms have improved the performance of the algorithm to a certain extent, but have not achieved the adaptive learning of the material's mechanical properties by engineering material test data while avoiding premature convergence of the algorithm, in order to improve the efficiency and accuracy of the material strength prediction model.

Therefore, this paper conducts research on the prediction of compressive strength of concrete and proposes an improved artificial bee colony algorithm. This algorithm improves the two aspects of the bee colony, namely, initialization and honey source update formula. It overcomes the randomness of honey source initialization and the drawback of easily falling into local optimal solutions. The artificial bee colony algorithm is combined with the MLP neural network to enhance the global search capability of the MLP algorithm. Through simulation experiments on two strength prediction datasets, the effectiveness of the algorithm is confirmed, significantly improving optimization efficiency and performance. This algorithm has certain practical value in the actual application of engineering projects.

## Model and optimization algorithm

### MLP neural network

The multilayer perceptron (MLP) is a feedforward supervised learning neural network that includes an input layer, an output layer, and at least one hidden layer. In the MLP model, each layer contains several neurons, and there is no direct connection between neurons in the same layer. The neurons between adjacent layers are fully connected through the addition of weights. Except for the input layer, each layer's neurons have a nonlinear activation function. The multilayer perceptron inputs data through the input layer, and the hidden layer neurons analyze and process the data, and finally, the output layer outputs the results, which achieves multi-layer optimization processing of data.

**Figure 1:**
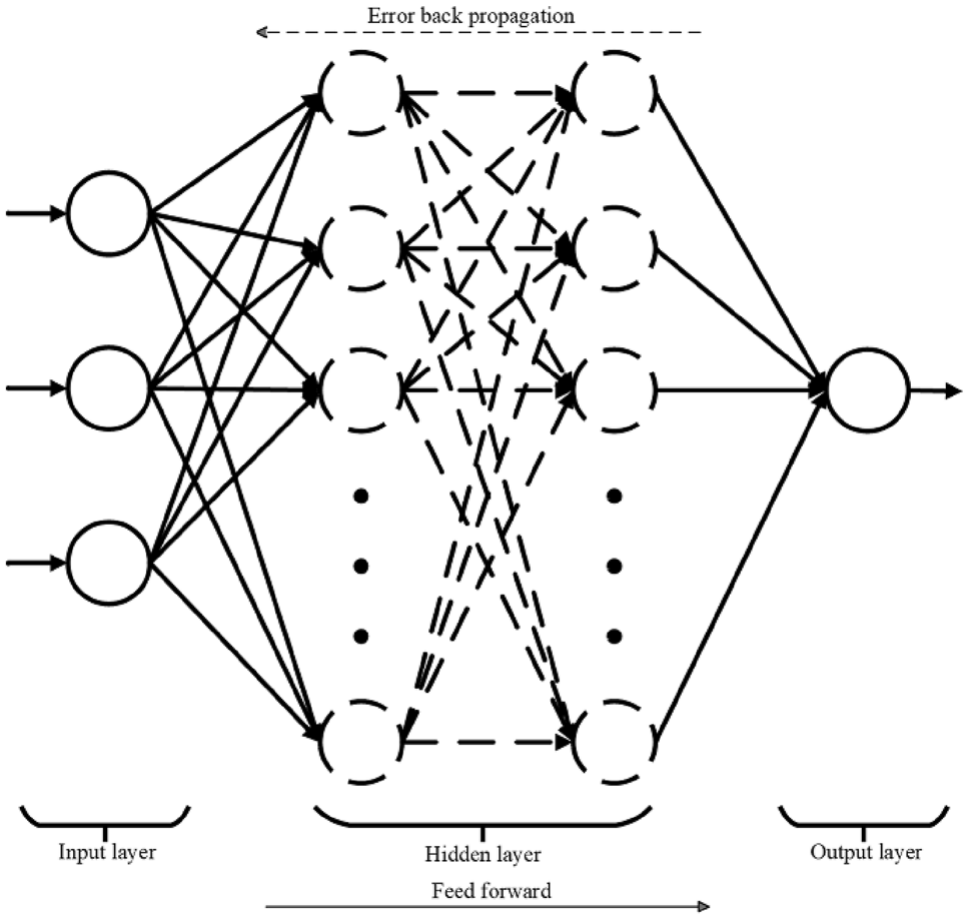
Structure diagram of MLP model.

MLP has excellent capabilities for nonlinear mapping, high parallelism, and high fault tolerance^29,30^. Compared to other machine learning algorithms, it performs well in the presence of noise, nonlinearity, and high-dimensional data. At the same time, it can adapt to specific problem requirements by adjusting the number of layers and nodes in the model. In the research problem of concrete strength prediction, it can predict the compressive strength of concrete by learning the nonlinear mapping relationship between input features and outputs. During the training process, known concrete mix proportions, water-cement ratio, age, and other feature parameters are used as input, and their corresponding compressive strength is used as output. The backpropagation algorithm is used to optimize the network parameters, resulting in a more accurate prediction model. Therefore, compared to other neural network models, the multilayer perceptron has outstanding advantages in nonlinear modeling, training speed, and handling of input variable correlations in concrete strength prediction. Its model structure is shown in Fig. 1. Circles usually represent neurons or nodes, and the connecting lines represent connections between neurons, with arrows on the connecting lines indicating the direction of signal transmission from one neuron to another.

MLP determines the number of neurons in the input and output layers based on the target requirements, while the number of neurons and layers in the hidden layer are determined based on the error requirements set. In the MLP model, the output formula for the $j - th$ neuron in the $i - th$ layer is:

1 $$ y_{j}^{(i)} = f_{j}^{(i)} \left( {W_{j}^{(i)} \cdot y^{(i - 1)} + b_{j}^{(i)} } \right). $$

In the equation, $W_{j}^{(i)}$ is the weight vector of the $j - th$ neuron in the $i - th$ layer, with direction from the ($(i - 1) - th$ layer to the $i - th$ layer and the $j - th$ neuron; $y^{(i - 1)}$ is the output vector of the $(i - 1) - th$ layer; $b_{j}^{(i)}$ is the bias vector of the $j - th$ neuron in the $i - th$ layer; and $f_{j}^{(i)}$ is the activation function of the $j - th$ neuron in the $i - th$ layer.

All the parameters of the MLP model are the weights and bias quantities between each layer. The selection of these parameters affects the prediction performance of MLP to a certain extent. Therefore, it is necessary to optimize the weights and bias quantities of MLP, to make the output of the MLP model closer to the true value, thus improving the prediction accuracy of the model.

### Basic artificial bee colony algorithm

There are currently many algorithms inspired by the behavior of insect colonies in nature, simulating the foraging behavior of insect colonies under the "survival of the fittest" rule. Artificial Bee Colony Algorithm is one of them, evolved from the foraging behavior of bees in nature. In 2005, Professor Karaboga first modeled the behavior and division of labor of bee colonies in foraging in literature and proposed the Artificial Bee Colony Algorithm model. Due to its ease of implementation, few control parameters, and strong stability, the algorithm performs both global and local searches in each iteration, which increases the likelihood of finding the optimal solution. Compared to other swarm intelligence algorithms, it converges faster and quickly gained attention and research from many researchers.

**Figure 2:**
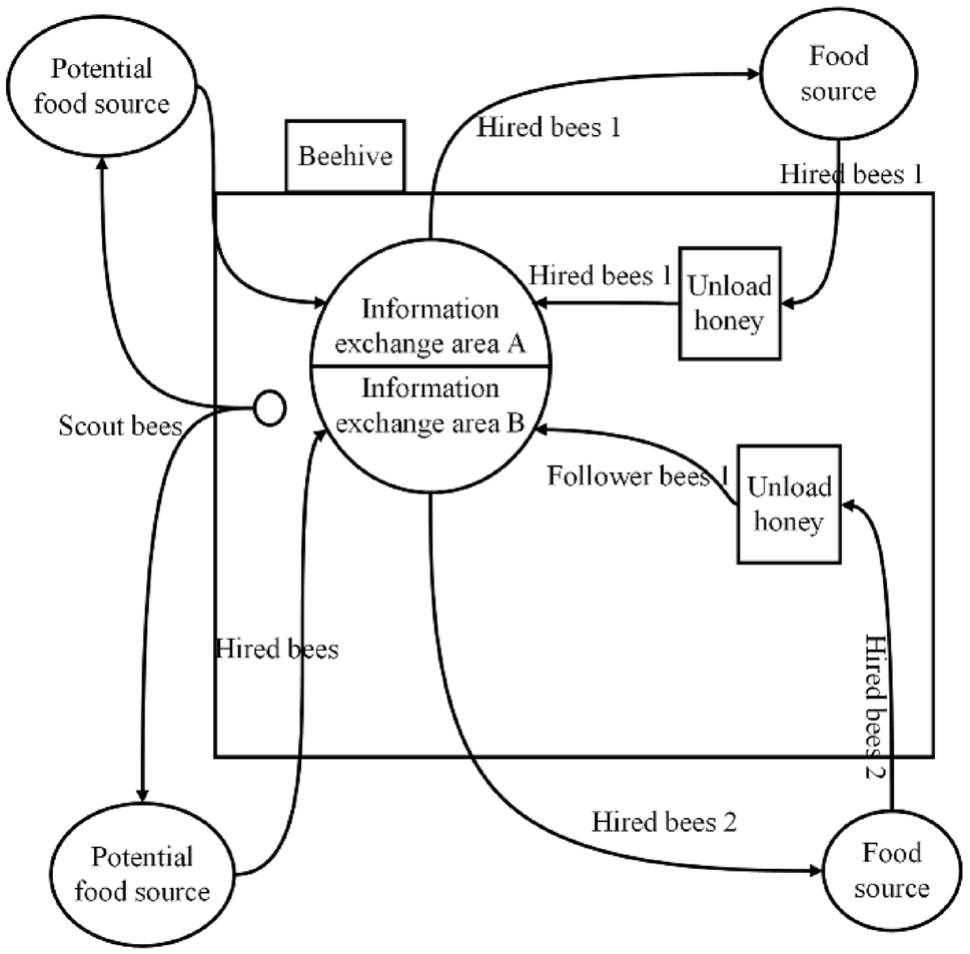
Workflow diagram of the bee colony.

The Artificial Bee Colony Algorithm can simulate the actual honey bee foraging behavior, with the location of the food source representing the solution to the problem, the quality of the pollen representing the fitness of the solution, and the spatial position of a food source representing a set of feasible solutions. The colony is divided into three types of bees: employed bees, which are responsible for randomly searching for food around the hive and carrying information on food sources; follower bees, which follow employed bees to collect nectar; and scout bees, which randomly search for other food sources when a food source has been over-exploited. The individual behavior of bees in the colony is simple, but they coordinate with each other, communicate and cooperate to make the system run smoothly. The workflow is shown in Fig. 2.

The basic model of the ABC algorithm includes four stages: the initialization stage, the employed bee stage, the onlooker bee stage, and the scout bee stage. Below is a detailed introduction to each stage of the artificial bee colony algorithm.

Stage 1: Initialization Phase: set the dimension of the solution space $d$, the size of the bee colony $NP$, the number of leading bees $NP/2$, the control parameter “$limit$” for abandoned food sources, and the maximum number of algorithm iterations Max Iterations. The ABC algorithm randomly generates $NP/2$ initial solutions $x_{i}$, $i = 1 , 2 , ... , NP/2$, each of which is a $d$-dimensional vector, and constructs the fitness function to evaluate the goodness of each food source.

Stage 2: Employed Bee Phase: each honey source represents a solution to the problem being solved. At the beginning, the honey source $v_{i} = (v_{i1} ,v_{i2} ,...,v_{id} )$ can be randomly generated according to the following equation:

2 $$ v_{ij} = L_{ij} + r_{1} \times \left( {U_{ij} - L_{ij} } \right), $$

where $v_{ij}$ represents the $j - th$ variable of the $i - th$ honey source, $i \in \left( {{1 , 2 , }...{ ,} NP/2} \right){ , }j \in \left( {{1 , 2 , }...{ , }d} \right)$, $U_{ij}$ and $L_{ij}$ are the upper and lower bounds of $g_{ij}$, and $r_{1}$ is a random number between 0 and 1, which is used to control the range of the neighborhood.

Stage 3: Follower Bee Phase: Follower bees select the honey source to search next based on the fitness of the honey source, using roulette wheel selection. The selection probability for honey source $i$ is calculated according to the following equation:

3 $$ p_{i} = \frac{{fit_{i} }}{{\sum\limits_{j = 1}^{NP/2} {fit_{j} } }}, $$

where $fit_{i}$ represents the fitness of the $i - th$ solution, which is the amount of nectar of the $i - th$ honey source, and "$p_{i}$" represents the probability of the $i - th$ honey source being selected. As can be seen from the equation, the probability of selecting a food source increases as its fitness increases. Once a follower bee selects a employed bee to follow, it searches in the neighborhood of the food source using Eq. (2) to find a better food source, which corresponds to the optimal fitness.

Stage 4: Scout bee stage: In the bee algorithm, if a local optimum is reached after $t$ iterations of search and the threshold $limit$ for the number of attempts is reached without finding a better quality honey source, the bee responsible for that source becomes a scout bee. The scout bee generates a new honey source according to Eq. (4).

4 $$ v_{i}^{t + 1} = \left\{ {\begin{array}{*{20}c} {L_{i} + r_{1} \times (U_{i} - L_{i} ),} & {t \ge limit} \\ {v_{i}^{t} } & {t < limit} \\ \end{array} } \right.. $$

It can be seen that the ABC algorithm has excellent global search capability and adaptability, and has a wide range of applications in scientific research and industry. It is suitable for predicting the strength of concrete. However, it has weaknesses in terms of slow convergence speed and a tendency to get stuck in local optima.

### Improvements to the artificial bee colony algorithm

In order to enhance the optimization accuracy and convergence speed of the ABC algorithm, this study proposes improvements in two aspects: the optimization of the initial solution space and the honey source search mechanism.

(1) Honey Source Initialization.

The initialization of honey sources has a significant impact on the speed and quality of the solution. In the basic Artificial Bee Colony algorithm, the positions of honey sources are randomly initialized, resulting in an uneven distribution of honey sources in the entire target space. However, chaotic sequences based on chaotic theory possess the properties of traversing the entire space and dynamism. Therefore, using chaotic sequences to improve the initialization of honey sources in the ABC algorithm overcomes the disadvantages of uneven distribution caused by random initialization.

A logistic mapping equation is used to generate chaotic sequences, as shown below:

5 $$ x_{k} = \mu x_{k - 1} \left( {1 - x_{k - 1} } \right). $$

In the equation: $\mu$ represents the growth rate, and when $\mu$ = 4, the system is in a fully chaotic state. $x_{0}$ is the initial value, and $x_{k}$ represents the value of the algorithm at iteration $k$. According to chaotic theory, when the initial value $x_{0}$ is not equal to 0, 0.25, 0.5, 0.75, or 1.0, the sequence is in a chaotic state, which can expand the search range of the algorithm.

After introducing the chaotic sequence, the Eq. (1) can be improved as follows:

6 $$ v_{i} = L_{i} + x_{i} \times \left( {U_{i} - L_{i} } \right), $$

where $i \in \left( {{1 , 2 , }...{\text{ , NP/2}}} \right) \,$,$U_{i}$, and $L_{i}$ are the upper and lower bounds of $g_{i}$; $x_{i}$ represents the $d$-dimensional chaotic sequence after a certain number of iterations.

(2) Honey Source Search Mechanism:

In order to improve the local search ability of the artificial bee colony algorithm, a Gaussian mutation mechanism is introduced. After each iteration, the fitness function values of the honey sources are sorted, and the worst $n \times \eta$ honey sources are selected for Gaussian mutation, where $n$ is the number of honey sources and $\eta$ is the mutation ratio. Empirically, $\eta$ is set to 1/12; $\mu$ is the mean, and $\sigma$ is the standard deviation. After the worst $n \times \eta$ honey sources have been subjected to the Gaussian mutation, the formula for generating a new honey source is as follows:

7 $$ v_{new}^{t} = v_{i}^{t} + Gauss\left( {\mu ,\,\sigma^{2} } \right), $$

where $v_{i}^{t}$ and $v_{new}^{t}$ are the positions of the bee colony before and after mutation, respectively.

**Figure 3:**
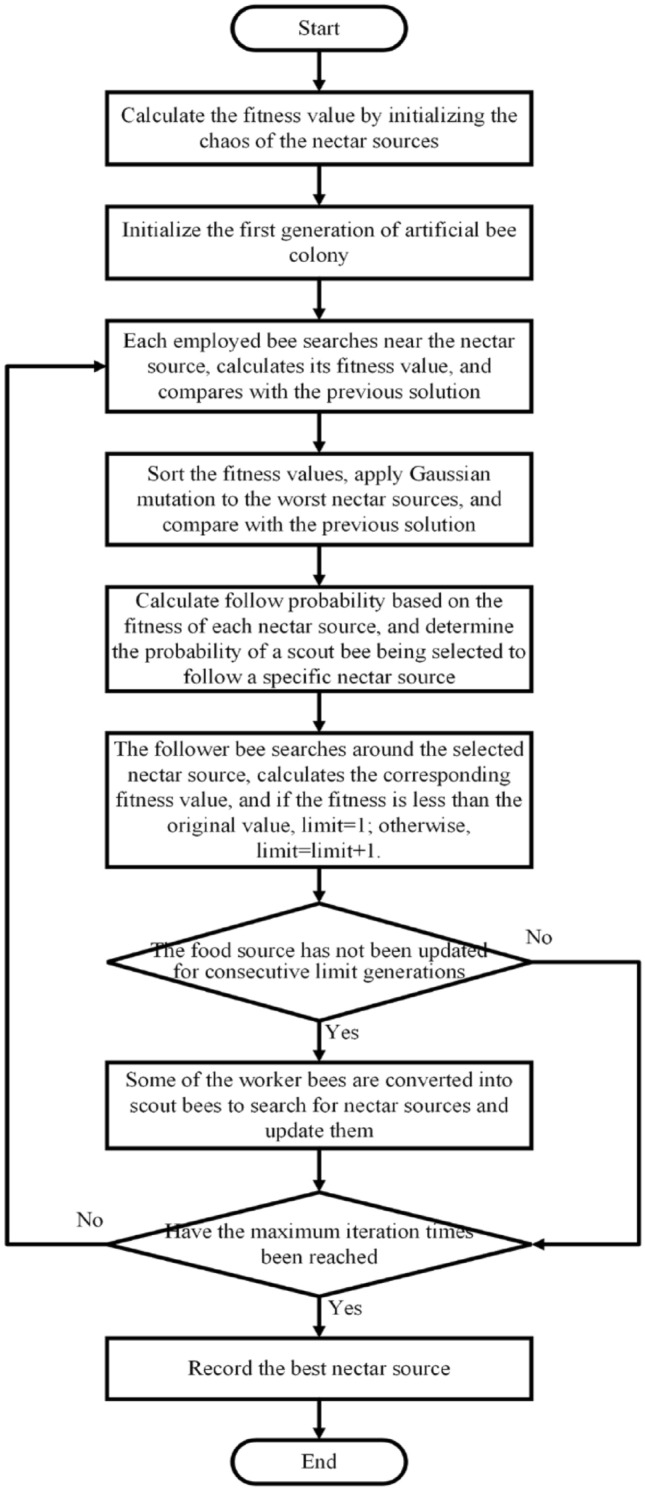
Improved artificial bee colony algorithm flowchart.

The improved artificial bee colony algorithm flow is shown in Fig. 3.

## Strength prediction model based on IABC-MLP

### Model framework for prediction

MLP is a feedforward artificial neural network that has the advantage of processing complex nonlinear relationships and is therefore introduced for predicting the strength of concrete. In the research of predicting the compressive strength of concrete, its prediction results are easily affected by the model structure and fitting ability, which may lead to problems such as underfitting or overfitting, resulting in inaccurate prediction results. Therefore, in this paper, an improved artificial bee colony algorithm is used to search for the optimal initial weights and thresholds of the MLP neural network, which is then applied to the pre-defined network to construct the final algorithmic training model. In this paper, we propose an integrated model for concrete strength prediction which optimizes the Multilayer Perceptron neural network using an improved Artificial Bee Colony algorithm. Compared to MLP neural networks, the proposed IABC-MLP algorithm exhibits advantages in addressing issues such as poor network stability and susceptibility to local optima. In IABC-MLP, the IABC algorithm is used to find the best weights and biases to minimize prediction errors, so that the new model has stronger global search capability and can search for optimal solutions from a wider range of areas, while avoiding the limitations of traditional multilayer perceptron network that rely on the selection of initial weights and gradient descent, thereby improving the accuracy and stability of the model.

Specifically, in the ABC algorithm, individual bees optimize the weights and biases of the MLP through search and selection operations. Each bee represents a solution, which includes a set of values for weights and biases. Bees evaluate solutions based on the performance of the objective function (i.e., prediction error) and perform search and exploration based on a certain probability. The "employed bees" phase in the ABC algorithm is used to improve the current solution through information exchange with other worker bees. The "follower bees" phase is used to search for new solutions and compare them with the current solution for selection. By optimizing the weights and biases of MLP using the ABC algorithm, the performance and prediction accuracy of the model can be improved, thereby achieving more accurate prediction of concrete strength.

**Figure 4:**
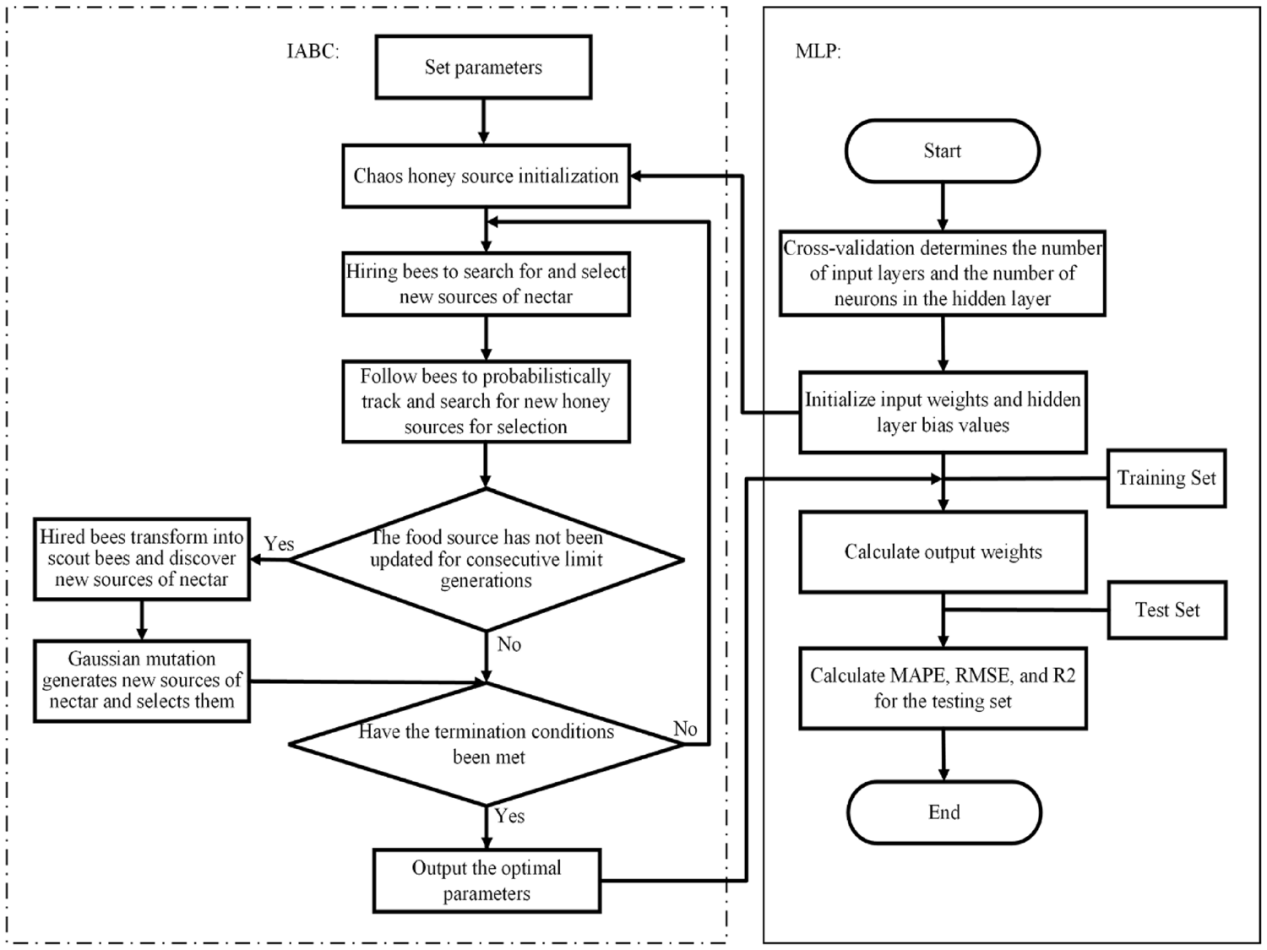
Flowchart of ABC-MLP algorithm.

The specific flow of the IABC-MLP prediction model is shown in Fig. 4.

### Model evaluation index

The evaluation of the accuracy of the model established in this paper is comprehensively assessed using mean absolute error ($MAE$), root mean square error ($RMSE$), and correlation coefficient ($R^{2}$) as performance indicators for model prediction.

$MAE$ represents the average absolute error between the predicted value and the actual value, reflecting the average size of the predicted value error. The smaller the $MAE$ value, the more accurate the prediction of the model. Its expression is as follows:

8 $$ MAE = \frac{1}{n}\sum\limits_{i = 1}^{n} {\left| {y_{i} - \mathop {y_{i} }\limits^{ \wedge } } \right|} . $$

$RMSE$ represents the square root of the mean of the sum of squares of the difference between the predicted value and the true value, and is more sensitive to outliers because it amplifies the square of larger prediction errors. The smaller the value of $RMSE$, the more accurate the prediction of the model. Its expression is as follows:

9 $$ RMSE = \sqrt {\frac{1}{n}\sum\limits_{i = 1}^{n} {\left| {y_{i} - \mathop {y_{i} }\limits^{ \wedge } } \right|}^{2} } . $$

$R^{2}$ indicates the correlation between the predicted result and the true value. The value of $R^{2}$ ranges from 0 to 1, indicating the correlation between the predicted result and the true value. The closer the value is to 1, the better the model effect is. Its expression is as follows:

10 $$ R^{2} = 1 - \frac{{\sum\limits_{i = 1}^{n} {\left( {y_{i} - \mathop {y_{i} }\limits^{ \wedge } } \right)^{2} } }}{{\sum\limits_{i = 1}^{n} {\left( {y_{i} - \overline{y} } \right)^{2} } }}, $$

where: $y_{i}$ is the measured value; $\mathop {y_{i} }\limits^{ \wedge }$ is the predicted value; $\overline{y}$ is the mean; $n$ is the number of measured values.

## Case study analysis

### Data acquisition

Concrete, as a composite material, its compressive strength depends mainly on two factors. Firstly, it depends on the proportion of various materials added during the mixing process, such as cement, slag, ash, water, superplastic, coarse aggregate, and fine aggregate, etc. Secondly, the mixed concrete needs to undergo chemical reactions in the natural environment after mixing in order to harden, so there is a significant correlation between the age of the concrete and its compressive strength.

Based on previous research and analysis of a large number of relevant literature and engineering experience, this paper chooses to consider the influence of concrete material proportion and age on its compressive strength and constructs a compressive strength regression prediction model based on MLP. The computer configuration used in this experiment is as follows: 16 GB of memory, AMD R7 processor, CPU frequency of 3.2 GHz, operating system Windows 11 (64-bit), and Python 3.9 as the programming language.

Dataset 1 is sourced from the Concrete Compressive Strength dataset of the UCI Machine Learning Repository. This dataset consists of 1030 samples, with each sample having 8 influencing factors: cement content, fly ash content, blast furnace slag content, superplasticizer content, water content, coarse aggregate, fine aggregate, and concrete age. Additionally, there is one target output value, which is the compressive strength of the concrete. Table 1 provides descriptive statistical data for Dataset 1.

**Table 1:** Descriptive statistics of concrete compressive strength and key factors in Dataset 1.

Parameters	Descriptive indicators						
	Mean	Median	Standard deviation	Variance	Minimum	Maximum	Skewness
Cement (kg m^−3^)	281.17	272.90	104.46	10,910.98	102.00	540.00	0.51
Slag (kg m^−3)^	73.90	22.00	86.24	7436.90	0.00	359.40	0.80
Ash (kg m^−3^)	54.19	0.00	63.97	4091.64	0.00	200.10	0.54
Water (kg m^−3^)	181.57	185.00	21.34	455.56	121.80	247.00	0.07
Superplastic (kg m^−3^)	6.20	6.40	5.97	35.65	0.00	32.20	0.91
Coarse aggregate (kg m^−3^)	972.92	968.00	77.72	6039.81	801.00	1145.00	-0.04
Fine aggregate (kg m^−3^)	773.58	779.50	80.14	6421.95	594.00	992.60	-0.25
Age (d)	45.66	28.00	63.14	3986.56	1.00	365.00	3.26
Strength (MPa)	35.82	34.45	16.70	278.81	2.33	82.60	0.42

### Input variable selection and data preprocessing

In order to reveal the extent of correlation between each type of input variable and the final output variable, and thereby optimize the input variables to achieve better predictive performance, we selected 8 initial variables from Dataset 1 and calculated their importance indices for the output variable using a random forest algorithm via Python software. The results and importance index rankings are shown in Table 2.

**Table 2:** Feature importance ranking.

Serial number	Features	Importance score	Serial number	Features	Importance score
1	Age	0.335511	5	Superplastic	0.071511
2	Cement	0.321780	6	Fine aggregate	0.036973
3	Water	0.106932	7	Coarse aggregate	0.029245
4	Slag	0.081184	8	Ash	0.016864

As shown in the table, it can be observed that among the input variables, the age of the concrete has the highest correlation with the output variable, which means that the duration of the age has the greatest impact on the compressive strength of the concrete. This is consistent with the empirical experience in engineering. The influence of cement content and water content on the output is secondary, as the cement content, water content, and water-cement ratio, which is one of the important indicators affecting concrete strength, are closely related. Therefore, the relationship between the calculated inputs and outputs based on this method is reasonable.

**Figure 5:**
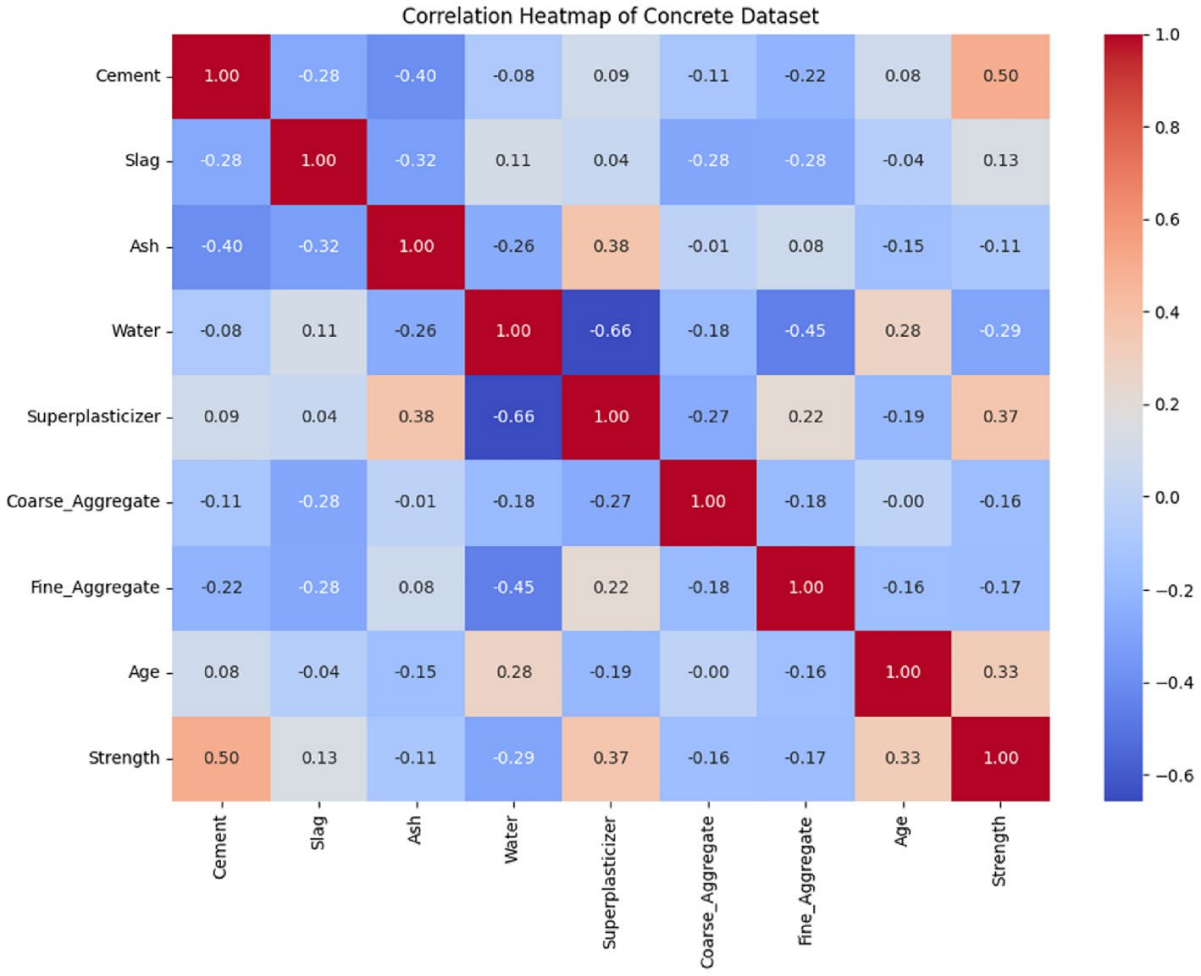
Heatmap of variable correlation.

Meanwhile, the correlation between input variables and the compressive strength of concrete was analyzed using the heatmap method in Python. This analysis aims to validate the importance scores of input variables calculated based on the random forest algorithm. The results are shown in Fig. 5. The variable correlation heatmap describes the correlation between different variables using different coefficient sizes and color depths. The coefficient size indicates the strength of the correlation between two variables, with a larger coefficient indicating a stronger correlation. The color depth represents the direction of the correlation, either positive or negative. A darker color indicates a stronger correlation, while a lighter color indicates a weaker correlation.

As shown in Fig. 5, the correlation coefficient between cement and concrete compressive strength is 0.5, and the color is relatively light red, which indicates that a higher content of cement leads to a higher concrete compressive strength. The correlation coefficient between water and concrete compressive strength is -0.29, and the color is a relatively dark blue, indicating that an increase in water content leads to a decrease in concrete compressive strength. In addition, the effects of cement content, high-efficiency water reducer, water content, and concrete age on concrete compressive strength are apparently higher than those of other variables, which is broadly consistent with the variable importance scores calculated based on the RF algorithm in Table 2, which proves that these four variables have a greater impact on concrete compressive strength.

**Figure 6:**
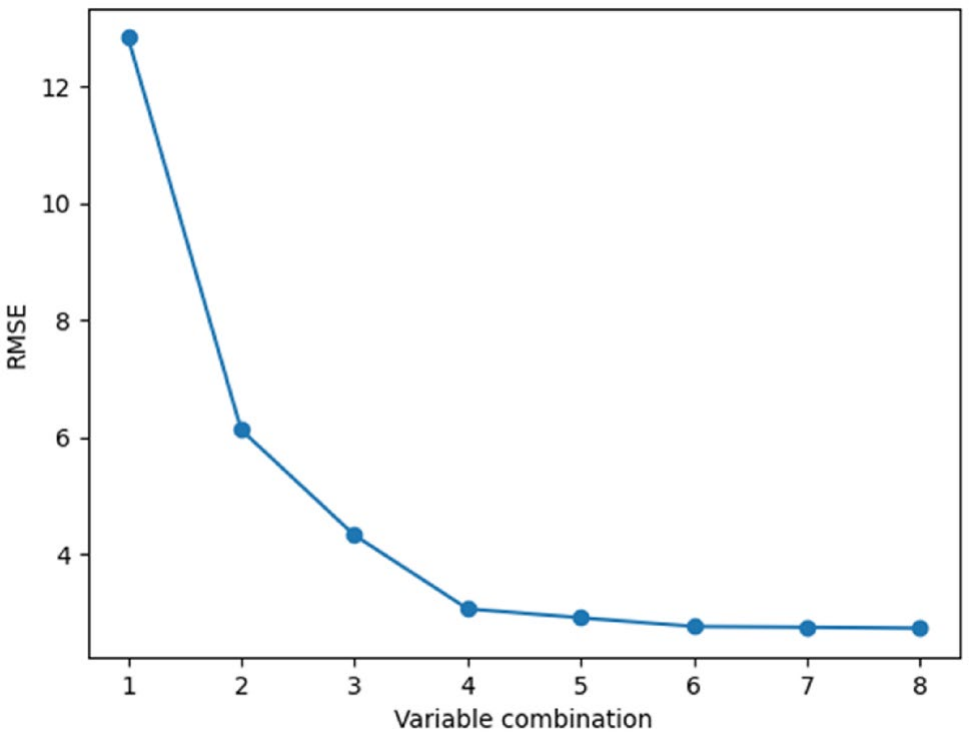
RMSE trend chart of different variable combinations.

By implementing feature selection based on importance ranking on the training set using GridSearchCV method in Pycharm, with RF parameters n_estimators = 500 and max_depth = 9, a trend graph of root mean squared error (RMSE) is obtained for different variable combinations after tenfold cross-validation, as shown in Fig. 6.

From Fig. 6, it can be observed that as important influential factors are sequentially selected from the variable combinations, the overall trend of the model's RMSE shows a gradual decrease. This indicates that the RF algorithm effectively removes some unimportant and redundant influential factors, reduces the deviation between predicted values and actual values, and improves the accuracy of the model's predictions.

After traversing all feature variables, the optimal combination of variables is determined to be 8. At this point, the root mean square error is minimized and the predictive accuracy of the model is highest. Therefore, all 8 influencing factors in Dataset 1 are used as input variables for the concrete compressive strength prediction model.

From the statistical description of the data, it can be observed that there are significant differences in concrete compressive strength and the physical values of various key factors, and the units are not standardized. Therefore, it is necessary to normalize the samples in order to improve the accuracy of the model training. In this paper, the (0, 1) normalization method is selected as the normalization technique, with the following expression:

11 $$ Y = \frac{{X - X_{\min } }}{{X_{\max } - X_{\min } }}. $$

In the formula: $Y$ represents the result of normalization; $X_{\min }$ is the minimum value in the sample; $X_{\max }$ is the maximum value in the sample; $X$ is the sample value that needs to be normalized.

### Parameter settings

For the MLP model in the IABC-MLP model, the initial weights and thresholds are obtained using the most widely used random initialization method. The transfer functions for the hidden layer and output layer are ReLU function and linear function, respectively. In this study, cross-validation is used to determine the number of hidden layers and nodes in the MLP model. By conducting multiple trainings and tests on the training set, the optimal situation is determined where the test mean squared error (MSE) is small. The calculation results show that when the number of hidden layers in the MLP model is 2, and the number of nodes are 50 and 25, respectively, the test error is smaller. To determine the optimization effects of the PSO, ABC, and IABC algorithms on MLP, the number of hidden layer nodes for the PSO-MLP model, ABC-MLP model, and IABC-MLP model remain unchanged. Therefore, the structures of the three prediction models are determined to be 8-50-25-1. The detailed parameters of the MLP model are shown in Table 3.

**Table 3:** MLP model parameters.

Parameter name	Parameter value	Parameter name	Parameter value
Number of layers	4	Learning rate	0.01
Number of hidden layer neurons	50, 25	Batch size	32
Activation function	ReLU	Number OF Iterations	800
Optimization algorithm	Stochastic gradient descent	Train-test split ratio	8: 2

Particle swarm optimization is a heuristic optimization algorithm in which the position and velocity of particles are gradually updated to find the optimal solution to the problem. In this algorithm, the number of particles is specified as 50, indicating that there are 50 particles in the algorithm. The maximum number of iterations for the PSO algorithm is set to 100, which means that the algorithm will terminate after 100 rounds of iteration. In addition, the learning rate and inertia factor are also set to 0.5 and 0.3, respectively, to control the influence of global best position and personal best position on the particle velocity in the algorithm.

Artificial bee colony algorithm is a heuristic algorithm based on the biological characteristics of bee colonies. In this algorithm, the number of bees is set to 50, indicating that 50 bees are used in the algorithm. At the same time, the maximum number of iterations is set to 100, which means that the algorithm will also stop after 100 iterations. The improved artificial bee colony algorithm improves the algorithm in two aspects: optimizing the initial solution space and honey source search mechanism, and the parameter settings are the same as those for the standard ABC algorithm.

## Model comparison

### Comparison with the coupled model

The particle swarm optimization (PSO) algorithm is a swarm intelligence algorithm that imitates the collaboration between particles to search for optimal solutions. Each particle updates its position and velocity continuously by interacting with other particles until the optimal solution is found. In the PSO algorithm, the interaction information between particles includes the current position, velocity, and the optimal position. Similar to the artificial bee colony (ABC) algorithm, the PSO algorithm can simultaneously consider global and local optimal solutions, and converge to the optimal solution quickly. Therefore, to verify the effectiveness of the IABC-MLP coupled model, we compared the IABC algorithm proposed in this paper with the basic ABC algorithm and the PSO algorithm.

**Figure 7:**
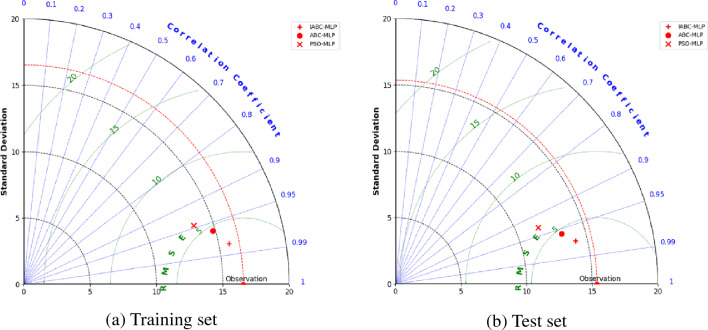
Taylor diagram for model comparison.

The normalized concrete strength training set was input into the MLP for training, and three concrete strength prediction models were established by optimizing the weights and biases of the MLP using the particle swarm algorithm, artificial bee colony algorithm, and improved artificial bee colony algorithm, respectively. In order to select the best strength prediction model with a clearer comparison, this study used a Taylor diagram to evaluate and select the models. Figure 7 displays the Taylor diagram of the three prediction models evaluating the accuracy of the training and test sets, providing an intuitive comparison of the performance of the three machine learning models in concrete strength prediction tasks. The scatter points in the figure represent the machine learning models, the red dashed line represents the observation line, the circular arc centered on the origin represents the standard deviation SD, the radiating line represents the correlation coefficient R, and the circular arc centered on the observation point represents the root mean square error RMSE. The coordinates of the training set and test set observation points reach the ideal state, i.e., R = 1, RMSE = 0. The standard deviation SD of the training set is 16.539, which is consistent with the standard deviation of the compressive strength sample of the verification set. The standard deviation SD of the test set is 15.356, which is consistent with the standard deviation of the compressive strength sample of the test set.

Based on the distribution of the training and test set models displayed in the Taylor diagram (Fig. 7), the following results can be observed: the validation set and test set R values of the three models are all above 0.9, indicating that there is a strong correlation between the predicted output of the model and the actual output. By comparing the rays between each scatter point and the coordinate origin, as well as the angles formed between the scatter points and the vertical coordinate axis, it can be concluded that R_IABC-MLP_ > R_ABC-MLP_ > R_PSO-MLP_. By comparing the straight-line distances between each scatter point and the observation point in the diagram, it can be concluded that the IABC-MLP model has the closest distance to the observation point, followed by ABC-MLP, and then PSO-MLP, indicating that RMSE_IABC-MLP_ < RMSE_ABC-MLP_ < RMSE_PSO-MLP_. Therefore, in this analysis, the IABC-MLP model performed the best, and the PSO-MLP model performed the worst.

To further analyze the prediction evaluation indicators of various coupling models and ensure the reliability of the results, this study adopted the method of multiple runs and carried out 30 independent computations, and provided the average statistical results. In order to further verify the quality of the research, this study compared the predictive performance of our constructed model with representative concrete strength prediction models published in the literature. Specifically, we chose the TSO-ELM model constructed by Zhang et al.^31^ and the CS-AdaBoost-CART model constructed by Xue et al.^32^ for comparison. The evaluation indicator results for the prediction set are shown in Fig. 4.

According to the evaluation indicators shown in Table 4, all types of machine learning models constructed in this article have been tested and fully reflect their predictive performance. It is worth noting that the IABC-MLP model exhibited the best predictive ability in all indicator evaluations, specifically with a goodness of fit above 97%, resulting in a 1.5% and 4.2% increase in fitting ability compared to the ABC-MLP and PSO-MLP models, respectively. In addition, the IABC-MLP also performed well in performance indicators such as mean absolute error and root mean square error, making it better suited to meeting the requirements of fitting accuracy for concrete strength prediction. It is worth mentioning that compared with the evaluation indicator results of two models proposed by other research results, the IABC-MLP model is the best model for predicting concrete compressive strength, achieving the optimal level in indicators such as MAE, RMSE, and $R^{2}$. In conclusion, in this research field, the IABC-MLP model exhibited outstanding fitting ability and is expected to provide highly valuable references for related research.

**Table 4:** Results of evaluation indicators for each coupling model.

Model	Evaluation metrics		
	$MAE$/MPa	$RMSE$/MPa	$R^{2}$
IABC-MLP	2.504	3.408	0.973
ABC-MLP	3.053	4.070	0.958
PSO-MLP	3.799	5.070	0.931
TSO-ELM	5.240	6.050	0.870
CS-AdaBoost-CART	3.996	4.987	0.914

**Figure 8:**
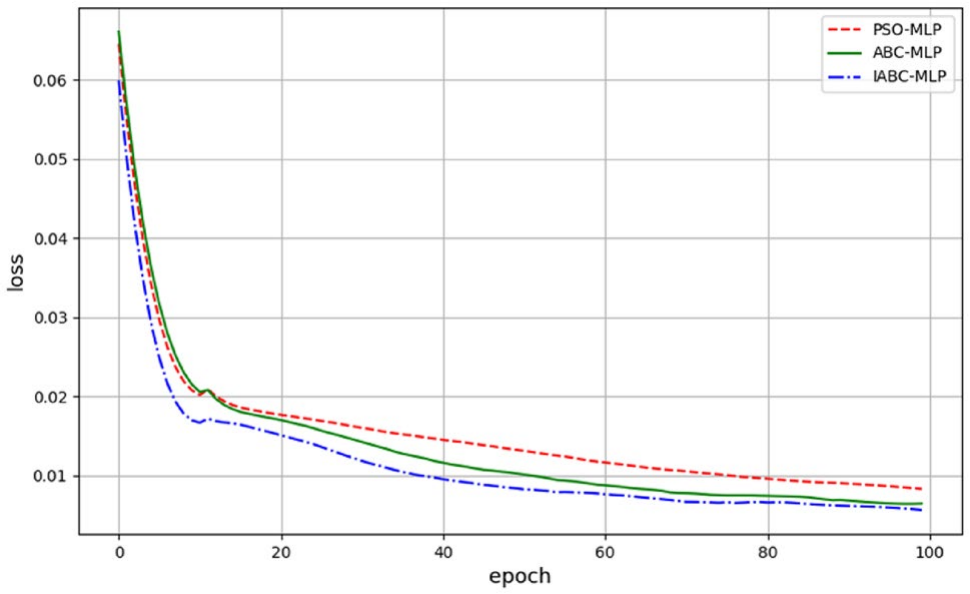
Loss iteration graph.

Convergence speed is an important indicator of the performance of optimization algorithms. Figure 8 shows the convergence curves of PSO-MLP, ABC-MLP, and IABC-MLP algorithms. In the figure, epoch represents the iteration times and loss represents the loss value. Comparing the loss values of the three coupled model algorithms after each iteration, it can be seen that the original ABC algorithm has a certain degree of slow convergence speed and is prone to getting stuck in local optimal solutions. Compared with the original algorithm, the PSO algorithm has faster convergence speed and fewer iterations, but weaker global optimization ability. This paper's IABC algorithm adopted the optimization of initial solution space and honey source search mechanism to avoid problems such as the randomness of initialization of food source positions and being prone to getting stuck in local optimal solutions. The algorithm enables bees to quickly move to the region where the optimal food source is located through the Gaussian mutation operator. Therefore, the IABC algorithm has significantly improved in both iteration speed and global optimization ability. After about 100 iterations, the test error tends to stabilize. Compared with the ABC-MLP algorithm and the PSO-MLP algorithm, the IABC-MLP neural network algorithm is superior in both optimization accuracy and convergence speed in the same number of iterations and can well predict the compressive strength performance of concrete.

### Comparison with single model

**Figure 9:**
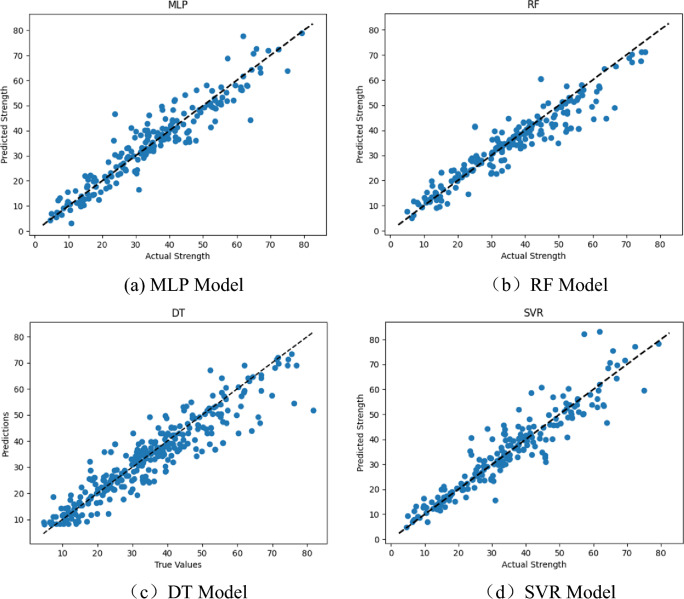
Fitting diagram of predicted values and actual values of each model.

To further observe the prediction effect of the IABC-MLP model, this study also selected four single models for concrete strength prediction experiments: Multi-Layer Perceptron (MLP), decision tree (DT), Support Vector Regression (SVR), and Random Forest (RF). Figure 9 shows the fitting effect of the predicted values and actual values of each model, where it can be visually seen that the DT model has the worst fitting effect compared to other models, and the sample points are not concentrated on the regression line. The performance of all models was measured using evaluation metrics such as mean absolute error, root mean squared error, and correlation coefficient. The calculation results of all models are shown in Table 5.

**Table 5:** Evaluation metrics results of each single model.

Model	Evaluation metrics		
	$MAE$/MPa	$RMSE$/MPa	$R^{2}$
MLP	3.837	5.183	0.898
DT	4.340	6.151	0.860
SVR	3.901	5.814	0.872
RF	3.738	5.472	0.884

From Table 5, it can be seen that the goodness of fit values of the four single models from highest to lowest are: MLP > RF > SVR > DT. Compared to the single MLP model, the ABC-MLP model increased the $R^{2}$ value by 8.35%. This means that ABC adapts and optimizes the model based on the characteristics of the data, overcoming the curse of dimensionality and improving the prediction accuracy and stability of the MLP model.

In addition, compared to the other three classical models, the IABC-MLP model shows significantly better prediction performance. The reason is that DT, SVR, and RF are individual learning algorithms, which require a large number of weights and thresholds when dealing with a large number of samples, resulting in low accuracy. On the other hand, IABC-MLP is a coupled metaheuristic algorithm that does not require excessive parameter tuning. It has a fast convergence speed and strong global optimization ability, enabling accurate prediction of concrete compressive strength.

## Model performance verification

In order to further validate the generalization performance of the IABC-MLP neural network model and test its feasibility in practical construction, a new Dataset 2 is selected for validation. It is applied to the dataset publicly available in the paper by Al-Shamiri^33^, which consists of 324 sets of concrete samples obtained in their laboratory. This dataset has fewer types of input variables, so there is no need for variable optimization. The input variables are the cement, superplastic, water, coarse aggregate, fine aggregate, with the concrete compressive strength as the output variable.

This dataset is used to verify the generalization of the proposed model in this paper, and its descriptive statistical data are shown in Table 6.

**Table 6:** Descriptive statistics of concrete compressive strength and key factors for Dataset 2.

Parameters	Descriptive indicators						
	Mean	Median	Standard deviation	Variance	Minimum	Maximum	Skewness
Cement (kg m^−3^)	417.81	411	76.91	5914.77	284	600	0.41
Water (kg m^−3^)	170	170	8.16	66.67	160	180	0
Superplastic (kg m^−3^)	0.90	1.0	0.56	0.32	0.00	2.0	0.09
Coarse aggregate (kg m^−3^)	898.51	898.00	43.75	1914.08	845.00	989.00	0.02
Fine aggregate (kg m^−3^)	767.63	769.5	85.39	7291.25	552.00	951.00	− 0.19
Strength (MPa)	51.93	48.90	9.43	88.95	37.5	73.6	0.44

**Figure 10:**
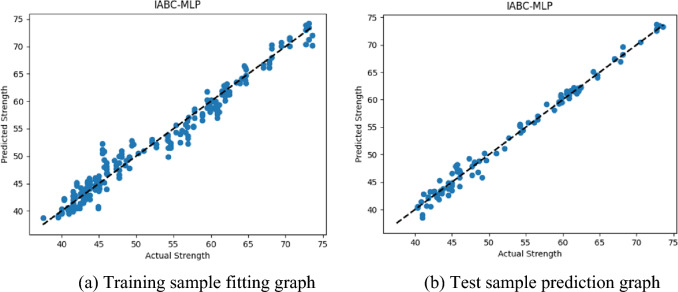
IABC-MLP strength fitting and prediction graphs.

The training sample fitting and test sample prediction are shown in Fig. 10.

From Fig. 10, it can be seen that for the training set, the data points in the scatter plot of the MLP model optimized by the improved artificial bee colony algorithm are closely distributed around the regression line, indicating that the model can fit the training data well. For the test set, the data points of the model further converge, indicating that the model not only performs well on the training set, but also has higher accuracy and generalization ability on the unseen test data. According to the IABC-MLP evaluation index results in Table 7, it can be analyzed that the model for predicting the compressive strength of concrete performs well. Firstly, the value of the MAE evaluation index is small, indicating that the average error between the predicted value and the actual value is small, and the model has good fitting effect. Secondly, the value of the RMSE evaluation index is also small, indicating that the dispersion degree of predicted values and actual values of the model is small, and the prediction accuracy is high. Finally, the results of the $R^{2}$ evaluation index are 0.977 and 0.986, showing high accuracy in both the training set and the test set, indicating that the model has good fitting ability and strong prediction ability. In conclusion, by observing the IABC-MLP strength fitting effect diagram and analyzing the evaluation index results, it can be clearly seen that the established model has high prediction accuracy and reliability in the study of predicting the compressive strength of concrete, can effectively predict the compressive strength of concrete, provide important reference for engineering design and construction, and is expected to improve engineering quality and safety.

**Table 7:** Evaluation indicator results for IABC-MLP.

Evaluation metrics	Training set	Test set
$MAE$/MPa	1.215	0.796
$RMSE$/MPa	1.553	1.211
$R^{2}$	0.977	0.986

## Conclusion

Given the high requirements for real-time and accuracy in predicting the compressive strength of concrete at construction sites, this study uses the multilayer perceptron with higher predictive fitting quality as the modeling method. Due to the significant impact of parameters on the prediction performance of MLP, the process of optimizing hyperparameters is time-consuming. In this study, the basic artificial bee colony algorithm is improved by introducing a Gaussian mutation operator and optimizing the initial solution space, and an MLP neural network model based on the improved artificial bee colony algorithm is established. Through simulating and modeling the data, the proposed IABC-MLP is validated, and the following conclusions are drawn:

1. To explore the parameter optimization problem of the MLP neural network, we used three metaheuristic algorithms, namely PSO, ABC, and IABC, and analyzed them using three evaluation indicators for the three coupled models. The results show that the fitting quality of IABC-MLP can reach above 97%. Compared to ABC-MLP and PSO-MLP, it is 1.5% and 4.2% higher, respectively, enabling a quicker completion of the prediction process and a more accurate prediction of the compressive strength of concrete.
2. Comparing the IABC-MLP algorithm with four common individual learning algorithms, namely MLP, DT, SVR, and RF, the study found that the IABC-MLP model improved the $R^{2}$ value by 8.35% compared to the single MLP model. In other words, IABC achieves an adaptive adjustment and optimization of the model based on the characteristics of the data, overcomes the curse of dimensionality problem in high-dimensional data, and enhances the prediction accuracy and stability of the MLP model.
3. The newly established IABC-MLP prediction model for the compressive strength of concrete is experimentally simulated on another publicly available dataset. The fitting accuracy reaches 99.2%, demonstrating the good generalization ability of the model and its suitability for predicting the compressive strength of concrete in actual construction sites.

In summary, the proposed IABC-MLP model in this study can quickly complete the prediction process, accurately predict the compressive strength of concrete, and has good generalization ability, making it suitable for predicting the compressive strength of concrete in actual construction sites. However, the prediction study in this paper mainly focuses on predicting the compressive strength of concrete under standard conditions at room temperature. It is also important to further research the practical application of predicting the compressive strength of concrete under different temperatures, water contents, and other conditions.

### Supplementary Information

Supplementary Information.

## Data Availability

Some or all data, models, or code that support the findings of this study are available from the corresponding author upon reasonable request.
