# Supplementary material for: Prediction of compressive strength of concrete based on improved artificial bee colony-multilayer perceptron algorithm
Source: Sci Rep. 2024 Mar 17;14:6414. doi: 10.1038/s41598-024-57131-w (PMC10944844; doi:10.1038/s41598-024-57131-w)
Supplement: Supplementary file 1 — Supplementary Information. [file 41598_2024_57131_MOESM1_ESM.docx]

**Dataset 1**

| number | cement | slag | ash | | water | | superplastic | | coarse_aggregate | | fine_aggregate | | age | | strength | | |
| --- | --- | --- | --- | --- | --- | --- | --- | --- | --- | --- | --- | --- | --- | --- | --- | --- | --- |
| 1 | 540 | 0 | | 0 | | 162 | | 2.5 | | 1040 | | 676 | | 28 | | 79.99 |  |
| 2 | 540 | 0 | | 0 | | 162 | | 2.5 | | 1055 | | 676 | | 28 | | 61.89 |  |
| 3 | 332.5 | 142.5 | | 0 | | 228 | | 0 | | 932 | | 594 | | 270 | | 40.27 |  |
| 4 | 332.5 | 142.5 | | 0 | | 228 | | 0 | | 932 | | 594 | | 365 | | 41.05 |  |
| 5 | 198.6 | 132.4 | | 0 | | 192 | | 0 | | 978.4 | | 825.5 | | 360 | | 44.3 |  |
| 6 | 266 | 114 | | 0 | | 228 | | 0 | | 932 | | 670 | | 90 | | 47.03 |  |
| 7 | 380 | 95 | | 0 | | 228 | | 0 | | 932 | | 594 | | 365 | | 43.7 |  |
| 8 | 380 | 95 | | 0 | | 228 | | 0 | | 932 | | 594 | | 28 | | 36.45 |  |
| 9 | 266 | 114 | | 0 | | 228 | | 0 | | 932 | | 670 | | 28 | | 45.85 |  |
| 10 | 475 | 0 | | 0 | | 228 | | 0 | | 932 | | 594 | | 28 | | 39.29 |  |
| 11 | 198.6 | 132.4 | | 0 | | 192 | | 0 | | 978.4 | | 825.5 | | 90 | | 38.07 |  |
| 12 | 198.6 | 132.4 | | 0 | | 192 | | 0 | | 978.4 | | 825.5 | | 28 | | 28.02 |  |
| 13 | 427.5 | 47.5 | | 0 | | 228 | | 0 | | 932 | | 594 | | 270 | | 43.01 |  |
| 14 | 190 | 190 | | 0 | | 228 | | 0 | | 932 | | 670 | | 90 | | 42.33 |  |
| 15 | 304 | 76 | | 0 | | 228 | | 0 | | 932 | | 670 | | 28 | | 47.81 |  |
| 16 | 380 | 0 | | 0 | | 228 | | 0 | | 932 | | 670 | | 90 | | 52.91 |  |
| 17 | 139.6 | 209.4 | | 0 | | 192 | | 0 | | 1047 | | 806.9 | | 90 | | 39.36 |  |
| 18 | 342 | 38 | | 0 | | 228 | | 0 | | 932 | | 670 | | 365 | | 56.14 |  |
| 19 | 380 | 95 | | 0 | | 228 | | 0 | | 932 | | 594 | | 90 | | 40.56 |  |
| 20 | 475 | 0 | | 0 | | 228 | | 0 | | 932 | | 594 | | 180 | | 42.62 |  |
| 21 | 427.5 | 47.5 | | 0 | | 228 | | 0 | | 932 | | 594 | | 180 | | 41.84 |  |
| 22 | 139.6 | 209.4 | | 0 | | 192 | | 0 | | 1047 | | 806.9 | | 28 | | 28.24 |  |
| 23 | 139.6 | 209.4 | | 0 | | 192 | | 0 | | 1047 | | 806.9 | | 3 | | 8.06 |  |
| 24 | 139.6 | 209.4 | | 0 | | 192 | | 0 | | 1047 | | 806.9 | | 180 | | 44.21 |  |
| 25 | 380 | 0 | | 0 | | 228 | | 0 | | 932 | | 670 | | 365 | | 52.52 |  |
| 26 | 380 | 0 | | 0 | | 228 | | 0 | | 932 | | 670 | | 270 | | 53.3 |  |
| 27 | 380 | 95 | | 0 | | 228 | | 0 | | 932 | | 594 | | 270 | | 41.15 |  |
| 28 | 342 | 38 | | 0 | | 228 | | 0 | | 932 | | 670 | | 180 | | 52.12 |  |
| 29 | 427.5 | 47.5 | | 0 | | 228 | | 0 | | 932 | | 594 | | 28 | | 37.43 |  |
| 30 | 475 | 0 | | 0 | | 228 | | 0 | | 932 | | 594 | | 7 | | 38.6 |  |
| 31 | 304 | 76 | | 0 | | 228 | | 0 | | 932 | | 670 | | 365 | | 55.26 |  |
| 32 | 266 | 114 | | 0 | | 228 | | 0 | | 932 | | 670 | | 365 | | 52.91 |  |
| 33 | 198.6 | 132.4 | | 0 | | 192 | | 0 | | 978.4 | | 825.5 | | 180 | | 41.72 |  |
| 34 | 475 | 0 | | 0 | | 228 | | 0 | | 932 | | 594 | | 270 | | 42.13 |  |
| 35 | 190 | 190 | | 0 | | 228 | | 0 | | 932 | | 670 | | 365 | | 53.69 |  |
| 36 | 237.5 | 237.5 | | 0 | | 228 | | 0 | | 932 | | 594 | | 270 | | 38.41 |  |
| 37 | 237.5 | 237.5 | | 0 | | 228 | | 0 | | 932 | | 594 | | 28 | | 30.08 |  |
| 38 | 332.5 | 142.5 | | 0 | | 228 | | 0 | | 932 | | 594 | | 90 | | 37.72 |  |
| 39 | 475 | 0 | | 0 | | 228 | | 0 | | 932 | | 594 | | 90 | | 42.23 |  |
| 40 | 237.5 | 237.5 | | 0 | | 228 | | 0 | | 932 | | 594 | | 180 | | 36.25 |  |
| 41 | 342 | 38 | | 0 | | 228 | | 0 | | 932 | | 670 | | 90 | | 50.46 |  |
| 42 | 427.5 | 47.5 | | 0 | | 228 | | 0 | | 932 | | 594 | | 365 | | 43.7 |  |
| 43 | 237.5 | 237.5 | | 0 | | 228 | | 0 | | 932 | | 594 | | 365 | | 39 |  |
| 44 | 380 | 0 | | 0 | | 228 | | 0 | | 932 | | 670 | | 180 | | 53.1 |  |
| 45 | 427.5 | 47.5 | | 0 | | 228 | | 0 | | 932 | | 594 | | 90 | | 41.54 |  |
| 46 | 427.5 | 47.5 | | 0 | | 228 | | 0 | | 932 | | 594 | | 7 | | 35.08 |  |
| 47 | 349 | 0 | | 0 | | 192 | | 0 | | 1047 | | 806.9 | | 3 | | 15.05 |  |
| 48 | 380 | 95 | | 0 | | 228 | | 0 | | 932 | | 594 | | 180 | | 40.76 |  |
| 49 | 237.5 | 237.5 | | 0 | | 228 | | 0 | | 932 | | 594 | | 7 | | 26.26 |  |
| 50 | 380 | 95 | | 0 | | 228 | | 0 | | 932 | | 594 | | 7 | | 32.82 |  |
| 51 | 332.5 | 142.5 | | 0 | | 228 | | 0 | | 932 | | 594 | | 180 | | 39.78 |  |
| 52 | 190 | 190 | | 0 | | 228 | | 0 | | 932 | | 670 | | 180 | | 46.93 |  |
| 53 | 237.5 | 237.5 | | 0 | | 228 | | 0 | | 932 | | 594 | | 90 | | 33.12 |  |
| 54 | 304 | 76 | | 0 | | 228 | | 0 | | 932 | | 670 | | 90 | | 49.19 |  |
| 55 | 139.6 | 209.4 | | 0 | | 192 | | 0 | | 1047 | | 806.9 | | 7 | | 14.59 |  |
| 56 | 198.6 | 132.4 | | 0 | | 192 | | 0 | | 978.4 | | 825.5 | | 7 | | 14.64 |  |
| 57 | 475 | 0 | | 0 | | 228 | | 0 | | 932 | | 594 | | 365 | | 41.93 |  |
| 58 | 198.6 | 132.4 | | 0 | | 192 | | 0 | | 978.4 | | 825.5 | | 3 | | 9.13 |  |
| 59 | 304 | 76 | | 0 | | 228 | | 0 | | 932 | | 670 | | 180 | | 50.95 |  |
| 60 | 332.5 | 142.5 | | 0 | | 228 | | 0 | | 932 | | 594 | | 28 | | 33.02 |  |
| 61 | 304 | 76 | | 0 | | 228 | | 0 | | 932 | | 670 | | 270 | | 54.38 |  |
| 62 | 266 | 114 | | 0 | | 228 | | 0 | | 932 | | 670 | | 270 | | 51.73 |  |
| 63 | 310 | 0 | | 0 | | 192 | | 0 | | 971 | | 850.6 | | 3 | | 9.87 |  |
| 64 | 190 | 190 | | 0 | | 228 | | 0 | | 932 | | 670 | | 270 | | 50.66 |  |
| 65 | 266 | 114 | | 0 | | 228 | | 0 | | 932 | | 670 | | 180 | | 48.7 |  |
| 66 | 342 | 38 | | 0 | | 228 | | 0 | | 932 | | 670 | | 270 | | 55.06 |  |
| 67 | 139.6 | 209.4 | | 0 | | 192 | | 0 | | 1047 | | 806.9 | | 360 | | 44.7 |  |
| 68 | 332.5 | 142.5 | | 0 | | 228 | | 0 | | 932 | | 594 | | 7 | | 30.28 |  |
| 69 | 190 | 190 | | 0 | | 228 | | 0 | | 932 | | 670 | | 28 | | 40.86 |  |
| 70 | 485 | 0 | | 0 | | 146 | | 0 | | 1120 | | 800 | | 28 | | 71.99 |  |
| 71 | 374 | 189.2 | | 0 | | 170.1 | | 10.1 | | 926.1 | | 756.7 | | 3 | | 34.4 |  |
| 72 | 313.3 | 262.2 | | 0 | | 175.5 | | 8.6 | | 1046.9 | | 611.8 | | 3 | | 28.8 |  |
| 73 | 425 | 106.3 | | 0 | | 153.5 | | 16.5 | | 852.1 | | 887.1 | | 3 | | 33.4 |  |
| 74 | 425 | 106.3 | | 0 | | 151.4 | | 18.6 | | 936 | | 803.7 | | 3 | | 36.3 |  |
| 75 | 375 | 93.8 | | 0 | | 126.6 | | 23.4 | | 852.1 | | 992.6 | | 3 | | 29 |  |
| 76 | 475 | 118.8 | | 0 | | 181.1 | | 8.9 | | 852.1 | | 781.5 | | 3 | | 37.8 |  |
| 77 | 469 | 117.2 | | 0 | | 137.8 | | 32.2 | | 852.1 | | 840.5 | | 3 | | 40.2 |  |
| 78 | 425 | 106.3 | | 0 | | 153.5 | | 16.5 | | 852.1 | | 887.1 | | 3 | | 33.4 |  |
| 79 | 388.6 | 97.1 | | 0 | | 157.9 | | 12.1 | | 852.1 | | 925.7 | | 3 | | 28.1 |  |
| 80 | 531.3 | 0 | | 0 | | 141.8 | | 28.2 | | 852.1 | | 893.7 | | 3 | | 41.3 |  |
| 81 | 425 | 106.3 | | 0 | | 153.5 | | 16.5 | | 852.1 | | 887.1 | | 3 | | 33.4 |  |
| 82 | 318.8 | 212.5 | | 0 | | 155.7 | | 14.3 | | 852.1 | | 880.4 | | 3 | | 25.2 |  |
| 83 | 401.8 | 94.7 | | 0 | | 147.4 | | 11.4 | | 946.8 | | 852.1 | | 3 | | 41.1 |  |
| 84 | 362.6 | 189 | | 0 | | 164.9 | | 11.6 | | 944.7 | | 755.8 | | 3 | | 35.3 |  |
| 85 | 323.7 | 282.8 | | 0 | | 183.8 | | 10.3 | | 942.7 | | 659.9 | | 3 | | 28.3 |  |
| 86 | 379.5 | 151.2 | | 0 | | 153.9 | | 15.9 | | 1134.3 | | 605 | | 3 | | 28.6 |  |
| 87 | 362.6 | 189 | | 0 | | 164.9 | | 11.6 | | 944.7 | | 755.8 | | 3 | | 35.3 |  |
| 88 | 286.3 | 200.9 | | 0 | | 144.7 | | 11.2 | | 1004.6 | | 803.7 | | 3 | | 24.4 |  |
| 89 | 362.6 | 189 | | 0 | | 164.9 | | 11.6 | | 944.7 | | 755.8 | | 3 | | 35.3 |  |
| 90 | 439 | 177 | | 0 | | 186 | | 11.1 | | 884.9 | | 707.9 | | 3 | | 39.3 |  |
| 91 | 389.9 | 189 | | 0 | | 145.9 | | 22 | | 944.7 | | 755.8 | | 3 | | 40.6 |  |
| 92 | 362.6 | 189 | | 0 | | 164.9 | | 11.6 | | 944.7 | | 755.8 | | 3 | | 35.3 |  |
| 93 | 337.9 | 189 | | 0 | | 174.9 | | 9.5 | | 944.7 | | 755.8 | | 3 | | 24.1 |  |
| 94 | 374 | 189.2 | | 0 | | 170.1 | | 10.1 | | 926.1 | | 756.7 | | 7 | | 46.2 |  |
| 95 | 313.3 | 262.2 | | 0 | | 175.5 | | 8.6 | | 1046.9 | | 611.8 | | 7 | | 42.8 |  |
| 96 | 425 | 106.3 | | 0 | | 153.5 | | 16.5 | | 852.1 | | 887.1 | | 7 | | 49.2 |  |
| 97 | 425 | 106.3 | | 0 | | 151.4 | | 18.6 | | 936 | | 803.7 | | 7 | | 46.8 |  |
| 98 | 375 | 93.8 | | 0 | | 126.6 | | 23.4 | | 852.1 | | 992.6 | | 7 | | 45.7 |  |
| 99 | 475 | 118.8 | | 0 | | 181.1 | | 8.9 | | 852.1 | | 781.5 | | 7 | | 55.6 |  |
| 100 | 469 | 117.2 | | 0 | | 137.8 | | 32.2 | | 852.1 | | 840.5 | | 7 | | 54.9 |  |
| 101 | 425 | 106.3 | | 0 | | 153.5 | | 16.5 | | 852.1 | | 887.1 | | 7 | | 49.2 |  |
| 102 | 388.6 | 97.1 | | 0 | | 157.9 | | 12.1 | | 852.1 | | 925.7 | | 7 | | 34.9 |  |
| 103 | 531.3 | 0 | | 0 | | 141.8 | | 28.2 | | 852.1 | | 893.7 | | 7 | | 46.9 |  |
| 104 | 425 | 106.3 | | 0 | | 153.5 | | 16.5 | | 852.1 | | 887.1 | | 7 | | 49.2 |  |
| 105 | 318.8 | 212.5 | | 0 | | 155.7 | | 14.3 | | 852.1 | | 880.4 | | 7 | | 33.4 |  |
| 106 | 401.8 | 94.7 | | 0 | | 147.4 | | 11.4 | | 946.8 | | 852.1 | | 7 | | 54.1 |  |
| 107 | 362.6 | 189 | | 0 | | 164.9 | | 11.6 | | 944.7 | | 755.8 | | 7 | | 55.9 |  |
| 108 | 323.7 | 282.8 | | 0 | | 183.8 | | 10.3 | | 942.7 | | 659.9 | | 7 | | 49.8 |  |
| 109 | 379.5 | 151.2 | | 0 | | 153.9 | | 15.9 | | 1134.3 | | 605 | | 7 | | 47.1 |  |
| 110 | 362.6 | 189 | | 0 | | 164.9 | | 11.6 | | 944.7 | | 755.8 | | 7 | | 55.9 |  |
| 111 | 286.3 | 200.9 | | 0 | | 144.7 | | 11.2 | | 1004.6 | | 803.7 | | 7 | | 38 |  |
| 112 | 362.6 | 189 | | 0 | | 164.9 | | 11.6 | | 944.7 | | 755.8 | | 7 | | 55.9 |  |
| 113 | 439 | 177 | | 0 | | 186 | | 11.1 | | 884.9 | | 707.9 | | 7 | | 56.1 |  |
| 114 | 389.9 | 189 | | 0 | | 145.9 | | 22 | | 944.7 | | 755.8 | | 7 | | 59.09 |  |
| 115 | 362.6 | 189 | | 0 | | 164.9 | | 11.6 | | 944.7 | | 755.8 | | 7 | | 22.9 |  |
| 116 | 337.9 | 189 | | 0 | | 174.9 | | 9.5 | | 944.7 | | 755.8 | | 7 | | 35.1 |  |
| 117 | 374 | 189.2 | | 0 | | 170.1 | | 10.1 | | 926.1 | | 756.7 | | 28 | | 61.09 |  |
| 118 | 313.3 | 262.2 | | 0 | | 175.5 | | 8.6 | | 1046.9 | | 611.8 | | 28 | | 59.8 |  |
| 119 | 425 | 106.3 | | 0 | | 153.5 | | 16.5 | | 852.1 | | 887.1 | | 28 | | 60.29 |  |
| 120 | 425 | 106.3 | | 0 | | 151.4 | | 18.6 | | 936 | | 803.7 | | 28 | | 61.8 |  |
| 121 | 375 | 93.8 | | 0 | | 126.6 | | 23.4 | | 852.1 | | 992.6 | | 28 | | 56.7 |  |
| 122 | 475 | 118.8 | | 0 | | 181.1 | | 8.9 | | 852.1 | | 781.5 | | 28 | | 68.3 |  |
| 123 | 469 | 117.2 | | 0 | | 137.8 | | 32.2 | | 852.1 | | 840.5 | | 28 | | 66.9 |  |
| 124 | 425 | 106.3 | | 0 | | 153.5 | | 16.5 | | 852.1 | | 887.1 | | 28 | | 60.29 |  |
| 125 | 388.6 | 97.1 | | 0 | | 157.9 | | 12.1 | | 852.1 | | 925.7 | | 28 | | 50.7 |  |
| 126 | 531.3 | 0 | | 0 | | 141.8 | | 28.2 | | 852.1 | | 893.7 | | 28 | | 56.4 |  |
| 127 | 425 | 106.3 | | 0 | | 153.5 | | 16.5 | | 852.1 | | 887.1 | | 28 | | 60.29 |  |
| 128 | 318.8 | 212.5 | | 0 | | 155.7 | | 14.3 | | 852.1 | | 880.4 | | 28 | | 55.5 |  |
| 129 | 401.8 | 94.7 | | 0 | | 147.4 | | 11.4 | | 946.8 | | 852.1 | | 28 | | 68.5 |  |
| 130 | 362.6 | 189 | | 0 | | 164.9 | | 11.6 | | 944.7 | | 755.8 | | 28 | | 71.3 |  |
| 131 | 323.7 | 282.8 | | 0 | | 183.8 | | 10.3 | | 942.7 | | 659.9 | | 28 | | 74.7 |  |
| 132 | 379.5 | 151.2 | | 0 | | 153.9 | | 15.9 | | 1134.3 | | 605 | | 28 | | 52.2 |  |
| 133 | 362.6 | 189 | | 0 | | 164.9 | | 11.6 | | 944.7 | | 755.8 | | 28 | | 71.3 |  |
| 134 | 286.3 | 200.9 | | 0 | | 144.7 | | 11.2 | | 1004.6 | | 803.7 | | 28 | | 67.7 |  |
| 135 | 362.6 | 189 | | 0 | | 164.9 | | 11.6 | | 944.7 | | 755.8 | | 28 | | 71.3 |  |
| 136 | 439 | 177 | | 0 | | 186 | | 11.1 | | 884.9 | | 707.9 | | 28 | | 66 |  |
| 137 | 389.9 | 189 | | 0 | | 145.9 | | 22 | | 944.7 | | 755.8 | | 28 | | 74.5 |  |
| 138 | 362.6 | 189 | | 0 | | 164.9 | | 11.6 | | 944.7 | | 755.8 | | 28 | | 71.3 |  |
| 139 | 337.9 | 189 | | 0 | | 174.9 | | 9.5 | | 944.7 | | 755.8 | | 28 | | 49.9 |  |
| 140 | 374 | 189.2 | | 0 | | 170.1 | | 10.1 | | 926.1 | | 756.7 | | 56 | | 63.4 |  |
| 141 | 313.3 | 262.2 | | 0 | | 175.5 | | 8.6 | | 1046.9 | | 611.8 | | 56 | | 64.9 |  |
| 142 | 425 | 106.3 | | 0 | | 153.5 | | 16.5 | | 852.1 | | 887.1 | | 56 | | 64.3 |  |
| 143 | 425 | 106.3 | | 0 | | 151.4 | | 18.6 | | 936 | | 803.7 | | 56 | | 64.9 |  |
| 144 | 375 | 93.8 | | 0 | | 126.6 | | 23.4 | | 852.1 | | 992.6 | | 56 | | 60.2 |  |
| 145 | 475 | 118.8 | | 0 | | 181.1 | | 8.9 | | 852.1 | | 781.5 | | 56 | | 72.3 |  |
| 146 | 469 | 117.2 | | 0 | | 137.8 | | 32.2 | | 852.1 | | 840.5 | | 56 | | 69.3 |  |
| 147 | 425 | 106.3 | | 0 | | 153.5 | | 16.5 | | 852.1 | | 887.1 | | 56 | | 64.3 |  |
| 148 | 388.6 | 97.1 | | 0 | | 157.9 | | 12.1 | | 852.1 | | 925.7 | | 56 | | 55.2 |  |
| 149 | 531.3 | 0 | | 0 | | 141.8 | | 28.2 | | 852.1 | | 893.7 | | 56 | | 58.8 |  |
| 150 | 425 | 106.3 | | 0 | | 153.5 | | 16.5 | | 852.1 | | 887.1 | | 56 | | 64.3 |  |
| 151 | 318.8 | 212.5 | | 0 | | 155.7 | | 14.3 | | 852.1 | | 880.4 | | 56 | | 66.1 |  |
| 152 | 401.8 | 94.7 | | 0 | | 147.4 | | 11.4 | | 946.8 | | 852.1 | | 56 | | 73.7 |  |
| 153 | 362.6 | 189 | | 0 | | 164.9 | | 11.6 | | 944.7 | | 755.8 | | 56 | | 77.3 |  |
| 154 | 323.7 | 282.8 | | 0 | | 183.8 | | 10.3 | | 942.7 | | 659.9 | | 56 | | 80.2 |  |
| 155 | 379.5 | 151.2 | | 0 | | 153.9 | | 15.9 | | 1134.3 | | 605 | | 56 | | 54.9 |  |
| 156 | 362.6 | 189 | | 0 | | 164.9 | | 11.6 | | 944.7 | | 755.8 | | 56 | | 77.3 |  |
| 157 | 286.3 | 200.9 | | 0 | | 144.7 | | 11.2 | | 1004.6 | | 803.7 | | 56 | | 72.99 |  |
| 158 | 362.6 | 189 | | 0 | | 164.9 | | 11.6 | | 944.7 | | 755.8 | | 56 | | 77.3 |  |
| 159 | 439 | 177 | | 0 | | 186 | | 11.1 | | 884.9 | | 707.9 | | 56 | | 71.7 |  |
| 160 | 389.9 | 189 | | 0 | | 145.9 | | 22 | | 944.7 | | 755.8 | | 56 | | 79.4 |  |
| 161 | 362.6 | 189 | | 0 | | 164.9 | | 11.6 | | 944.7 | | 755.8 | | 56 | | 77.3 |  |
| 162 | 337.9 | 189 | | 0 | | 174.9 | | 9.5 | | 944.7 | | 755.8 | | 56 | | 59.89 |  |
| 163 | 374 | 189.2 | | 0 | | 170.1 | | 10.1 | | 926.1 | | 756.7 | | 91 | | 64.9 |  |
| 164 | 313.3 | 262.2 | | 0 | | 175.5 | | 8.6 | | 1046.9 | | 611.8 | | 91 | | 66.6 |  |
| 165 | 425 | 106.3 | | 0 | | 153.5 | | 16.5 | | 852.1 | | 887.1 | | 91 | | 65.2 |  |
| 166 | 425 | 106.3 | | 0 | | 151.4 | | 18.6 | | 936 | | 803.7 | | 91 | | 66.7 |  |
| 167 | 375 | 93.8 | | 0 | | 126.6 | | 23.4 | | 852.1 | | 992.6 | | 91 | | 62.5 |  |
| 168 | 475 | 118.8 | | 0 | | 181.1 | | 8.9 | | 852.1 | | 781.5 | | 91 | | 74.19 |  |
| 169 | 469 | 117.2 | | 0 | | 137.8 | | 32.2 | | 852.1 | | 840.5 | | 91 | | 70.7 |  |
| 170 | 425 | 106.3 | | 0 | | 153.5 | | 16.5 | | 852.1 | | 887.1 | | 91 | | 65.2 |  |
| 171 | 388.6 | 97.1 | | 0 | | 157.9 | | 12.1 | | 852.1 | | 925.7 | | 91 | | 57.6 |  |
| 172 | 531.3 | 0 | | 0 | | 141.8 | | 28.2 | | 852.1 | | 893.7 | | 91 | | 59.2 |  |
| 173 | 425 | 106.3 | | 0 | | 153.5 | | 16.5 | | 852.1 | | 887.1 | | 91 | | 65.2 |  |
| 174 | 318.8 | 212.5 | | 0 | | 155.7 | | 14.3 | | 852.1 | | 880.4 | | 91 | | 68.1 |  |
| 175 | 401.8 | 94.7 | | 0 | | 147.4 | | 11.4 | | 946.8 | | 852.1 | | 91 | | 75.5 |  |
| 176 | 362.6 | 189 | | 0 | | 164.9 | | 11.6 | | 944.7 | | 755.8 | | 91 | | 79.3 |  |
| 177 | 379.5 | 151.2 | | 0 | | 153.9 | | 15.9 | | 1134.3 | | 605 | | 91 | | 56.5 |  |
| 178 | 362.6 | 189 | | 0 | | 164.9 | | 11.6 | | 944.7 | | 755.8 | | 91 | | 79.3 |  |
| 179 | 286.3 | 200.9 | | 0 | | 144.7 | | 11.2 | | 1004.6 | | 803.7 | | 91 | | 76.8 |  |
| 180 | 362.6 | 189 | | 0 | | 164.9 | | 11.6 | | 944.7 | | 755.8 | | 91 | | 79.3 |  |
| 181 | 439 | 177 | | 0 | | 186 | | 11.1 | | 884.9 | | 707.9 | | 91 | | 73.3 |  |
| 182 | 389.9 | 189 | | 0 | | 145.9 | | 22 | | 944.7 | | 755.8 | | 91 | | 82.6 |  |
| 183 | 362.6 | 189 | | 0 | | 164.9 | | 11.6 | | 944.7 | | 755.8 | | 91 | | 79.3 |  |
| 184 | 337.9 | 189 | | 0 | | 174.9 | | 9.5 | | 944.7 | | 755.8 | | 91 | | 67.8 |  |
| 185 | 222.4 | 0 | | 96.7 | | 189.3 | | 4.5 | | 967.1 | | 870.3 | | 3 | | 11.58 |  |
| 186 | 222.4 | 0 | | 96.7 | | 189.3 | | 4.5 | | 967.1 | | 870.3 | | 14 | | 24.45 |  |
| 187 | 222.4 | 0 | | 96.7 | | 189.3 | | 4.5 | | 967.1 | | 870.3 | | 28 | | 24.89 |  |
| 188 | 222.4 | 0 | | 96.7 | | 189.3 | | 4.5 | | 967.1 | | 870.3 | | 56 | | 29.45 |  |
| 189 | 222.4 | 0 | | 96.7 | | 189.3 | | 4.5 | | 967.1 | | 870.3 | | 100 | | 40.71 |  |
| 190 | 233.8 | 0 | | 94.6 | | 197.9 | | 4.6 | | 947 | | 852.2 | | 3 | | 10.38 |  |
| 191 | 233.8 | 0 | | 94.6 | | 197.9 | | 4.6 | | 947 | | 852.2 | | 14 | | 22.14 |  |
| 192 | 233.8 | 0 | | 94.6 | | 197.9 | | 4.6 | | 947 | | 852.2 | | 28 | | 22.84 |  |
| 193 | 233.8 | 0 | | 94.6 | | 197.9 | | 4.6 | | 947 | | 852.2 | | 56 | | 27.66 |  |
| 194 | 233.8 | 0 | | 94.6 | | 197.9 | | 4.6 | | 947 | | 852.2 | | 100 | | 34.56 |  |
| 195 | 194.7 | 0 | | 100.5 | | 165.6 | | 7.5 | | 1006.4 | | 905.9 | | 3 | | 12.45 |  |
| 196 | 194.7 | 0 | | 100.5 | | 165.6 | | 7.5 | | 1006.4 | | 905.9 | | 14 | | 24.99 |  |
| 197 | 194.7 | 0 | | 100.5 | | 165.6 | | 7.5 | | 1006.4 | | 905.9 | | 28 | | 25.72 |  |
| 198 | 194.7 | 0 | | 100.5 | | 165.6 | | 7.5 | | 1006.4 | | 905.9 | | 56 | | 33.96 |  |
| 199 | 194.7 | 0 | | 100.5 | | 165.6 | | 7.5 | | 1006.4 | | 905.9 | | 100 | | 37.34 |  |
| 200 | 190.7 | 0 | | 125.4 | | 162.1 | | 7.8 | | 1090 | | 804 | | 3 | | 15.04 |  |
| 201 | 190.7 | 0 | | 125.4 | | 162.1 | | 7.8 | | 1090 | | 804 | | 14 | | 21.06 |  |
| 202 | 190.7 | 0 | | 125.4 | | 162.1 | | 7.8 | | 1090 | | 804 | | 28 | | 26.4 |  |
| 203 | 190.7 | 0 | | 125.4 | | 162.1 | | 7.8 | | 1090 | | 804 | | 56 | | 35.34 |  |
| 204 | 190.7 | 0 | | 125.4 | | 162.1 | | 7.8 | | 1090 | | 804 | | 100 | | 40.57 |  |
| 205 | 212.1 | 0 | | 121.6 | | 180.3 | | 5.7 | | 1057.6 | | 779.3 | | 3 | | 12.47 |  |
| 206 | 212.1 | 0 | | 121.6 | | 180.3 | | 5.7 | | 1057.6 | | 779.3 | | 14 | | 20.92 |  |
| 207 | 212.1 | 0 | | 121.6 | | 180.3 | | 5.7 | | 1057.6 | | 779.3 | | 28 | | 24.9 |  |
| 208 | 212.1 | 0 | | 121.6 | | 180.3 | | 5.7 | | 1057.6 | | 779.3 | | 56 | | 34.2 |  |
| 209 | 212.1 | 0 | | 121.6 | | 180.3 | | 5.7 | | 1057.6 | | 779.3 | | 100 | | 39.61 |  |
| 210 | 230 | 0 | | 118.3 | | 195.5 | | 4.6 | | 1029.4 | | 758.6 | | 3 | | 10.03 |  |
| 211 | 230 | 0 | | 118.3 | | 195.5 | | 4.6 | | 1029.4 | | 758.6 | | 14 | | 20.08 |  |
| 212 | 230 | 0 | | 118.3 | | 195.5 | | 4.6 | | 1029.4 | | 758.6 | | 28 | | 24.48 |  |
| 213 | 230 | 0 | | 118.3 | | 195.5 | | 4.6 | | 1029.4 | | 758.6 | | 56 | | 31.54 |  |
| 214 | 230 | 0 | | 118.3 | | 195.5 | | 4.6 | | 1029.4 | | 758.6 | | 100 | | 35.34 |  |
| 215 | 190.3 | 0 | | 125.2 | | 161.9 | | 9.9 | | 1088.1 | | 802.6 | | 3 | | 9.45 |  |
| 216 | 190.3 | 0 | | 125.2 | | 161.9 | | 9.9 | | 1088.1 | | 802.6 | | 14 | | 22.72 |  |
| 217 | 190.3 | 0 | | 125.2 | | 161.9 | | 9.9 | | 1088.1 | | 802.6 | | 28 | | 28.47 |  |
| 218 | 190.3 | 0 | | 125.2 | | 161.9 | | 9.9 | | 1088.1 | | 802.6 | | 56 | | 38.56 |  |
| 219 | 190.3 | 0 | | 125.2 | | 161.9 | | 9.9 | | 1088.1 | | 802.6 | | 100 | | 40.39 |  |
| 220 | 166.1 | 0 | | 163.3 | | 176.5 | | 4.5 | | 1058.6 | | 780.1 | | 3 | | 10.76 |  |
| 221 | 166.1 | 0 | | 163.3 | | 176.5 | | 4.5 | | 1058.6 | | 780.1 | | 14 | | 25.48 |  |
| 222 | 166.1 | 0 | | 163.3 | | 176.5 | | 4.5 | | 1058.6 | | 780.1 | | 28 | | 21.54 |  |
| 223 | 166.1 | 0 | | 163.3 | | 176.5 | | 4.5 | | 1058.6 | | 780.1 | | 56 | | 28.63 |  |
| 224 | 166.1 | 0 | | 163.3 | | 176.5 | | 4.5 | | 1058.6 | | 780.1 | | 100 | | 33.54 |  |
| 225 | 168 | 42.1 | | 163.8 | | 121.8 | | 5.7 | | 1058.7 | | 780.1 | | 3 | | 7.75 |  |
| 226 | 168 | 42.1 | | 163.8 | | 121.8 | | 5.7 | | 1058.7 | | 780.1 | | 14 | | 17.82 |  |
| 227 | 168 | 42.1 | | 163.8 | | 121.8 | | 5.7 | | 1058.7 | | 780.1 | | 28 | | 24.24 |  |
| 228 | 168 | 42.1 | | 163.8 | | 121.8 | | 5.7 | | 1058.7 | | 780.1 | | 56 | | 32.85 |  |
| 229 | 168 | 42.1 | | 163.8 | | 121.8 | | 5.7 | | 1058.7 | | 780.1 | | 100 | | 39.23 |  |
| 230 | 213.7 | 98.1 | | 24.5 | | 181.7 | | 6.9 | | 1065.8 | | 785.4 | | 3 | | 18 |  |
| 231 | 213.7 | 98.1 | | 24.5 | | 181.7 | | 6.9 | | 1065.8 | | 785.4 | | 14 | | 30.39 |  |
| 232 | 213.7 | 98.1 | | 24.5 | | 181.7 | | 6.9 | | 1065.8 | | 785.4 | | 28 | | 45.71 |  |
| 233 | 213.7 | 98.1 | | 24.5 | | 181.7 | | 6.9 | | 1065.8 | | 785.4 | | 56 | | 50.77 |  |
| 234 | 213.7 | 98.1 | | 24.5 | | 181.7 | | 6.9 | | 1065.8 | | 785.4 | | 100 | | 53.9 |  |
| 235 | 213.8 | 98.1 | | 24.5 | | 181.7 | | 6.7 | | 1066 | | 785.5 | | 3 | | 13.18 |  |
| 236 | 213.8 | 98.1 | | 24.5 | | 181.7 | | 6.7 | | 1066 | | 785.5 | | 14 | | 17.84 |  |
| 237 | 213.8 | 98.1 | | 24.5 | | 181.7 | | 6.7 | | 1066 | | 785.5 | | 28 | | 40.23 |  |
| 238 | 213.8 | 98.1 | | 24.5 | | 181.7 | | 6.7 | | 1066 | | 785.5 | | 56 | | 47.13 |  |
| 239 | 213.8 | 98.1 | | 24.5 | | 181.7 | | 6.7 | | 1066 | | 785.5 | | 100 | | 49.97 |  |
| 240 | 229.7 | 0 | | 118.2 | | 195.2 | | 6.1 | | 1028.1 | | 757.6 | | 3 | | 13.36 |  |
| 241 | 229.7 | 0 | | 118.2 | | 195.2 | | 6.1 | | 1028.1 | | 757.6 | | 14 | | 22.32 |  |
| 242 | 229.7 | 0 | | 118.2 | | 195.2 | | 6.1 | | 1028.1 | | 757.6 | | 28 | | 24.54 |  |
| 243 | 229.7 | 0 | | 118.2 | | 195.2 | | 6.1 | | 1028.1 | | 757.6 | | 56 | | 31.35 |  |
| 244 | 229.7 | 0 | | 118.2 | | 195.2 | | 6.1 | | 1028.1 | | 757.6 | | 100 | | 40.86 |  |
| 245 | 238.1 | 0 | | 94.1 | | 186.7 | | 7 | | 949.9 | | 847 | | 3 | | 19.93 |  |
| 246 | 238.1 | 0 | | 94.1 | | 186.7 | | 7 | | 949.9 | | 847 | | 14 | | 25.69 |  |
| 247 | 238.1 | 0 | | 94.1 | | 186.7 | | 7 | | 949.9 | | 847 | | 28 | | 30.23 |  |
| 248 | 238.1 | 0 | | 94.1 | | 186.7 | | 7 | | 949.9 | | 847 | | 56 | | 39.59 |  |
| 249 | 238.1 | 0 | | 94.1 | | 186.7 | | 7 | | 949.9 | | 847 | | 100 | | 44.3 |  |
| 250 | 250 | 0 | | 95.7 | | 187.4 | | 5.5 | | 956.9 | | 861.2 | | 3 | | 13.82 |  |
| 251 | 250 | 0 | | 95.7 | | 187.4 | | 5.5 | | 956.9 | | 861.2 | | 14 | | 24.92 |  |
| 252 | 250 | 0 | | 95.7 | | 187.4 | | 5.5 | | 956.9 | | 861.2 | | 28 | | 29.22 |  |
| 253 | 250 | 0 | | 95.7 | | 187.4 | | 5.5 | | 956.9 | | 861.2 | | 56 | | 38.33 |  |
| 254 | 250 | 0 | | 95.7 | | 187.4 | | 5.5 | | 956.9 | | 861.2 | | 100 | | 42.35 |  |
| 255 | 212.5 | 0 | | 100.4 | | 159.3 | | 8.7 | | 1007.8 | | 903.6 | | 3 | | 13.54 |  |
| 256 | 212.5 | 0 | | 100.4 | | 159.3 | | 8.7 | | 1007.8 | | 903.6 | | 14 | | 26.31 |  |
| 257 | 212.5 | 0 | | 100.4 | | 159.3 | | 8.7 | | 1007.8 | | 903.6 | | 28 | | 31.64 |  |
| 258 | 212.5 | 0 | | 100.4 | | 159.3 | | 8.7 | | 1007.8 | | 903.6 | | 56 | | 42.55 |  |
| 259 | 212.5 | 0 | | 100.4 | | 159.3 | | 8.7 | | 1007.8 | | 903.6 | | 100 | | 42.92 |  |
| 260 | 212.6 | 0 | | 100.4 | | 159.4 | | 10.4 | | 1003.8 | | 903.8 | | 3 | | 13.33 |  |
| 261 | 212.6 | 0 | | 100.4 | | 159.4 | | 10.4 | | 1003.8 | | 903.8 | | 14 | | 25.37 |  |
| 262 | 212.6 | 0 | | 100.4 | | 159.4 | | 10.4 | | 1003.8 | | 903.8 | | 28 | | 37.4 |  |
| 263 | 212.6 | 0 | | 100.4 | | 159.4 | | 10.4 | | 1003.8 | | 903.8 | | 56 | | 44.4 |  |
| 264 | 212.6 | 0 | | 100.4 | | 159.4 | | 10.4 | | 1003.8 | | 903.8 | | 100 | | 47.74 |  |
| 265 | 212 | 0 | | 124.8 | | 159 | | 7.8 | | 1085.4 | | 799.5 | | 3 | | 19.52 |  |
| 266 | 212 | 0 | | 124.8 | | 159 | | 7.8 | | 1085.4 | | 799.5 | | 14 | | 31.35 |  |
| 267 | 212 | 0 | | 124.8 | | 159 | | 7.8 | | 1085.4 | | 799.5 | | 28 | | 38.5 |  |
| 268 | 212 | 0 | | 124.8 | | 159 | | 7.8 | | 1085.4 | | 799.5 | | 56 | | 45.08 |  |
| 269 | 212 | 0 | | 124.8 | | 159 | | 7.8 | | 1085.4 | | 799.5 | | 100 | | 47.82 |  |
| 270 | 231.8 | 0 | | 121.6 | | 174 | | 6.7 | | 1056.4 | | 778.5 | | 3 | | 15.44 |  |
| 271 | 231.8 | 0 | | 121.6 | | 174 | | 6.7 | | 1056.4 | | 778.5 | | 14 | | 26.77 |  |
| 272 | 231.8 | 0 | | 121.6 | | 174 | | 6.7 | | 1056.4 | | 778.5 | | 28 | | 33.73 |  |
| 273 | 231.8 | 0 | | 121.6 | | 174 | | 6.7 | | 1056.4 | | 778.5 | | 56 | | 42.7 |  |
| 274 | 231.8 | 0 | | 121.6 | | 174 | | 6.7 | | 1056.4 | | 778.5 | | 100 | | 45.84 |  |
| 275 | 251.4 | 0 | | 118.3 | | 188.5 | | 5.8 | | 1028.4 | | 757.7 | | 3 | | 17.22 |  |
| 276 | 251.4 | 0 | | 118.3 | | 188.5 | | 5.8 | | 1028.4 | | 757.7 | | 14 | | 29.93 |  |
| 277 | 251.4 | 0 | | 118.3 | | 188.5 | | 5.8 | | 1028.4 | | 757.7 | | 28 | | 29.65 |  |
| 278 | 251.4 | 0 | | 118.3 | | 188.5 | | 5.8 | | 1028.4 | | 757.7 | | 56 | | 36.97 |  |
| 279 | 251.4 | 0 | | 118.3 | | 188.5 | | 5.8 | | 1028.4 | | 757.7 | | 100 | | 43.58 |  |
| 280 | 251.4 | 0 | | 118.3 | | 188.5 | | 6.4 | | 1028.4 | | 757.7 | | 3 | | 13.12 |  |
| 281 | 251.4 | 0 | | 118.3 | | 188.5 | | 6.4 | | 1028.4 | | 757.7 | | 14 | | 24.43 |  |
| 282 | 251.4 | 0 | | 118.3 | | 188.5 | | 6.4 | | 1028.4 | | 757.7 | | 28 | | 32.66 |  |
| 283 | 251.4 | 0 | | 118.3 | | 188.5 | | 6.4 | | 1028.4 | | 757.7 | | 56 | | 36.64 |  |
| 284 | 251.4 | 0 | | 118.3 | | 188.5 | | 6.4 | | 1028.4 | | 757.7 | | 100 | | 44.21 |  |
| 285 | 181.4 | 0 | | 167 | | 169.6 | | 7.6 | | 1055.6 | | 777.8 | | 3 | | 13.62 |  |
| 286 | 181.4 | 0 | | 167 | | 169.6 | | 7.6 | | 1055.6 | | 777.8 | | 14 | | 21.6 |  |
| 287 | 181.4 | 0 | | 167 | | 169.6 | | 7.6 | | 1055.6 | | 777.8 | | 28 | | 27.77 |  |
| 288 | 181.4 | 0 | | 167 | | 169.6 | | 7.6 | | 1055.6 | | 777.8 | | 56 | | 35.57 |  |
| 289 | 181.4 | 0 | | 167 | | 169.6 | | 7.6 | | 1055.6 | | 777.8 | | 100 | | 45.37 |  |
| 290 | 182 | 45.2 | | 122 | | 170.2 | | 8.2 | | 1059.4 | | 780.7 | | 3 | | 7.32 |  |
| 291 | 182 | 45.2 | | 122 | | 170.2 | | 8.2 | | 1059.4 | | 780.7 | | 14 | | 21.5 |  |
| 292 | 182 | 45.2 | | 122 | | 170.2 | | 8.2 | | 1059.4 | | 780.7 | | 28 | | 31.27 |  |
| 293 | 182 | 45.2 | | 122 | | 170.2 | | 8.2 | | 1059.4 | | 780.7 | | 56 | | 43.5 |  |
| 294 | 182 | 45.2 | | 122 | | 170.2 | | 8.2 | | 1059.4 | | 780.7 | | 100 | | 48.67 |  |
| 295 | 168.9 | 42.2 | | 124.3 | | 158.3 | | 10.8 | | 1080.8 | | 796.2 | | 3 | | 7.4 |  |
| 296 | 168.9 | 42.2 | | 124.3 | | 158.3 | | 10.8 | | 1080.8 | | 796.2 | | 14 | | 23.51 |  |
| 297 | 168.9 | 42.2 | | 124.3 | | 158.3 | | 10.8 | | 1080.8 | | 796.2 | | 28 | | 31.12 |  |
| 298 | 168.9 | 42.2 | | 124.3 | | 158.3 | | 10.8 | | 1080.8 | | 796.2 | | 56 | | 39.15 |  |
| 299 | 168.9 | 42.2 | | 124.3 | | 158.3 | | 10.8 | | 1080.8 | | 796.2 | | 100 | | 48.15 |  |
| 300 | 290.4 | 0 | | 96.2 | | 168.1 | | 9.4 | | 961.2 | | 865 | | 3 | | 22.5 |  |
| 301 | 290.4 | 0 | | 96.2 | | 168.1 | | 9.4 | | 961.2 | | 865 | | 14 | | 34.67 |  |
| 302 | 290.4 | 0 | | 96.2 | | 168.1 | | 9.4 | | 961.2 | | 865 | | 28 | | 34.74 |  |
| 303 | 290.4 | 0 | | 96.2 | | 168.1 | | 9.4 | | 961.2 | | 865 | | 56 | | 45.08 |  |
| 304 | 290.4 | 0 | | 96.2 | | 168.1 | | 9.4 | | 961.2 | | 865 | | 100 | | 48.97 |  |
| 305 | 277.1 | 0 | | 97.4 | | 160.6 | | 11.8 | | 973.9 | | 875.6 | | 3 | | 23.14 |  |
| 306 | 277.1 | 0 | | 97.4 | | 160.6 | | 11.8 | | 973.9 | | 875.6 | | 14 | | 41.89 |  |
| 307 | 277.1 | 0 | | 97.4 | | 160.6 | | 11.8 | | 973.9 | | 875.6 | | 28 | | 48.28 |  |
| 308 | 277.1 | 0 | | 97.4 | | 160.6 | | 11.8 | | 973.9 | | 875.6 | | 56 | | 51.04 |  |
| 309 | 277.1 | 0 | | 97.4 | | 160.6 | | 11.8 | | 973.9 | | 875.6 | | 100 | | 55.64 |  |
| 310 | 295.7 | 0 | | 95.6 | | 171.5 | | 8.9 | | 955.1 | | 859.2 | | 3 | | 22.95 |  |
| 311 | 295.7 | 0 | | 95.6 | | 171.5 | | 8.9 | | 955.1 | | 859.2 | | 14 | | 35.23 |  |
| 312 | 295.7 | 0 | | 95.6 | | 171.5 | | 8.9 | | 955.1 | | 859.2 | | 28 | | 39.94 |  |
| 313 | 295.7 | 0 | | 95.6 | | 171.5 | | 8.9 | | 955.1 | | 859.2 | | 56 | | 48.72 |  |
| 314 | 295.7 | 0 | | 95.6 | | 171.5 | | 8.9 | | 955.1 | | 859.2 | | 100 | | 52.04 |  |
| 315 | 251.8 | 0 | | 99.9 | | 146.1 | | 12.4 | | 1006 | | 899.8 | | 3 | | 21.02 |  |
| 316 | 251.8 | 0 | | 99.9 | | 146.1 | | 12.4 | | 1006 | | 899.8 | | 14 | | 33.36 |  |
| 317 | 251.8 | 0 | | 99.9 | | 146.1 | | 12.4 | | 1006 | | 899.8 | | 28 | | 33.94 |  |
| 318 | 251.8 | 0 | | 99.9 | | 146.1 | | 12.4 | | 1006 | | 899.8 | | 56 | | 44.14 |  |
| 319 | 251.8 | 0 | | 99.9 | | 146.1 | | 12.4 | | 1006 | | 899.8 | | 100 | | 45.37 |  |
| 320 | 249.1 | 0 | | 98.8 | | 158.1 | | 12.8 | | 987.8 | | 889 | | 3 | | 15.36 |  |
| 321 | 249.1 | 0 | | 98.8 | | 158.1 | | 12.8 | | 987.8 | | 889 | | 14 | | 28.68 |  |
| 322 | 249.1 | 0 | | 98.8 | | 158.1 | | 12.8 | | 987.8 | | 889 | | 28 | | 30.85 |  |
| 323 | 249.1 | 0 | | 98.8 | | 158.1 | | 12.8 | | 987.8 | | 889 | | 56 | | 42.03 |  |
| 324 | 249.1 | 0 | | 98.8 | | 158.1 | | 12.8 | | 987.8 | | 889 | | 100 | | 51.06 |  |
| 325 | 252.3 | 0 | | 98.8 | | 146.3 | | 14.2 | | 987.8 | | 889 | | 3 | | 21.78 |  |
| 326 | 252.3 | 0 | | 98.8 | | 146.3 | | 14.2 | | 987.8 | | 889 | | 14 | | 42.29 |  |
| 327 | 252.3 | 0 | | 98.8 | | 146.3 | | 14.2 | | 987.8 | | 889 | | 28 | | 50.6 |  |
| 328 | 252.3 | 0 | | 98.8 | | 146.3 | | 14.2 | | 987.8 | | 889 | | 56 | | 55.83 |  |
| 329 | 252.3 | 0 | | 98.8 | | 146.3 | | 14.2 | | 987.8 | | 889 | | 100 | | 60.95 |  |
| 330 | 246.8 | 0 | | 125.1 | | 143.3 | | 12 | | 1086.8 | | 800.9 | | 3 | | 23.52 |  |
| 331 | 246.8 | 0 | | 125.1 | | 143.3 | | 12 | | 1086.8 | | 800.9 | | 14 | | 42.22 |  |
| 332 | 246.8 | 0 | | 125.1 | | 143.3 | | 12 | | 1086.8 | | 800.9 | | 28 | | 52.5 |  |
| 333 | 246.8 | 0 | | 125.1 | | 143.3 | | 12 | | 1086.8 | | 800.9 | | 56 | | 60.32 |  |
| 334 | 246.8 | 0 | | 125.1 | | 143.3 | | 12 | | 1086.8 | | 800.9 | | 100 | | 66.42 |  |
| 335 | 275.1 | 0 | | 121.4 | | 159.5 | | 9.9 | | 1053.6 | | 777.5 | | 3 | | 23.8 |  |
| 336 | 275.1 | 0 | | 121.4 | | 159.5 | | 9.9 | | 1053.6 | | 777.5 | | 14 | | 38.77 |  |
| 337 | 275.1 | 0 | | 121.4 | | 159.5 | | 9.9 | | 1053.6 | | 777.5 | | 28 | | 51.33 |  |
| 338 | 275.1 | 0 | | 121.4 | | 159.5 | | 9.9 | | 1053.6 | | 777.5 | | 56 | | 56.85 |  |
| 339 | 275.1 | 0 | | 121.4 | | 159.5 | | 9.9 | | 1053.6 | | 777.5 | | 100 | | 58.61 |  |
| 340 | 297.2 | 0 | | 117.5 | | 174.8 | | 9.5 | | 1022.8 | | 753.5 | | 3 | | 21.91 |  |
| 341 | 297.2 | 0 | | 117.5 | | 174.8 | | 9.5 | | 1022.8 | | 753.5 | | 14 | | 36.99 |  |
| 342 | 297.2 | 0 | | 117.5 | | 174.8 | | 9.5 | | 1022.8 | | 753.5 | | 28 | | 47.4 |  |
| 343 | 297.2 | 0 | | 117.5 | | 174.8 | | 9.5 | | 1022.8 | | 753.5 | | 56 | | 51.96 |  |
| 344 | 297.2 | 0 | | 117.5 | | 174.8 | | 9.5 | | 1022.8 | | 753.5 | | 100 | | 56.74 |  |
| 345 | 213.7 | 0 | | 174.7 | | 154.8 | | 10.2 | | 1053.5 | | 776.4 | | 3 | | 17.57 |  |
| 346 | 213.7 | 0 | | 174.7 | | 154.8 | | 10.2 | | 1053.5 | | 776.4 | | 14 | | 33.73 |  |
| 347 | 213.7 | 0 | | 174.7 | | 154.8 | | 10.2 | | 1053.5 | | 776.4 | | 28 | | 40.15 |  |
| 348 | 213.7 | 0 | | 174.7 | | 154.8 | | 10.2 | | 1053.5 | | 776.4 | | 56 | | 46.64 |  |
| 349 | 213.7 | 0 | | 174.7 | | 154.8 | | 10.2 | | 1053.5 | | 776.4 | | 100 | | 50.08 |  |
| 350 | 213.5 | 0 | | 174.2 | | 154.6 | | 11.7 | | 1052.3 | | 775.5 | | 3 | | 17.37 |  |
| 351 | 213.5 | 0 | | 174.2 | | 154.6 | | 11.7 | | 1052.3 | | 775.5 | | 14 | | 33.7 |  |
| 352 | 213.5 | 0 | | 174.2 | | 154.6 | | 11.7 | | 1052.3 | | 775.5 | | 28 | | 45.94 |  |
| 353 | 213.5 | 0 | | 174.2 | | 154.6 | | 11.7 | | 1052.3 | | 775.5 | | 56 | | 51.43 |  |
| 354 | 213.5 | 0 | | 174.2 | | 154.6 | | 11.7 | | 1052.3 | | 775.5 | | 100 | | 59.3 |  |
| 355 | 277.2 | 97.8 | | 24.5 | | 160.7 | | 11.2 | | 1061.7 | | 782.5 | | 3 | | 30.45 |  |
| 356 | 277.2 | 97.8 | | 24.5 | | 160.7 | | 11.2 | | 1061.7 | | 782.5 | | 14 | | 47.71 |  |
| 357 | 277.2 | 97.8 | | 24.5 | | 160.7 | | 11.2 | | 1061.7 | | 782.5 | | 28 | | 63.14 |  |
| 358 | 277.2 | 97.8 | | 24.5 | | 160.7 | | 11.2 | | 1061.7 | | 782.5 | | 56 | | 66.82 |  |
| 359 | 277.2 | 97.8 | | 24.5 | | 160.7 | | 11.2 | | 1061.7 | | 782.5 | | 100 | | 66.95 |  |
| 360 | 218.2 | 54.6 | | 123.8 | | 140.8 | | 11.9 | | 1075.7 | | 792.7 | | 3 | | 27.42 |  |
| 361 | 218.2 | 54.6 | | 123.8 | | 140.8 | | 11.9 | | 1075.7 | | 792.7 | | 14 | | 35.96 |  |
| 362 | 218.2 | 54.6 | | 123.8 | | 140.8 | | 11.9 | | 1075.7 | | 792.7 | | 28 | | 55.51 |  |
| 363 | 218.2 | 54.6 | | 123.8 | | 140.8 | | 11.9 | | 1075.7 | | 792.7 | | 56 | | 61.99 |  |
| 364 | 218.2 | 54.6 | | 123.8 | | 140.8 | | 11.9 | | 1075.7 | | 792.7 | | 100 | | 63.53 |  |
| 365 | 214.9 | 53.8 | | 121.9 | | 155.6 | | 9.6 | | 1014.3 | | 780.6 | | 3 | | 18.02 |  |
| 366 | 214.9 | 53.8 | | 121.9 | | 155.6 | | 9.6 | | 1014.3 | | 780.6 | | 14 | | 38.6 |  |
| 367 | 214.9 | 53.8 | | 121.9 | | 155.6 | | 9.6 | | 1014.3 | | 780.6 | | 28 | | 52.2 |  |
| 368 | 214.9 | 53.8 | | 121.9 | | 155.6 | | 9.6 | | 1014.3 | | 780.6 | | 56 | | 53.96 |  |
| 369 | 214.9 | 53.8 | | 121.9 | | 155.6 | | 9.6 | | 1014.3 | | 780.6 | | 100 | | 56.63 |  |
| 370 | 218.9 | 0 | | 124.1 | | 158.5 | | 11.3 | | 1078.7 | | 794.9 | | 3 | | 15.34 |  |
| 371 | 218.9 | 0 | | 124.1 | | 158.5 | | 11.3 | | 1078.7 | | 794.9 | | 14 | | 26.05 |  |
| 372 | 218.9 | 0 | | 124.1 | | 158.5 | | 11.3 | | 1078.7 | | 794.9 | | 28 | | 30.22 |  |
| 373 | 218.9 | 0 | | 124.1 | | 158.5 | | 11.3 | | 1078.7 | | 794.9 | | 56 | | 37.27 |  |
| 374 | 218.9 | 0 | | 124.1 | | 158.5 | | 11.3 | | 1078.7 | | 794.9 | | 100 | | 46.23 |  |
| 375 | 376 | 0 | | 0 | | 214.6 | | 0 | | 1003.5 | | 762.4 | | 3 | | 16.28 |  |
| 376 | 376 | 0 | | 0 | | 214.6 | | 0 | | 1003.5 | | 762.4 | | 14 | | 25.62 |  |
| 377 | 376 | 0 | | 0 | | 214.6 | | 0 | | 1003.5 | | 762.4 | | 28 | | 31.97 |  |
| 378 | 376 | 0 | | 0 | | 214.6 | | 0 | | 1003.5 | | 762.4 | | 56 | | 36.3 |  |
| 379 | 376 | 0 | | 0 | | 214.6 | | 0 | | 1003.5 | | 762.4 | | 100 | | 43.06 |  |
| 380 | 500 | 0 | | 0 | | 140 | | 4 | | 966 | | 853 | | 28 | | 67.57 |  |
| 381 | 475 | 0 | | 59 | | 142 | | 1.9 | | 1098 | | 641 | | 28 | | 57.23 |  |
| 382 | 315 | 137 | | 0 | | 145 | | 5.9 | | 1130 | | 745 | | 28 | | 81.75 |  |
| 383 | 505 | 0 | | 60 | | 195 | | 0 | | 1030 | | 630 | | 28 | | 64.02 |  |
| 384 | 451 | 0 | | 0 | | 165 | | 11.3 | | 1030 | | 745 | | 28 | | 78.8 |  |
| 385 | 516 | 0 | | 0 | | 162 | | 8.2 | | 801 | | 802 | | 28 | | 41.37 |  |
| 386 | 520 | 0 | | 0 | | 170 | | 5.2 | | 855 | | 855 | | 28 | | 60.28 |  |
| 387 | 528 | 0 | | 0 | | 185 | | 6.9 | | 920 | | 720 | | 28 | | 56.83 |  |
| 388 | 520 | 0 | | 0 | | 175 | | 5.2 | | 870 | | 805 | | 28 | | 51.02 |  |
| 389 | 385 | 0 | | 136 | | 158 | | 20 | | 903 | | 768 | | 28 | | 55.55 |  |
| 390 | 500.1 | 0 | | 0 | | 200 | | 3 | | 1124.4 | | 613.2 | | 28 | | 44.13 |  |
| 391 | 450.1 | 50 | | 0 | | 200 | | 3 | | 1124.4 | | 613.2 | | 28 | | 39.38 |  |
| 392 | 397 | 17.2 | | 158 | | 167 | | 20.8 | | 967 | | 633 | | 28 | | 55.65 |  |
| 393 | 333 | 17.5 | | 163 | | 167 | | 17.9 | | 996 | | 652 | | 28 | | 47.28 |  |
| 394 | 334 | 17.6 | | 158 | | 189 | | 15.3 | | 967 | | 633 | | 28 | | 44.33 |  |
| 395 | 405 | 0 | | 0 | | 175 | | 0 | | 1120 | | 695 | | 28 | | 52.3 |  |
| 396 | 200 | 200 | | 0 | | 190 | | 0 | | 1145 | | 660 | | 28 | | 49.25 |  |
| 397 | 516 | 0 | | 0 | | 162 | | 8.3 | | 801 | | 802 | | 28 | | 41.37 |  |
| 398 | 145 | 116 | | 119 | | 184 | | 5.7 | | 833 | | 880 | | 28 | | 29.16 |  |
| 399 | 160 | 128 | | 122 | | 182 | | 6.4 | | 824 | | 879 | | 28 | | 39.4 |  |
| 400 | 234 | 156 | | 0 | | 189 | | 5.9 | | 981 | | 760 | | 28 | | 39.3 |  |
| 401 | 250 | 180 | | 95 | | 159 | | 9.5 | | 860 | | 800 | | 28 | | 67.87 |  |
| 402 | 475 | 0 | | 0 | | 162 | | 9.5 | | 1044 | | 662 | | 28 | | 58.52 |  |
| 403 | 285 | 190 | | 0 | | 163 | | 7.6 | | 1031 | | 685 | | 28 | | 53.58 |  |
| 404 | 356 | 119 | | 0 | | 160 | | 9 | | 1061 | | 657 | | 28 | | 59 |  |
| 405 | 275 | 180 | | 120 | | 162 | | 10.4 | | 830 | | 765 | | 28 | | 76.24 |  |
| 406 | 500 | 0 | | 0 | | 151 | | 9 | | 1033 | | 655 | | 28 | | 69.84 |  |
| 407 | 165 | 0 | | 143.6 | | 163.8 | | 0 | | 1005.6 | | 900.9 | | 3 | | 14.4 |  |
| 408 | 165 | 128.5 | | 132.1 | | 175.1 | | 8.1 | | 1005.8 | | 746.6 | | 3 | | 19.42 |  |
| 409 | 178 | 129.8 | | 118.6 | | 179.9 | | 3.6 | | 1007.3 | | 746.8 | | 3 | | 20.73 |  |
| 410 | 167.4 | 129.9 | | 128.6 | | 175.5 | | 7.8 | | 1006.3 | | 746.6 | | 3 | | 14.94 |  |
| 411 | 172.4 | 13.6 | | 172.4 | | 156.8 | | 4.1 | | 1006.3 | | 856.4 | | 3 | | 21.29 |  |
| 412 | 173.5 | 50.1 | | 173.5 | | 164.8 | | 6.5 | | 1006.2 | | 793.5 | | 3 | | 23.08 |  |
| 413 | 167 | 75.4 | | 167 | | 164 | | 7.9 | | 1007.3 | | 770.1 | | 3 | | 15.52 |  |
| 414 | 173.8 | 93.4 | | 159.9 | | 172.3 | | 9.7 | | 1007.2 | | 746.6 | | 3 | | 15.82 |  |
| 415 | 190.3 | 0 | | 125.2 | | 166.6 | | 9.9 | | 1079 | | 798.9 | | 3 | | 12.55 |  |
| 416 | 250 | 0 | | 95.7 | | 191.8 | | 5.3 | | 948.9 | | 857.2 | | 3 | | 8.49 |  |
| 417 | 213.5 | 0 | | 174.2 | | 159.2 | | 11.7 | | 1043.6 | | 771.9 | | 3 | | 15.61 |  |
| 418 | 194.7 | 0 | | 100.5 | | 170.2 | | 7.5 | | 998 | | 901.8 | | 3 | | 12.18 |  |
| 419 | 251.4 | 0 | | 118.3 | | 192.9 | | 5.8 | | 1043.6 | | 754.3 | | 3 | | 11.98 |  |
| 420 | 165 | 0 | | 143.6 | | 163.8 | | 0 | | 1005.6 | | 900.9 | | 14 | | 16.88 |  |
| 421 | 165 | 128.5 | | 132.1 | | 175.1 | | 8.1 | | 1005.8 | | 746.6 | | 14 | | 33.09 |  |
| 422 | 178 | 129.8 | | 118.6 | | 179.9 | | 3.6 | | 1007.3 | | 746.8 | | 14 | | 34.24 |  |
| 423 | 167.4 | 129.9 | | 128.6 | | 175.5 | | 7.8 | | 1006.3 | | 746.6 | | 14 | | 31.81 |  |
| 424 | 172.4 | 13.6 | | 172.4 | | 156.8 | | 4.1 | | 1006.3 | | 856.4 | | 14 | | 29.75 |  |
| 425 | 173.5 | 50.1 | | 173.5 | | 164.8 | | 6.5 | | 1006.2 | | 793.5 | | 14 | | 33.01 |  |
| 426 | 167 | 75.4 | | 167 | | 164 | | 7.9 | | 1007.3 | | 770.1 | | 14 | | 32.9 |  |
| 427 | 173.8 | 93.4 | | 159.9 | | 172.3 | | 9.7 | | 1007.2 | | 746.6 | | 14 | | 29.55 |  |
| 428 | 190.3 | 0 | | 125.2 | | 166.6 | | 9.9 | | 1079 | | 798.9 | | 14 | | 19.42 |  |
| 429 | 250 | 0 | | 95.7 | | 191.8 | | 5.3 | | 948.9 | | 857.2 | | 14 | | 24.66 |  |
| 430 | 213.5 | 0 | | 174.2 | | 159.2 | | 11.7 | | 1043.6 | | 771.9 | | 14 | | 29.59 |  |
| 431 | 194.7 | 0 | | 100.5 | | 170.2 | | 7.5 | | 998 | | 901.8 | | 14 | | 24.28 |  |
| 432 | 251.4 | 0 | | 118.3 | | 192.9 | | 5.8 | | 1043.6 | | 754.3 | | 14 | | 20.73 |  |
| 433 | 165 | 0 | | 143.6 | | 163.8 | | 0 | | 1005.6 | | 900.9 | | 28 | | 26.2 |  |
| 434 | 165 | 128.5 | | 132.1 | | 175.1 | | 8.1 | | 1005.8 | | 746.6 | | 28 | | 46.39 |  |
| 435 | 178 | 129.8 | | 118.6 | | 179.9 | | 3.6 | | 1007.3 | | 746.8 | | 28 | | 39.16 |  |
| 436 | 167.4 | 129.9 | | 128.6 | | 175.5 | | 7.8 | | 1006.3 | | 746.6 | | 28 | | 41.2 |  |
| 437 | 172.4 | 13.6 | | 172.4 | | 156.8 | | 4.1 | | 1006.3 | | 856.4 | | 28 | | 33.69 |  |
| 438 | 173.5 | 50.1 | | 173.5 | | 164.8 | | 6.5 | | 1006.2 | | 793.5 | | 28 | | 38.2 |  |
| 439 | 167 | 75.4 | | 167 | | 164 | | 7.9 | | 1007.3 | | 770.1 | | 28 | | 41.41 |  |
| 440 | 173.8 | 93.4 | | 159.9 | | 172.3 | | 9.7 | | 1007.2 | | 746.6 | | 28 | | 37.81 |  |
| 441 | 190.3 | 0 | | 125.2 | | 166.6 | | 9.9 | | 1079 | | 798.9 | | 28 | | 24.85 |  |
| 442 | 250 | 0 | | 95.7 | | 191.8 | | 5.3 | | 948.9 | | 857.2 | | 28 | | 27.22 |  |
| 443 | 213.5 | 0 | | 174.2 | | 159.2 | | 11.7 | | 1043.6 | | 771.9 | | 28 | | 44.64 |  |
| 444 | 194.7 | 0 | | 100.5 | | 170.2 | | 7.5 | | 998 | | 901.8 | | 28 | | 37.27 |  |
| 445 | 251.4 | 0 | | 118.3 | | 192.9 | | 5.8 | | 1043.6 | | 754.3 | | 28 | | 33.27 |  |
| 446 | 165 | 0 | | 143.6 | | 163.8 | | 0 | | 1005.6 | | 900.9 | | 56 | | 36.56 |  |
| 447 | 165 | 128.5 | | 132.1 | | 175.1 | | 8.1 | | 1005.8 | | 746.6 | | 56 | | 53.72 |  |
| 448 | 178 | 129.8 | | 118.6 | | 179.9 | | 3.6 | | 1007.3 | | 746.8 | | 56 | | 48.59 |  |
| 449 | 167.4 | 129.9 | | 128.6 | | 175.5 | | 7.8 | | 1006.3 | | 746.6 | | 56 | | 51.72 |  |
| 450 | 172.4 | 13.6 | | 172.4 | | 156.8 | | 4.1 | | 1006.3 | | 856.4 | | 56 | | 35.85 |  |
| 451 | 173.5 | 50.1 | | 173.5 | | 164.8 | | 6.5 | | 1006.2 | | 793.5 | | 56 | | 53.77 |  |
| 452 | 167 | 75.4 | | 167 | | 164 | | 7.9 | | 1007.3 | | 770.1 | | 56 | | 53.46 |  |
| 453 | 173.8 | 93.4 | | 159.9 | | 172.3 | | 9.7 | | 1007.2 | | 746.6 | | 56 | | 48.99 |  |
| 454 | 190.3 | 0 | | 125.2 | | 166.6 | | 9.9 | | 1079 | | 798.9 | | 56 | | 31.72 |  |
| 455 | 250 | 0 | | 95.7 | | 191.8 | | 5.3 | | 948.9 | | 857.2 | | 56 | | 39.64 |  |
| 456 | 213.5 | 0 | | 174.2 | | 159.2 | | 11.7 | | 1043.6 | | 771.9 | | 56 | | 51.26 |  |
| 457 | 194.7 | 0 | | 100.5 | | 170.2 | | 7.5 | | 998 | | 901.8 | | 56 | | 43.39 |  |
| 458 | 251.4 | 0 | | 118.3 | | 192.9 | | 5.8 | | 1043.6 | | 754.3 | | 56 | | 39.27 |  |
| 459 | 165 | 0 | | 143.6 | | 163.8 | | 0 | | 1005.6 | | 900.9 | | 100 | | 37.96 |  |
| 460 | 165 | 128.5 | | 132.1 | | 175.1 | | 8.1 | | 1005.8 | | 746.6 | | 100 | | 55.02 |  |
| 461 | 178 | 129.8 | | 118.6 | | 179.9 | | 3.6 | | 1007.3 | | 746.8 | | 100 | | 49.99 |  |
| 462 | 167.4 | 129.9 | | 128.6 | | 175.5 | | 7.8 | | 1006.3 | | 746.6 | | 100 | | 53.66 |  |
| 463 | 172.4 | 13.6 | | 172.4 | | 156.8 | | 4.1 | | 1006.3 | | 856.4 | | 100 | | 37.68 |  |
| 464 | 173.5 | 50.1 | | 173.5 | | 164.8 | | 6.5 | | 1006.2 | | 793.5 | | 100 | | 56.06 |  |
| 465 | 167 | 75.4 | | 167 | | 164 | | 7.9 | | 1007.3 | | 770.1 | | 100 | | 56.81 |  |
| 466 | 173.8 | 93.4 | | 159.9 | | 172.3 | | 9.7 | | 1007.2 | | 746.6 | | 100 | | 50.94 |  |
| 467 | 190.3 | 0 | | 125.2 | | 166.6 | | 9.9 | | 1079 | | 798.9 | | 100 | | 33.56 |  |
| 468 | 250 | 0 | | 95.7 | | 191.8 | | 5.3 | | 948.9 | | 857.2 | | 100 | | 41.16 |  |
| 469 | 213.5 | 0 | | 174.2 | | 159.2 | | 11.7 | | 1043.6 | | 771.9 | | 100 | | 52.96 |  |
| 470 | 194.7 | 0 | | 100.5 | | 170.2 | | 7.5 | | 998 | | 901.8 | | 100 | | 44.28 |  |
| 471 | 251.4 | 0 | | 118.3 | | 192.9 | | 5.8 | | 1043.6 | | 754.3 | | 100 | | 40.15 |  |
| 472 | 446 | 24 | | 79 | | 162 | | 11.6 | | 967 | | 712 | | 28 | | 57.03 |  |
| 473 | 446 | 24 | | 79 | | 162 | | 11.6 | | 967 | | 712 | | 28 | | 44.42 |  |
| 474 | 446 | 24 | | 79 | | 162 | | 11.6 | | 967 | | 712 | | 28 | | 51.02 |  |
| 475 | 446 | 24 | | 79 | | 162 | | 10.3 | | 967 | | 712 | | 28 | | 53.39 |  |
| 476 | 446 | 24 | | 79 | | 162 | | 11.6 | | 967 | | 712 | | 3 | | 35.36 |  |
| 477 | 446 | 24 | | 79 | | 162 | | 11.6 | | 967 | | 712 | | 3 | | 25.02 |  |
| 478 | 446 | 24 | | 79 | | 162 | | 11.6 | | 967 | | 712 | | 3 | | 23.35 |  |
| 479 | 446 | 24 | | 79 | | 162 | | 11.6 | | 967 | | 712 | | 7 | | 52.01 |  |
| 480 | 446 | 24 | | 79 | | 162 | | 11.6 | | 967 | | 712 | | 7 | | 38.02 |  |
| 481 | 446 | 24 | | 79 | | 162 | | 11.6 | | 967 | | 712 | | 7 | | 39.3 |  |
| 482 | 446 | 24 | | 79 | | 162 | | 11.6 | | 967 | | 712 | | 56 | | 61.07 |  |
| 483 | 446 | 24 | | 79 | | 162 | | 11.6 | | 967 | | 712 | | 56 | | 56.14 |  |
| 484 | 446 | 24 | | 79 | | 162 | | 11.6 | | 967 | | 712 | | 56 | | 55.25 |  |
| 485 | 446 | 24 | | 79 | | 162 | | 10.3 | | 967 | | 712 | | 56 | | 54.77 |  |
| 486 | 387 | 20 | | 94 | | 157 | | 14.3 | | 938 | | 845 | | 28 | | 50.24 |  |
| 487 | 387 | 20 | | 94 | | 157 | | 13.9 | | 938 | | 845 | | 28 | | 46.68 |  |
| 488 | 387 | 20 | | 94 | | 157 | | 11.6 | | 938 | | 845 | | 28 | | 46.68 |  |
| 489 | 387 | 20 | | 94 | | 157 | | 14.3 | | 938 | | 845 | | 3 | | 22.75 |  |
| 490 | 387 | 20 | | 94 | | 157 | | 13.9 | | 938 | | 845 | | 3 | | 25.51 |  |
| 491 | 387 | 20 | | 94 | | 157 | | 11.6 | | 938 | | 845 | | 3 | | 34.77 |  |
| 492 | 387 | 20 | | 94 | | 157 | | 14.3 | | 938 | | 845 | | 7 | | 36.84 |  |
| 493 | 387 | 20 | | 94 | | 157 | | 13.9 | | 938 | | 845 | | 7 | | 45.9 |  |
| 494 | 387 | 20 | | 94 | | 157 | | 11.6 | | 938 | | 845 | | 7 | | 41.67 |  |
| 495 | 387 | 20 | | 94 | | 157 | | 14.3 | | 938 | | 845 | | 56 | | 56.34 |  |
| 496 | 387 | 20 | | 94 | | 157 | | 13.9 | | 938 | | 845 | | 56 | | 47.97 |  |
| 497 | 387 | 20 | | 94 | | 157 | | 11.6 | | 938 | | 845 | | 56 | | 61.46 |  |
| 498 | 355 | 19 | | 97 | | 145 | | 13.1 | | 967 | | 871 | | 28 | | 44.03 |  |
| 499 | 355 | 19 | | 97 | | 145 | | 12.3 | | 967 | | 871 | | 28 | | 55.45 |  |
| 500 | 491 | 26 | | 123 | | 210 | | 3.9 | | 882 | | 699 | | 28 | | 55.55 |  |
| 501 | 491 | 26 | | 123 | | 201 | | 3.9 | | 822 | | 699 | | 28 | | 57.92 |  |
| 502 | 491 | 26 | | 123 | | 210 | | 3.9 | | 882 | | 699 | | 3 | | 25.61 |  |
| 503 | 491 | 26 | | 123 | | 210 | | 3.9 | | 882 | | 699 | | 7 | | 33.49 |  |
| 504 | 491 | 26 | | 123 | | 210 | | 3.9 | | 882 | | 699 | | 56 | | 59.59 |  |
| 505 | 491 | 26 | | 123 | | 201 | | 3.9 | | 822 | | 699 | | 3 | | 29.55 |  |
| 506 | 491 | 26 | | 123 | | 201 | | 3.9 | | 822 | | 699 | | 7 | | 37.92 |  |
| 507 | 491 | 26 | | 123 | | 201 | | 3.9 | | 822 | | 699 | | 56 | | 61.86 |  |
| 508 | 424 | 22 | | 132 | | 178 | | 8.5 | | 822 | | 750 | | 28 | | 62.05 |  |
| 509 | 424 | 22 | | 132 | | 178 | | 8.5 | | 882 | | 750 | | 3 | | 32.01 |  |
| 510 | 424 | 22 | | 132 | | 168 | | 8.9 | | 822 | | 750 | | 28 | | 72.1 |  |
| 511 | 424 | 22 | | 132 | | 178 | | 8.5 | | 822 | | 750 | | 7 | | 39 |  |
| 512 | 424 | 22 | | 132 | | 178 | | 8.5 | | 822 | | 750 | | 56 | | 65.7 |  |
| 513 | 424 | 22 | | 132 | | 168 | | 8.9 | | 822 | | 750 | | 3 | | 32.11 |  |
| 514 | 424 | 22 | | 132 | | 168 | | 8.9 | | 822 | | 750 | | 7 | | 40.29 |  |
| 515 | 424 | 22 | | 132 | | 168 | | 8.9 | | 822 | | 750 | | 56 | | 74.36 |  |
| 516 | 202 | 11 | | 141 | | 206 | | 1.7 | | 942 | | 801 | | 28 | | 21.97 |  |
| 517 | 202 | 11 | | 141 | | 206 | | 1.7 | | 942 | | 801 | | 3 | | 9.85 |  |
| 518 | 202 | 11 | | 141 | | 206 | | 1.7 | | 942 | | 801 | | 7 | | 15.07 |  |
| 519 | 202 | 11 | | 141 | | 206 | | 1.7 | | 942 | | 801 | | 56 | | 23.25 |  |
| 520 | 284 | 15 | | 141 | | 179 | | 5.5 | | 842 | | 801 | | 28 | | 43.73 |  |
| 521 | 284 | 15 | | 141 | | 179 | | 5.5 | | 842 | | 801 | | 3 | | 13.4 |  |
| 522 | 284 | 15 | | 141 | | 179 | | 5.5 | | 842 | | 801 | | 7 | | 24.13 |  |
| 523 | 284 | 15 | | 141 | | 179 | | 5.5 | | 842 | | 801 | | 56 | | 44.52 |  |
| 524 | 359 | 19 | | 141 | | 154 | | 10.9 | | 942 | | 801 | | 28 | | 62.94 |  |
| 525 | 359 | 19 | | 141 | | 154 | | 10.9 | | 942 | | 801 | | 28 | | 59.49 |  |
| 526 | 359 | 19 | | 141 | | 154 | | 10.9 | | 942 | | 801 | | 3 | | 25.12 |  |
| 527 | 359 | 19 | | 141 | | 154 | | 10.9 | | 942 | | 801 | | 3 | | 23.64 |  |
| 528 | 359 | 19 | | 141 | | 154 | | 10.9 | | 942 | | 801 | | 7 | | 35.75 |  |
| 529 | 359 | 19 | | 141 | | 154 | | 10.9 | | 942 | | 801 | | 7 | | 38.61 |  |
| 530 | 359 | 19 | | 141 | | 154 | | 10.9 | | 942 | | 801 | | 56 | | 68.75 |  |
| 531 | 359 | 19 | | 141 | | 154 | | 10.9 | | 942 | | 801 | | 56 | | 66.78 |  |
| 532 | 436 | 0 | | 0 | | 218 | | 0 | | 838.4 | | 719.7 | | 28 | | 23.85 |  |
| 533 | 289 | 0 | | 0 | | 192 | | 0 | | 913.2 | | 895.3 | | 90 | | 32.07 |  |
| 534 | 289 | 0 | | 0 | | 192 | | 0 | | 913.2 | | 895.3 | | 3 | | 11.65 |  |
| 535 | 393 | 0 | | 0 | | 192 | | 0 | | 940.6 | | 785.6 | | 3 | | 19.2 |  |
| 536 | 393 | 0 | | 0 | | 192 | | 0 | | 940.6 | | 785.6 | | 90 | | 48.85 |  |
| 537 | 393 | 0 | | 0 | | 192 | | 0 | | 940.6 | | 785.6 | | 28 | | 39.6 |  |
| 538 | 480 | 0 | | 0 | | 192 | | 0 | | 936.2 | | 712.2 | | 28 | | 43.94 |  |
| 539 | 480 | 0 | | 0 | | 192 | | 0 | | 936.2 | | 712.2 | | 7 | | 34.57 |  |
| 540 | 480 | 0 | | 0 | | 192 | | 0 | | 936.2 | | 712.2 | | 90 | | 54.32 |  |
| 541 | 480 | 0 | | 0 | | 192 | | 0 | | 936.2 | | 712.2 | | 3 | | 24.4 |  |
| 542 | 333 | 0 | | 0 | | 192 | | 0 | | 931.2 | | 842.6 | | 3 | | 15.62 |  |
| 543 | 255 | 0 | | 0 | | 192 | | 0 | | 889.8 | | 945 | | 90 | | 21.86 |  |
| 544 | 255 | 0 | | 0 | | 192 | | 0 | | 889.8 | | 945 | | 7 | | 10.22 |  |
| 545 | 289 | 0 | | 0 | | 192 | | 0 | | 913.2 | | 895.3 | | 7 | | 14.6 |  |
| 546 | 255 | 0 | | 0 | | 192 | | 0 | | 889.8 | | 945 | | 28 | | 18.75 |  |
| 547 | 333 | 0 | | 0 | | 192 | | 0 | | 931.2 | | 842.6 | | 28 | | 31.97 |  |
| 548 | 333 | 0 | | 0 | | 192 | | 0 | | 931.2 | | 842.6 | | 7 | | 23.4 |  |
| 549 | 289 | 0 | | 0 | | 192 | | 0 | | 913.2 | | 895.3 | | 28 | | 25.57 |  |
| 550 | 333 | 0 | | 0 | | 192 | | 0 | | 931.2 | | 842.6 | | 90 | | 41.68 |  |
| 551 | 393 | 0 | | 0 | | 192 | | 0 | | 940.6 | | 785.6 | | 7 | | 27.74 |  |
| 552 | 255 | 0 | | 0 | | 192 | | 0 | | 889.8 | | 945 | | 3 | | 8.2 |  |
| 553 | 158.8 | 238.2 | | 0 | | 185.7 | | 0 | | 1040.6 | | 734.3 | | 7 | | 9.62 |  |
| 554 | 239.6 | 359.4 | | 0 | | 185.7 | | 0 | | 941.6 | | 664.3 | | 7 | | 25.42 |  |
| 555 | 238.2 | 158.8 | | 0 | | 185.7 | | 0 | | 1040.6 | | 734.3 | | 7 | | 15.69 |  |
| 556 | 181.9 | 272.8 | | 0 | | 185.7 | | 0 | | 1012.4 | | 714.3 | | 28 | | 27.94 |  |
| 557 | 193.5 | 290.2 | | 0 | | 185.7 | | 0 | | 998.2 | | 704.3 | | 28 | | 32.63 |  |
| 558 | 255.5 | 170.3 | | 0 | | 185.7 | | 0 | | 1026.6 | | 724.3 | | 7 | | 17.24 |  |
| 559 | 272.8 | 181.9 | | 0 | | 185.7 | | 0 | | 1012.4 | | 714.3 | | 7 | | 19.77 |  |
| 560 | 239.6 | 359.4 | | 0 | | 185.7 | | 0 | | 941.6 | | 664.3 | | 28 | | 39.44 |  |
| 561 | 220.8 | 147.2 | | 0 | | 185.7 | | 0 | | 1055 | | 744.3 | | 28 | | 25.75 |  |
| 562 | 397 | 0 | | 0 | | 185.7 | | 0 | | 1040.6 | | 734.3 | | 28 | | 33.08 |  |
| 563 | 382.5 | 0 | | 0 | | 185.7 | | 0 | | 1047.8 | | 739.3 | | 7 | | 24.07 |  |
| 564 | 210.7 | 316.1 | | 0 | | 185.7 | | 0 | | 977 | | 689.3 | | 7 | | 21.82 |  |
| 565 | 158.8 | 238.2 | | 0 | | 185.7 | | 0 | | 1040.6 | | 734.3 | | 28 | | 21.07 |  |
| 566 | 295.8 | 0 | | 0 | | 185.7 | | 0 | | 1091.4 | | 769.3 | | 7 | | 14.84 |  |
| 567 | 255.5 | 170.3 | | 0 | | 185.7 | | 0 | | 1026.6 | | 724.3 | | 28 | | 32.05 |  |
| 568 | 203.5 | 135.7 | | 0 | | 185.7 | | 0 | | 1076.2 | | 759.3 | | 7 | | 11.96 |  |
| 569 | 397 | 0 | | 0 | | 185.7 | | 0 | | 1040.6 | | 734.3 | | 7 | | 25.45 |  |
| 570 | 381.4 | 0 | | 0 | | 185.7 | | 0 | | 1104.6 | | 784.3 | | 28 | | 22.49 |  |
| 571 | 295.8 | 0 | | 0 | | 185.7 | | 0 | | 1091.4 | | 769.3 | | 28 | | 25.22 |  |
| 572 | 228 | 342.1 | | 0 | | 185.7 | | 0 | | 955.8 | | 674.3 | | 28 | | 39.7 |  |
| 573 | 220.8 | 147.2 | | 0 | | 185.7 | | 0 | | 1055 | | 744.3 | | 7 | | 13.09 |  |
| 574 | 316.1 | 210.7 | | 0 | | 185.7 | | 0 | | 977 | | 689.3 | | 28 | | 38.7 |  |
| 575 | 135.7 | 203.5 | | 0 | | 185.7 | | 0 | | 1076.2 | | 759.3 | | 7 | | 7.51 |  |
| 576 | 238.1 | 0 | | 0 | | 185.7 | | 0 | | 1118.8 | | 789.3 | | 28 | | 17.58 |  |
| 577 | 339.2 | 0 | | 0 | | 185.7 | | 0 | | 1069.2 | | 754.3 | | 7 | | 21.18 |  |
| 578 | 135.7 | 203.5 | | 0 | | 185.7 | | 0 | | 1076.2 | | 759.3 | | 28 | | 18.2 |  |
| 579 | 193.5 | 290.2 | | 0 | | 185.7 | | 0 | | 998.2 | | 704.3 | | 7 | | 17.2 |  |
| 580 | 203.5 | 135.7 | | 0 | | 185.7 | | 0 | | 1076.2 | | 759.3 | | 28 | | 22.63 |  |
| 581 | 290.2 | 193.5 | | 0 | | 185.7 | | 0 | | 998.2 | | 704.3 | | 7 | | 21.86 |  |
| 582 | 181.9 | 272.8 | | 0 | | 185.7 | | 0 | | 1012.4 | | 714.3 | | 7 | | 12.37 |  |
| 583 | 170.3 | 155.5 | | 0 | | 185.7 | | 0 | | 1026.6 | | 724.3 | | 28 | | 25.73 |  |
| 584 | 210.7 | 316.1 | | 0 | | 185.7 | | 0 | | 977 | | 689.3 | | 28 | | 37.81 |  |
| 585 | 228 | 342.1 | | 0 | | 185.7 | | 0 | | 955.8 | | 674.3 | | 7 | | 21.92 |  |
| 586 | 290.2 | 193.5 | | 0 | | 185.7 | | 0 | | 998.2 | | 704.3 | | 28 | | 33.04 |  |
| 587 | 381.4 | 0 | | 0 | | 185.7 | | 0 | | 1104.6 | | 784.3 | | 7 | | 14.54 |  |
| 588 | 238.2 | 158.8 | | 0 | | 185.7 | | 0 | | 1040.6 | | 734.3 | | 28 | | 26.91 |  |
| 589 | 186.2 | 124.1 | | 0 | | 185.7 | | 0 | | 1083.4 | | 764.3 | | 7 | | 8 |  |
| 590 | 339.2 | 0 | | 0 | | 185.7 | | 0 | | 1069.2 | | 754.3 | | 28 | | 31.9 |  |
| 591 | 238.1 | 0 | | 0 | | 185.7 | | 0 | | 1118.8 | | 789.3 | | 7 | | 10.34 |  |
| 592 | 252.5 | 0 | | 0 | | 185.7 | | 0 | | 1111.6 | | 784.3 | | 28 | | 19.77 |  |
| 593 | 382.5 | 0 | | 0 | | 185.7 | | 0 | | 1047.8 | | 739.3 | | 28 | | 37.44 |  |
| 594 | 252.5 | 0 | | 0 | | 185.7 | | 0 | | 1111.6 | | 784.3 | | 7 | | 11.48 |  |
| 595 | 316.1 | 210.7 | | 0 | | 185.7 | | 0 | | 977 | | 689.3 | | 7 | | 24.44 |  |
| 596 | 186.2 | 124.1 | | 0 | | 185.7 | | 0 | | 1083.4 | | 764.3 | | 28 | | 17.6 |  |
| 597 | 170.3 | 155.5 | | 0 | | 185.7 | | 0 | | 1026.6 | | 724.3 | | 7 | | 10.73 |  |
| 598 | 272.8 | 181.9 | | 0 | | 185.7 | | 0 | | 1012.4 | | 714.3 | | 28 | | 31.38 |  |
| 599 | 339 | 0 | | 0 | | 197 | | 0 | | 968 | | 781 | | 3 | | 13.22 |  |
| 600 | 339 | 0 | | 0 | | 197 | | 0 | | 968 | | 781 | | 7 | | 20.97 |  |
| 601 | 339 | 0 | | 0 | | 197 | | 0 | | 968 | | 781 | | 14 | | 27.04 |  |
| 602 | 339 | 0 | | 0 | | 197 | | 0 | | 968 | | 781 | | 28 | | 32.04 |  |
| 603 | 339 | 0 | | 0 | | 197 | | 0 | | 968 | | 781 | | 90 | | 35.17 |  |
| 604 | 339 | 0 | | 0 | | 197 | | 0 | | 968 | | 781 | | 180 | | 36.45 |  |
| 605 | 339 | 0 | | 0 | | 197 | | 0 | | 968 | | 781 | | 365 | | 38.89 |  |
| 606 | 236 | 0 | | 0 | | 194 | | 0 | | 968 | | 885 | | 3 | | 6.47 |  |
| 607 | 236 | 0 | | 0 | | 194 | | 0 | | 968 | | 885 | | 14 | | 12.84 |  |
| 608 | 236 | 0 | | 0 | | 194 | | 0 | | 968 | | 885 | | 28 | | 18.42 |  |
| 609 | 236 | 0 | | 0 | | 194 | | 0 | | 968 | | 885 | | 90 | | 21.95 |  |
| 610 | 236 | 0 | | 0 | | 193 | | 0 | | 968 | | 885 | | 180 | | 24.1 |  |
| 611 | 236 | 0 | | 0 | | 193 | | 0 | | 968 | | 885 | | 365 | | 25.08 |  |
| 612 | 277 | 0 | | 0 | | 191 | | 0 | | 968 | | 856 | | 14 | | 21.26 |  |
| 613 | 277 | 0 | | 0 | | 191 | | 0 | | 968 | | 856 | | 28 | | 25.97 |  |
| 614 | 277 | 0 | | 0 | | 191 | | 0 | | 968 | | 856 | | 3 | | 11.36 |  |
| 615 | 277 | 0 | | 0 | | 191 | | 0 | | 968 | | 856 | | 90 | | 31.25 |  |
| 616 | 277 | 0 | | 0 | | 191 | | 0 | | 968 | | 856 | | 180 | | 32.33 |  |
| 617 | 277 | 0 | | 0 | | 191 | | 0 | | 968 | | 856 | | 360 | | 33.7 |  |
| 618 | 254 | 0 | | 0 | | 198 | | 0 | | 968 | | 863 | | 3 | | 9.31 |  |
| 619 | 254 | 0 | | 0 | | 198 | | 0 | | 968 | | 863 | | 90 | | 26.94 |  |
| 620 | 254 | 0 | | 0 | | 198 | | 0 | | 968 | | 863 | | 180 | | 27.63 |  |
| 621 | 254 | 0 | | 0 | | 198 | | 0 | | 968 | | 863 | | 365 | | 29.79 |  |
| 622 | 307 | 0 | | 0 | | 193 | | 0 | | 968 | | 812 | | 180 | | 34.49 |  |
| 623 | 307 | 0 | | 0 | | 193 | | 0 | | 968 | | 812 | | 365 | | 36.15 |  |
| 624 | 307 | 0 | | 0 | | 193 | | 0 | | 968 | | 812 | | 3 | | 12.54 |  |
| 625 | 307 | 0 | | 0 | | 193 | | 0 | | 968 | | 812 | | 28 | | 27.53 |  |
| 626 | 307 | 0 | | 0 | | 193 | | 0 | | 968 | | 812 | | 90 | | 32.92 |  |
| 627 | 236 | 0 | | 0 | | 193 | | 0 | | 968 | | 885 | | 7 | | 9.99 |  |
| 628 | 200 | 0 | | 0 | | 180 | | 0 | | 1125 | | 845 | | 7 | | 7.84 |  |
| 629 | 200 | 0 | | 0 | | 180 | | 0 | | 1125 | | 845 | | 28 | | 12.25 |  |
| 630 | 225 | 0 | | 0 | | 181 | | 0 | | 1113 | | 833 | | 7 | | 11.17 |  |
| 631 | 225 | 0 | | 0 | | 181 | | 0 | | 1113 | | 833 | | 28 | | 17.34 |  |
| 632 | 325 | 0 | | 0 | | 184 | | 0 | | 1063 | | 783 | | 7 | | 17.54 |  |
| 633 | 325 | 0 | | 0 | | 184 | | 0 | | 1063 | | 783 | | 28 | | 30.57 |  |
| 634 | 275 | 0 | | 0 | | 183 | | 0 | | 1088 | | 808 | | 7 | | 14.2 |  |
| 635 | 275 | 0 | | 0 | | 183 | | 0 | | 1088 | | 808 | | 28 | | 24.5 |  |
| 636 | 300 | 0 | | 0 | | 184 | | 0 | | 1075 | | 795 | | 7 | | 15.58 |  |
| 637 | 300 | 0 | | 0 | | 184 | | 0 | | 1075 | | 795 | | 28 | | 26.85 |  |
| 638 | 375 | 0 | | 0 | | 186 | | 0 | | 1038 | | 758 | | 7 | | 26.06 |  |
| 639 | 375 | 0 | | 0 | | 186 | | 0 | | 1038 | | 758 | | 28 | | 38.21 |  |
| 640 | 400 | 0 | | 0 | | 187 | | 0 | | 1025 | | 745 | | 28 | | 43.7 |  |
| 641 | 400 | 0 | | 0 | | 187 | | 0 | | 1025 | | 745 | | 7 | | 30.14 |  |
| 642 | 250 | 0 | | 0 | | 182 | | 0 | | 1100 | | 820 | | 7 | | 12.73 |  |
| 643 | 250 | 0 | | 0 | | 182 | | 0 | | 1100 | | 820 | | 28 | | 20.87 |  |
| 644 | 350 | 0 | | 0 | | 186 | | 0 | | 1050 | | 770 | | 7 | | 20.28 |  |
| 645 | 350 | 0 | | 0 | | 186 | | 0 | | 1050 | | 770 | | 28 | | 34.29 |  |
| 646 | 203.5 | 305.3 | | 0 | | 203.5 | | 0 | | 963.4 | | 630 | | 7 | | 19.54 |  |
| 647 | 250.2 | 166.8 | | 0 | | 203.5 | | 0 | | 977.6 | | 694.1 | | 90 | | 47.71 |  |
| 648 | 157 | 236 | | 0 | | 192 | | 0 | | 935.4 | | 781.2 | | 90 | | 43.38 |  |
| 649 | 141.3 | 212 | | 0 | | 203.5 | | 0 | | 971.8 | | 748.5 | | 28 | | 29.89 |  |
| 650 | 166.8 | 250.2 | | 0 | | 203.5 | | 0 | | 975.6 | | 692.6 | | 3 | | 6.9 |  |
| 651 | 122.6 | 183.9 | | 0 | | 203.5 | | 0 | | 958.2 | | 800.1 | | 90 | | 33.19 |  |
| 652 | 183.9 | 122.6 | | 0 | | 203.5 | | 0 | | 959.2 | | 800 | | 3 | | 4.9 |  |
| 653 | 102 | 153 | | 0 | | 192 | | 0 | | 887 | | 942 | | 3 | | 4.57 |  |
| 654 | 102 | 153 | | 0 | | 192 | | 0 | | 887 | | 942 | | 90 | | 25.46 |  |
| 655 | 122.6 | 183.9 | | 0 | | 203.5 | | 0 | | 958.2 | | 800.1 | | 28 | | 24.29 |  |
| 656 | 166.8 | 250.2 | | 0 | | 203.5 | | 0 | | 975.6 | | 692.6 | | 28 | | 33.95 |  |
| 657 | 200 | 133 | | 0 | | 192 | | 0 | | 965.4 | | 806.2 | | 3 | | 11.41 |  |
| 658 | 108.3 | 162.4 | | 0 | | 203.5 | | 0 | | 938.2 | | 849 | | 28 | | 20.59 |  |
| 659 | 305.3 | 203.5 | | 0 | | 203.5 | | 0 | | 965.4 | | 631 | | 7 | | 25.89 |  |
| 660 | 108.3 | 162.4 | | 0 | | 203.5 | | 0 | | 938.2 | | 849 | | 90 | | 29.23 |  |
| 661 | 116 | 173 | | 0 | | 192 | | 0 | | 909.8 | | 891.9 | | 90 | | 31.02 |  |
| 662 | 141.3 | 212 | | 0 | | 203.5 | | 0 | | 971.8 | | 748.5 | | 7 | | 10.39 |  |
| 663 | 157 | 236 | | 0 | | 192 | | 0 | | 935.4 | | 781.2 | | 28 | | 33.66 |  |
| 664 | 133 | 200 | | 0 | | 192 | | 0 | | 927.4 | | 839.2 | | 28 | | 27.87 |  |
| 665 | 250.2 | 166.8 | | 0 | | 203.5 | | 0 | | 977.6 | | 694.1 | | 7 | | 19.35 |  |
| 666 | 173 | 116 | | 0 | | 192 | | 0 | | 946.8 | | 856.8 | | 7 | | 11.39 |  |
| 667 | 192 | 288 | | 0 | | 192 | | 0 | | 929.8 | | 716.1 | | 3 | | 12.79 |  |
| 668 | 192 | 288 | | 0 | | 192 | | 0 | | 929.8 | | 716.1 | | 28 | | 39.32 |  |
| 669 | 153 | 102 | | 0 | | 192 | | 0 | | 888 | | 943.1 | | 3 | | 4.78 |  |
| 670 | 288 | 192 | | 0 | | 192 | | 0 | | 932 | | 717.8 | | 3 | | 16.11 |  |
| 671 | 305.3 | 203.5 | | 0 | | 203.5 | | 0 | | 965.4 | | 631 | | 28 | | 43.38 |  |
| 672 | 236 | 157 | | 0 | | 192 | | 0 | | 972.6 | | 749.1 | | 7 | | 20.42 |  |
| 673 | 173 | 116 | | 0 | | 192 | | 0 | | 946.8 | | 856.8 | | 3 | | 6.94 |  |
| 674 | 212 | 141.3 | | 0 | | 203.5 | | 0 | | 973.4 | | 750 | | 7 | | 15.03 |  |
| 675 | 236 | 157 | | 0 | | 192 | | 0 | | 972.6 | | 749.1 | | 3 | | 13.57 |  |
| 676 | 183.9 | 122.6 | | 0 | | 203.5 | | 0 | | 959.2 | | 800 | | 90 | | 32.53 |  |
| 677 | 166.8 | 250.2 | | 0 | | 203.5 | | 0 | | 975.6 | | 692.6 | | 7 | | 15.75 |  |
| 678 | 102 | 153 | | 0 | | 192 | | 0 | | 887 | | 942 | | 7 | | 7.68 |  |
| 679 | 288 | 192 | | 0 | | 192 | | 0 | | 932 | | 717.8 | | 28 | | 38.8 |  |
| 680 | 212 | 141.3 | | 0 | | 203.5 | | 0 | | 973.4 | | 750 | | 28 | | 33 |  |
| 681 | 102 | 153 | | 0 | | 192 | | 0 | | 887 | | 942 | | 28 | | 17.28 |  |
| 682 | 173 | 116 | | 0 | | 192 | | 0 | | 946.8 | | 856.8 | | 28 | | 24.28 |  |
| 683 | 183.9 | 122.6 | | 0 | | 203.5 | | 0 | | 959.2 | | 800 | | 28 | | 24.05 |  |
| 684 | 133 | 200 | | 0 | | 192 | | 0 | | 927.4 | | 839.2 | | 90 | | 36.59 |  |
| 685 | 192 | 288 | | 0 | | 192 | | 0 | | 929.8 | | 716.1 | | 90 | | 50.73 |  |
| 686 | 133 | 200 | | 0 | | 192 | | 0 | | 927.4 | | 839.2 | | 7 | | 13.66 |  |
| 687 | 305.3 | 203.5 | | 0 | | 203.5 | | 0 | | 965.4 | | 631 | | 3 | | 14.14 |  |
| 688 | 236 | 157 | | 0 | | 192 | | 0 | | 972.6 | | 749.1 | | 90 | | 47.78 |  |
| 689 | 108.3 | 162.4 | | 0 | | 203.5 | | 0 | | 938.2 | | 849 | | 3 | | 2.33 |  |
| 690 | 157 | 236 | | 0 | | 192 | | 0 | | 935.4 | | 781.2 | | 7 | | 16.89 |  |
| 691 | 288 | 192 | | 0 | | 192 | | 0 | | 932 | | 717.8 | | 7 | | 23.52 |  |
| 692 | 212 | 141.3 | | 0 | | 203.5 | | 0 | | 973.4 | | 750 | | 3 | | 6.81 |  |
| 693 | 212 | 141.3 | | 0 | | 203.5 | | 0 | | 973.4 | | 750 | | 90 | | 39.7 |  |
| 694 | 153 | 102 | | 0 | | 192 | | 0 | | 888 | | 943.1 | | 28 | | 17.96 |  |
| 695 | 236 | 157 | | 0 | | 192 | | 0 | | 972.6 | | 749.1 | | 28 | | 32.88 |  |
| 696 | 116 | 173 | | 0 | | 192 | | 0 | | 909.8 | | 891.9 | | 28 | | 22.35 |  |
| 697 | 183.9 | 122.6 | | 0 | | 203.5 | | 0 | | 959.2 | | 800 | | 7 | | 10.79 |  |
| 698 | 108.3 | 162.4 | | 0 | | 203.5 | | 0 | | 938.2 | | 849 | | 7 | | 7.72 |  |
| 699 | 203.5 | 305.3 | | 0 | | 203.5 | | 0 | | 963.4 | | 630 | | 28 | | 41.68 |  |
| 700 | 203.5 | 305.3 | | 0 | | 203.5 | | 0 | | 963.4 | | 630 | | 3 | | 9.56 |  |
| 701 | 133 | 200 | | 0 | | 192 | | 0 | | 927.4 | | 839.2 | | 3 | | 6.88 |  |
| 702 | 288 | 192 | | 0 | | 192 | | 0 | | 932 | | 717.8 | | 90 | | 50.53 |  |
| 703 | 200 | 133 | | 0 | | 192 | | 0 | | 965.4 | | 806.2 | | 7 | | 17.17 |  |
| 704 | 200 | 133 | | 0 | | 192 | | 0 | | 965.4 | | 806.2 | | 28 | | 30.44 |  |
| 705 | 250.2 | 166.8 | | 0 | | 203.5 | | 0 | | 977.6 | | 694.1 | | 3 | | 9.73 |  |
| 706 | 122.6 | 183.9 | | 0 | | 203.5 | | 0 | | 958.2 | | 800.1 | | 3 | | 3.32 |  |
| 707 | 153 | 102 | | 0 | | 192 | | 0 | | 888 | | 943.1 | | 90 | | 26.32 |  |
| 708 | 200 | 133 | | 0 | | 192 | | 0 | | 965.4 | | 806.2 | | 90 | | 43.25 |  |
| 709 | 116 | 173 | | 0 | | 192 | | 0 | | 909.8 | | 891.9 | | 3 | | 6.28 |  |
| 710 | 173 | 116 | | 0 | | 192 | | 0 | | 946.8 | | 856.8 | | 90 | | 32.1 |  |
| 711 | 250.2 | 166.8 | | 0 | | 203.5 | | 0 | | 977.6 | | 694.1 | | 28 | | 36.96 |  |
| 712 | 305.3 | 203.5 | | 0 | | 203.5 | | 0 | | 965.4 | | 631 | | 90 | | 54.6 |  |
| 713 | 192 | 288 | | 0 | | 192 | | 0 | | 929.8 | | 716.1 | | 7 | | 21.48 |  |
| 714 | 157 | 236 | | 0 | | 192 | | 0 | | 935.4 | | 781.2 | | 3 | | 9.69 |  |
| 715 | 153 | 102 | | 0 | | 192 | | 0 | | 888 | | 943.1 | | 7 | | 8.37 |  |
| 716 | 141.3 | 212 | | 0 | | 203.5 | | 0 | | 971.8 | | 748.5 | | 90 | | 39.66 |  |
| 717 | 116 | 173 | | 0 | | 192 | | 0 | | 909.8 | | 891.9 | | 7 | | 10.09 |  |
| 718 | 141.3 | 212 | | 0 | | 203.5 | | 0 | | 971.8 | | 748.5 | | 3 | | 4.83 |  |
| 719 | 122.6 | 183.9 | | 0 | | 203.5 | | 0 | | 958.2 | | 800.1 | | 7 | | 10.35 |  |
| 720 | 166.8 | 250.2 | | 0 | | 203.5 | | 0 | | 975.6 | | 692.6 | | 90 | | 43.57 |  |
| 721 | 203.5 | 305.3 | | 0 | | 203.5 | | 0 | | 963.4 | | 630 | | 90 | | 51.86 |  |
| 722 | 310 | 0 | | 0 | | 192 | | 0 | | 1012 | | 830 | | 3 | | 11.85 |  |
| 723 | 310 | 0 | | 0 | | 192 | | 0 | | 1012 | | 830 | | 7 | | 17.24 |  |
| 724 | 310 | 0 | | 0 | | 192 | | 0 | | 1012 | | 830 | | 28 | | 27.83 |  |
| 725 | 310 | 0 | | 0 | | 192 | | 0 | | 1012 | | 830 | | 90 | | 35.76 |  |
| 726 | 310 | 0 | | 0 | | 192 | | 0 | | 1012 | | 830 | | 120 | | 38.7 |  |
| 727 | 331 | 0 | | 0 | | 192 | | 0 | | 1025 | | 821 | | 3 | | 14.31 |  |
| 728 | 331 | 0 | | 0 | | 192 | | 0 | | 1025 | | 821 | | 7 | | 17.44 |  |
| 729 | 331 | 0 | | 0 | | 192 | | 0 | | 1025 | | 821 | | 28 | | 31.74 |  |
| 730 | 331 | 0 | | 0 | | 192 | | 0 | | 1025 | | 821 | | 90 | | 37.91 |  |
| 731 | 331 | 0 | | 0 | | 192 | | 0 | | 1025 | | 821 | | 120 | | 39.38 |  |
| 732 | 349 | 0 | | 0 | | 192 | | 0 | | 1056 | | 809 | | 3 | | 15.87 |  |
| 733 | 349 | 0 | | 0 | | 192 | | 0 | | 1056 | | 809 | | 7 | | 9.01 |  |
| 734 | 349 | 0 | | 0 | | 192 | | 0 | | 1056 | | 809 | | 28 | | 33.61 |  |
| 735 | 349 | 0 | | 0 | | 192 | | 0 | | 1056 | | 809 | | 90 | | 40.66 |  |
| 736 | 349 | 0 | | 0 | | 192 | | 0 | | 1056 | | 809 | | 120 | | 40.86 |  |
| 737 | 238 | 0 | | 0 | | 186 | | 0 | | 1119 | | 789 | | 7 | | 12.05 |  |
| 738 | 238 | 0 | | 0 | | 186 | | 0 | | 1119 | | 789 | | 28 | | 17.54 |  |
| 739 | 296 | 0 | | 0 | | 186 | | 0 | | 1090 | | 769 | | 7 | | 18.91 |  |
| 740 | 296 | 0 | | 0 | | 186 | | 0 | | 1090 | | 769 | | 28 | | 25.18 |  |
| 741 | 297 | 0 | | 0 | | 186 | | 0 | | 1040 | | 734 | | 7 | | 30.96 |  |
| 742 | 480 | 0 | | 0 | | 192 | | 0 | | 936 | | 721 | | 28 | | 43.89 |  |
| 743 | 480 | 0 | | 0 | | 192 | | 0 | | 936 | | 721 | | 90 | | 54.28 |  |
| 744 | 397 | 0 | | 0 | | 186 | | 0 | | 1040 | | 734 | | 28 | | 36.94 |  |
| 745 | 281 | 0 | | 0 | | 186 | | 0 | | 1104 | | 774 | | 7 | | 14.5 |  |
| 746 | 281 | 0 | | 0 | | 185 | | 0 | | 1104 | | 774 | | 28 | | 22.44 |  |
| 747 | 500 | 0 | | 0 | | 200 | | 0 | | 1125 | | 613 | | 1 | | 12.64 |  |
| 748 | 500 | 0 | | 0 | | 200 | | 0 | | 1125 | | 613 | | 3 | | 26.06 |  |
| 749 | 500 | 0 | | 0 | | 200 | | 0 | | 1125 | | 613 | | 7 | | 33.21 |  |
| 750 | 500 | 0 | | 0 | | 200 | | 0 | | 1125 | | 613 | | 14 | | 36.94 |  |
| 751 | 500 | 0 | | 0 | | 200 | | 0 | | 1125 | | 613 | | 28 | | 44.09 |  |
| 752 | 540 | 0 | | 0 | | 173 | | 0 | | 1125 | | 613 | | 7 | | 52.61 |  |
| 753 | 540 | 0 | | 0 | | 173 | | 0 | | 1125 | | 613 | | 14 | | 59.76 |  |
| 754 | 540 | 0 | | 0 | | 173 | | 0 | | 1125 | | 613 | | 28 | | 67.31 |  |
| 755 | 540 | 0 | | 0 | | 173 | | 0 | | 1125 | | 613 | | 90 | | 69.66 |  |
| 756 | 540 | 0 | | 0 | | 173 | | 0 | | 1125 | | 613 | | 180 | | 71.62 |  |
| 757 | 540 | 0 | | 0 | | 173 | | 0 | | 1125 | | 613 | | 270 | | 74.17 |  |
| 758 | 350 | 0 | | 0 | | 203 | | 0 | | 974 | | 775 | | 7 | | 18.13 |  |
| 759 | 350 | 0 | | 0 | | 203 | | 0 | | 974 | | 775 | | 14 | | 22.53 |  |
| 760 | 350 | 0 | | 0 | | 203 | | 0 | | 974 | | 775 | | 28 | | 27.34 |  |
| 761 | 350 | 0 | | 0 | | 203 | | 0 | | 974 | | 775 | | 56 | | 29.98 |  |
| 762 | 350 | 0 | | 0 | | 203 | | 0 | | 974 | | 775 | | 90 | | 31.35 |  |
| 763 | 350 | 0 | | 0 | | 203 | | 0 | | 974 | | 775 | | 180 | | 32.72 |  |
| 764 | 385 | 0 | | 0 | | 186 | | 0 | | 966 | | 763 | | 1 | | 6.27 |  |
| 765 | 385 | 0 | | 0 | | 186 | | 0 | | 966 | | 763 | | 3 | | 14.7 |  |
| 766 | 385 | 0 | | 0 | | 186 | | 0 | | 966 | | 763 | | 7 | | 23.22 |  |
| 767 | 385 | 0 | | 0 | | 186 | | 0 | | 966 | | 763 | | 14 | | 27.92 |  |
| 768 | 385 | 0 | | 0 | | 186 | | 0 | | 966 | | 763 | | 28 | | 31.35 |  |
| 769 | 331 | 0 | | 0 | | 192 | | 0 | | 978 | | 825 | | 180 | | 39 |  |
| 770 | 331 | 0 | | 0 | | 192 | | 0 | | 978 | | 825 | | 360 | | 41.24 |  |
| 771 | 349 | 0 | | 0 | | 192 | | 0 | | 1047 | | 806 | | 3 | | 14.99 |  |
| 772 | 331 | 0 | | 0 | | 192 | | 0 | | 978 | | 825 | | 3 | | 13.52 |  |
| 773 | 382 | 0 | | 0 | | 186 | | 0 | | 1047 | | 739 | | 7 | | 24 |  |
| 774 | 382 | 0 | | 0 | | 186 | | 0 | | 1047 | | 739 | | 28 | | 37.42 |  |
| 775 | 382 | 0 | | 0 | | 186 | | 0 | | 1111 | | 784 | | 7 | | 11.47 |  |
| 776 | 281 | 0 | | 0 | | 186 | | 0 | | 1104 | | 774 | | 28 | | 22.44 |  |
| 777 | 339 | 0 | | 0 | | 185 | | 0 | | 1069 | | 754 | | 7 | | 21.16 |  |
| 778 | 339 | 0 | | 0 | | 185 | | 0 | | 1069 | | 754 | | 28 | | 31.84 |  |
| 779 | 295 | 0 | | 0 | | 185 | | 0 | | 1069 | | 769 | | 7 | | 14.8 |  |
| 780 | 295 | 0 | | 0 | | 185 | | 0 | | 1069 | | 769 | | 28 | | 25.18 |  |
| 781 | 238 | 0 | | 0 | | 185 | | 0 | | 1118 | | 789 | | 28 | | 17.54 |  |
| 782 | 296 | 0 | | 0 | | 192 | | 0 | | 1085 | | 765 | | 7 | | 14.2 |  |
| 783 | 296 | 0 | | 0 | | 192 | | 0 | | 1085 | | 765 | | 28 | | 21.65 |  |
| 784 | 296 | 0 | | 0 | | 192 | | 0 | | 1085 | | 765 | | 90 | | 29.39 |  |
| 785 | 331 | 0 | | 0 | | 192 | | 0 | | 879 | | 825 | | 3 | | 13.52 |  |
| 786 | 331 | 0 | | 0 | | 192 | | 0 | | 978 | | 825 | | 7 | | 16.26 |  |
| 787 | 331 | 0 | | 0 | | 192 | | 0 | | 978 | | 825 | | 28 | | 31.45 |  |
| 788 | 331 | 0 | | 0 | | 192 | | 0 | | 978 | | 825 | | 90 | | 37.23 |  |
| 789 | 349 | 0 | | 0 | | 192 | | 0 | | 1047 | | 806 | | 7 | | 18.13 |  |
| 790 | 349 | 0 | | 0 | | 192 | | 0 | | 1047 | | 806 | | 28 | | 32.72 |  |
| 791 | 349 | 0 | | 0 | | 192 | | 0 | | 1047 | | 806 | | 90 | | 39.49 |  |
| 792 | 349 | 0 | | 0 | | 192 | | 0 | | 1047 | | 806 | | 180 | | 41.05 |  |
| 793 | 349 | 0 | | 0 | | 192 | | 0 | | 1047 | | 806 | | 360 | | 42.13 |  |
| 794 | 302 | 0 | | 0 | | 203 | | 0 | | 974 | | 817 | | 14 | | 18.13 |  |
| 795 | 302 | 0 | | 0 | | 203 | | 0 | | 974 | | 817 | | 180 | | 26.74 |  |
| 796 | 525 | 0 | | 0 | | 189 | | 0 | | 1125 | | 613 | | 180 | | 61.92 |  |
| 797 | 500 | 0 | | 0 | | 200 | | 0 | | 1125 | | 613 | | 90 | | 47.22 |  |
| 798 | 500 | 0 | | 0 | | 200 | | 0 | | 1125 | | 613 | | 180 | | 51.04 |  |
| 799 | 500 | 0 | | 0 | | 200 | | 0 | | 1125 | | 613 | | 270 | | 55.16 |  |
| 800 | 540 | 0 | | 0 | | 173 | | 0 | | 1125 | | 613 | | 3 | | 41.64 |  |
| 801 | 252 | 0 | | 0 | | 185 | | 0 | | 1111 | | 784 | | 7 | | 13.71 |  |
| 802 | 252 | 0 | | 0 | | 185 | | 0 | | 1111 | | 784 | | 28 | | 19.69 |  |
| 803 | 339 | 0 | | 0 | | 185 | | 0 | | 1060 | | 754 | | 28 | | 31.65 |  |
| 804 | 393 | 0 | | 0 | | 192 | | 0 | | 940 | | 758 | | 3 | | 19.11 |  |
| 805 | 393 | 0 | | 0 | | 192 | | 0 | | 940 | | 758 | | 28 | | 39.58 |  |
| 806 | 393 | 0 | | 0 | | 192 | | 0 | | 940 | | 758 | | 90 | | 48.79 |  |
| 807 | 382 | 0 | | 0 | | 185 | | 0 | | 1047 | | 739 | | 7 | | 24 |  |
| 808 | 382 | 0 | | 0 | | 185 | | 0 | | 1047 | | 739 | | 28 | | 37.42 |  |
| 809 | 252 | 0 | | 0 | | 186 | | 0 | | 1111 | | 784 | | 7 | | 11.47 |  |
| 810 | 252 | 0 | | 0 | | 185 | | 0 | | 1111 | | 784 | | 28 | | 19.69 |  |
| 811 | 310 | 0 | | 0 | | 192 | | 0 | | 970 | | 850 | | 7 | | 14.99 |  |
| 812 | 310 | 0 | | 0 | | 192 | | 0 | | 970 | | 850 | | 28 | | 27.92 |  |
| 813 | 310 | 0 | | 0 | | 192 | | 0 | | 970 | | 850 | | 90 | | 34.68 |  |
| 814 | 310 | 0 | | 0 | | 192 | | 0 | | 970 | | 850 | | 180 | | 37.33 |  |
| 815 | 310 | 0 | | 0 | | 192 | | 0 | | 970 | | 850 | | 360 | | 38.11 |  |
| 816 | 525 | 0 | | 0 | | 189 | | 0 | | 1125 | | 613 | | 3 | | 33.8 |  |
| 817 | 525 | 0 | | 0 | | 189 | | 0 | | 1125 | | 613 | | 7 | | 42.42 |  |
| 818 | 525 | 0 | | 0 | | 189 | | 0 | | 1125 | | 613 | | 14 | | 48.4 |  |
| 819 | 525 | 0 | | 0 | | 189 | | 0 | | 1125 | | 613 | | 28 | | 55.94 |  |
| 820 | 525 | 0 | | 0 | | 189 | | 0 | | 1125 | | 613 | | 90 | | 58.78 |  |
| 821 | 525 | 0 | | 0 | | 189 | | 0 | | 1125 | | 613 | | 270 | | 67.11 |  |
| 822 | 322 | 0 | | 0 | | 203 | | 0 | | 974 | | 800 | | 14 | | 20.77 |  |
| 823 | 322 | 0 | | 0 | | 203 | | 0 | | 974 | | 800 | | 28 | | 25.18 |  |
| 824 | 322 | 0 | | 0 | | 203 | | 0 | | 974 | | 800 | | 180 | | 29.59 |  |
| 825 | 302 | 0 | | 0 | | 203 | | 0 | | 974 | | 817 | | 28 | | 21.75 |  |
| 826 | 397 | 0 | | 0 | | 185 | | 0 | | 1040 | | 734 | | 28 | | 39.09 |  |
| 827 | 480 | 0 | | 0 | | 192 | | 0 | | 936 | | 721 | | 3 | | 24.39 |  |
| 828 | 522 | 0 | | 0 | | 146 | | 0 | | 896 | | 896 | | 7 | | 50.51 |  |
| 829 | 522 | 0 | | 0 | | 146 | | 0 | | 896 | | 896 | | 28 | | 74.99 |  |
| 830 | 273 | 105 | | 82 | | 210 | | 9 | | 904 | | 680 | | 28 | | 37.17 |  |
| 831 | 162 | 190 | | 148 | | 179 | | 19 | | 838 | | 741 | | 28 | | 33.76 |  |
| 832 | 154 | 144 | | 112 | | 220 | | 10 | | 923 | | 658 | | 28 | | 16.5 |  |
| 833 | 147 | 115 | | 89 | | 202 | | 9 | | 860 | | 829 | | 28 | | 19.99 |  |
| 834 | 152 | 178 | | 139 | | 168 | | 18 | | 944 | | 695 | | 28 | | 36.35 |  |
| 835 | 310 | 143 | | 111 | | 168 | | 22 | | 914 | | 651 | | 28 | | 33.69 |  |
| 836 | 144 | 0 | | 175 | | 158 | | 18 | | 943 | | 844 | | 28 | | 15.42 |  |
| 837 | 304 | 140 | | 0 | | 214 | | 6 | | 895 | | 722 | | 28 | | 33.42 |  |
| 838 | 374 | 0 | | 0 | | 190 | | 7 | | 1013 | | 730 | | 28 | | 39.05 |  |
| 839 | 159 | 149 | | 116 | | 175 | | 15 | | 953 | | 720 | | 28 | | 27.68 |  |
| 840 | 153 | 239 | | 0 | | 200 | | 6 | | 1002 | | 684 | | 28 | | 26.86 |  |
| 841 | 310 | 143 | | 0 | | 168 | | 10 | | 914 | | 804 | | 28 | | 45.3 |  |
| 842 | 305 | 0 | | 100 | | 196 | | 10 | | 959 | | 705 | | 28 | | 30.12 |  |
| 843 | 151 | 0 | | 184 | | 167 | | 12 | | 991 | | 772 | | 28 | | 15.57 |  |
| 844 | 142 | 167 | | 130 | | 174 | | 11 | | 883 | | 785 | | 28 | | 44.61 |  |
| 845 | 298 | 137 | | 107 | | 201 | | 6 | | 878 | | 655 | | 28 | | 53.52 |  |
| 846 | 321 | 164 | | 0 | | 190 | | 5 | | 870 | | 774 | | 28 | | 57.21 |  |
| 847 | 366 | 187 | | 0 | | 191 | | 7 | | 824 | | 757 | | 28 | | 65.91 |  |
| 848 | 280 | 129 | | 100 | | 172 | | 9 | | 825 | | 805 | | 28 | | 52.82 |  |
| 849 | 252 | 97 | | 76 | | 194 | | 8 | | 835 | | 821 | | 28 | | 33.4 |  |
| 850 | 165 | 0 | | 150 | | 182 | | 12 | | 1023 | | 729 | | 28 | | 18.03 |  |
| 851 | 156 | 243 | | 0 | | 180 | | 11 | | 1022 | | 698 | | 28 | | 37.36 |  |
| 852 | 160 | 188 | | 146 | | 203 | | 11 | | 829 | | 710 | | 28 | | 32.84 |  |
| 853 | 298 | 0 | | 107 | | 186 | | 6 | | 879 | | 815 | | 28 | | 42.64 |  |
| 854 | 318 | 0 | | 126 | | 210 | | 6 | | 861 | | 737 | | 28 | | 40.06 |  |
| 855 | 287 | 121 | | 94 | | 188 | | 9 | | 904 | | 696 | | 28 | | 41.94 |  |
| 856 | 326 | 166 | | 0 | | 174 | | 9 | | 882 | | 790 | | 28 | | 61.23 |  |
| 857 | 356 | 0 | | 142 | | 193 | | 11 | | 801 | | 778 | | 28 | | 40.87 |  |
| 858 | 132 | 207 | | 161 | | 179 | | 5 | | 867 | | 736 | | 28 | | 33.3 |  |
| 859 | 322 | 149 | | 0 | | 186 | | 8 | | 951 | | 709 | | 28 | | 52.42 |  |
| 860 | 164 | 0 | | 200 | | 181 | | 13 | | 849 | | 846 | | 28 | | 15.09 |  |
| 861 | 314 | 0 | | 113 | | 170 | | 10 | | 925 | | 783 | | 28 | | 38.46 |  |
| 862 | 321 | 0 | | 128 | | 182 | | 11 | | 870 | | 780 | | 28 | | 37.26 |  |
| 863 | 140 | 164 | | 128 | | 237 | | 6 | | 869 | | 656 | | 28 | | 35.23 |  |
| 864 | 288 | 121 | | 0 | | 177 | | 7 | | 908 | | 829 | | 28 | | 42.13 |  |
| 865 | 298 | 0 | | 107 | | 210 | | 11 | | 880 | | 744 | | 28 | | 31.87 |  |
| 866 | 265 | 111 | | 86 | | 195 | | 6 | | 833 | | 790 | | 28 | | 41.54 |  |
| 867 | 160 | 250 | | 0 | | 168 | | 12 | | 1049 | | 688 | | 28 | | 39.45 |  |
| 868 | 166 | 260 | | 0 | | 183 | | 13 | | 859 | | 827 | | 28 | | 37.91 |  |
| 869 | 276 | 116 | | 90 | | 180 | | 9 | | 870 | | 768 | | 28 | | 44.28 |  |
| 870 | 322 | 0 | | 116 | | 196 | | 10 | | 818 | | 813 | | 28 | | 31.18 |  |
| 871 | 149 | 139 | | 109 | | 193 | | 6 | | 892 | | 780 | | 28 | | 23.69 |  |
| 872 | 159 | 187 | | 0 | | 176 | | 11 | | 990 | | 789 | | 28 | | 32.76 |  |
| 873 | 261 | 100 | | 78 | | 201 | | 9 | | 864 | | 761 | | 28 | | 32.4 |  |
| 874 | 237 | 92 | | 71 | | 247 | | 6 | | 853 | | 695 | | 28 | | 28.63 |  |
| 875 | 313 | 0 | | 113 | | 178 | | 8 | | 1002 | | 689 | | 28 | | 36.8 |  |
| 876 | 155 | 183 | | 0 | | 193 | | 9 | | 1047 | | 697 | | 28 | | 18.28 |  |
| 877 | 146 | 230 | | 0 | | 202 | | 3 | | 827 | | 872 | | 28 | | 33.06 |  |
| 878 | 296 | 0 | | 107 | | 221 | | 11 | | 819 | | 778 | | 28 | | 31.42 |  |
| 879 | 133 | 210 | | 0 | | 196 | | 3 | | 949 | | 795 | | 28 | | 31.03 |  |
| 880 | 313 | 145 | | 0 | | 178 | | 8 | | 867 | | 824 | | 28 | | 44.39 |  |
| 881 | 152 | 0 | | 112 | | 184 | | 8 | | 992 | | 816 | | 28 | | 12.18 |  |
| 882 | 153 | 145 | | 113 | | 178 | | 8 | | 1002 | | 689 | | 28 | | 25.56 |  |
| 883 | 140 | 133 | | 103 | | 200 | | 7 | | 916 | | 753 | | 28 | | 36.44 |  |
| 884 | 149 | 236 | | 0 | | 176 | | 13 | | 847 | | 893 | | 28 | | 32.96 |  |
| 885 | 300 | 0 | | 120 | | 212 | | 10 | | 878 | | 728 | | 28 | | 23.84 |  |
| 886 | 153 | 145 | | 113 | | 178 | | 8 | | 867 | | 824 | | 28 | | 26.23 |  |
| 887 | 148 | 0 | | 137 | | 158 | | 16 | | 1002 | | 830 | | 28 | | 17.95 |  |
| 888 | 326 | 0 | | 138 | | 199 | | 11 | | 801 | | 792 | | 28 | | 40.68 |  |
| 889 | 153 | 145 | | 0 | | 178 | | 8 | | 1000 | | 822 | | 28 | | 19.01 |  |
| 890 | 262 | 111 | | 86 | | 195 | | 5 | | 895 | | 733 | | 28 | | 33.72 |  |
| 891 | 158 | 0 | | 195 | | 220 | | 11 | | 898 | | 713 | | 28 | | 8.54 |  |
| 892 | 151 | 0 | | 185 | | 167 | | 16 | | 1074 | | 678 | | 28 | | 13.46 |  |
| 893 | 273 | 0 | | 90 | | 199 | | 11 | | 931 | | 762 | | 28 | | 32.24 |  |
| 894 | 149 | 118 | | 92 | | 183 | | 7 | | 953 | | 780 | | 28 | | 23.52 |  |
| 895 | 143 | 169 | | 143 | | 191 | | 8 | | 967 | | 643 | | 28 | | 29.72 |  |
| 896 | 260 | 101 | | 78 | | 171 | | 10 | | 936 | | 763 | | 28 | | 49.77 |  |
| 897 | 313 | 161 | | 0 | | 178 | | 10 | | 917 | | 759 | | 28 | | 52.44 |  |
| 898 | 284 | 120 | | 0 | | 168 | | 7 | | 970 | | 794 | | 28 | | 40.93 |  |
| 899 | 336 | 0 | | 0 | | 182 | | 3 | | 986 | | 817 | | 28 | | 44.86 |  |
| 900 | 145 | 0 | | 134 | | 181 | | 11 | | 979 | | 812 | | 28 | | 13.2 |  |
| 901 | 150 | 237 | | 0 | | 174 | | 12 | | 1069 | | 675 | | 28 | | 37.43 |  |
| 902 | 144 | 170 | | 133 | | 192 | | 8 | | 814 | | 805 | | 28 | | 29.87 |  |
| 903 | 331 | 170 | | 0 | | 195 | | 8 | | 811 | | 802 | | 28 | | 56.61 |  |
| 904 | 155 | 0 | | 143 | | 193 | | 9 | | 1047 | | 697 | | 28 | | 12.46 |  |
| 905 | 155 | 183 | | 0 | | 193 | | 9 | | 877 | | 868 | | 28 | | 23.79 |  |
| 906 | 135 | 0 | | 166 | | 180 | | 10 | | 961 | | 805 | | 28 | | 13.29 |  |
| 907 | 266 | 112 | | 87 | | 178 | | 10 | | 910 | | 745 | | 28 | | 39.42 |  |
| 908 | 314 | 145 | | 113 | | 179 | | 8 | | 869 | | 690 | | 28 | | 46.23 |  |
| 909 | 313 | 145 | | 0 | | 127 | | 8 | | 1000 | | 822 | | 28 | | 44.52 |  |
| 910 | 146 | 173 | | 0 | | 182 | | 3 | | 986 | | 817 | | 28 | | 23.74 |  |
| 911 | 144 | 136 | | 106 | | 178 | | 7 | | 941 | | 774 | | 28 | | 26.14 |  |
| 912 | 148 | 0 | | 182 | | 181 | | 15 | | 839 | | 884 | | 28 | | 15.52 |  |
| 913 | 277 | 117 | | 91 | | 191 | | 7 | | 946 | | 666 | | 28 | | 43.57 |  |
| 914 | 298 | 0 | | 107 | | 164 | | 13 | | 953 | | 784 | | 28 | | 35.86 |  |
| 915 | 313 | 145 | | 0 | | 178 | | 8 | | 1002 | | 689 | | 28 | | 41.05 |  |
| 916 | 155 | 184 | | 143 | | 194 | | 9 | | 880 | | 699 | | 28 | | 28.99 |  |
| 917 | 289 | 134 | | 0 | | 195 | | 6 | | 924 | | 760 | | 28 | | 46.24 |  |
| 918 | 148 | 175 | | 0 | | 171 | | 2 | | 1000 | | 828 | | 28 | | 26.92 |  |
| 919 | 145 | 0 | | 179 | | 202 | | 8 | | 824 | | 869 | | 28 | | 10.54 |  |
| 920 | 313 | 0 | | 0 | | 178 | | 8 | | 1000 | | 822 | | 28 | | 25.1 |  |
| 921 | 136 | 162 | | 126 | | 172 | | 10 | | 923 | | 764 | | 28 | | 29.07 |  |
| 922 | 155 | 0 | | 143 | | 193 | | 9 | | 877 | | 868 | | 28 | | 9.74 |  |
| 923 | 255 | 99 | | 77 | | 189 | | 6 | | 919 | | 749 | | 28 | | 33.8 |  |
| 924 | 162 | 207 | | 172 | | 216 | | 10 | | 822 | | 638 | | 28 | | 39.84 |  |
| 925 | 136 | 196 | | 98 | | 199 | | 6 | | 847 | | 783 | | 28 | | 26.97 |  |
| 926 | 164 | 163 | | 128 | | 197 | | 8 | | 961 | | 641 | | 28 | | 27.23 |  |
| 927 | 162 | 214 | | 164 | | 202 | | 10 | | 820 | | 680 | | 28 | | 30.65 |  |
| 928 | 157 | 214 | | 152 | | 200 | | 9 | | 819 | | 704 | | 28 | | 33.05 |  |
| 929 | 149 | 153 | | 194 | | 192 | | 8 | | 935 | | 623 | | 28 | | 24.58 |  |
| 930 | 135 | 105 | | 193 | | 196 | | 6 | | 965 | | 643 | | 28 | | 21.91 |  |
| 931 | 159 | 209 | | 161 | | 201 | | 7 | | 848 | | 669 | | 28 | | 30.88 |  |
| 932 | 144 | 15 | | 195 | | 176 | | 6 | | 1021 | | 709 | | 28 | | 15.34 |  |
| 933 | 154 | 174 | | 185 | | 228 | | 7 | | 845 | | 612 | | 28 | | 24.34 |  |
| 934 | 167 | 187 | | 195 | | 185 | | 7 | | 898 | | 636 | | 28 | | 23.89 |  |
| 935 | 184 | 86 | | 190 | | 213 | | 6 | | 923 | | 623 | | 28 | | 22.93 |  |
| 936 | 156 | 178 | | 187 | | 221 | | 7 | | 854 | | 614 | | 28 | | 29.41 |  |
| 937 | 236.9 | 91.7 | | 71.5 | | 246.9 | | 6 | | 852.9 | | 695.4 | | 28 | | 28.63 |  |
| 938 | 313.3 | 0 | | 113 | | 178.5 | | 8 | | 1001.9 | | 688.7 | | 28 | | 36.8 |  |
| 939 | 154.8 | 183.4 | | 0 | | 193.3 | | 9.1 | | 1047.4 | | 696.7 | | 28 | | 18.29 |  |
| 940 | 145.9 | 230.5 | | 0 | | 202.5 | | 3.4 | | 827 | | 871.8 | | 28 | | 32.72 |  |
| 941 | 296 | 0 | | 106.7 | | 221.4 | | 10.5 | | 819.2 | | 778.4 | | 28 | | 31.42 |  |
| 942 | 133.1 | 210.2 | | 0 | | 195.7 | | 3.1 | | 949.4 | | 795.3 | | 28 | | 28.94 |  |
| 943 | 313.3 | 145 | | 0 | | 178.5 | | 8 | | 867.2 | | 824 | | 28 | | 40.93 |  |
| 944 | 151.6 | 0 | | 111.9 | | 184.4 | | 7.9 | | 992 | | 815.9 | | 28 | | 12.18 |  |
| 945 | 153.1 | 145 | | 113 | | 178.5 | | 8 | | 1001.9 | | 688.7 | | 28 | | 25.56 |  |
| 946 | 139.9 | 132.6 | | 103.3 | | 200.3 | | 7.4 | | 916 | | 753.4 | | 28 | | 36.44 |  |
| 947 | 149.5 | 236 | | 0 | | 175.8 | | 12.6 | | 846.8 | | 892.7 | | 28 | | 32.96 |  |
| 948 | 299.8 | 0 | | 119.8 | | 211.5 | | 9.9 | | 878.2 | | 727.6 | | 28 | | 23.84 |  |
| 949 | 153.1 | 145 | | 113 | | 178.5 | | 8 | | 867.2 | | 824 | | 28 | | 26.23 |  |
| 950 | 148.1 | 0 | | 136.6 | | 158.1 | | 16.1 | | 1001.8 | | 830.1 | | 28 | | 17.96 |  |
| 951 | 326.5 | 0 | | 137.9 | | 199 | | 10.8 | | 801.1 | | 792.5 | | 28 | | 38.63 |  |
| 952 | 152.7 | 144.7 | | 0 | | 178.1 | | 8 | | 999.7 | | 822.2 | | 28 | | 19.01 |  |
| 953 | 261.9 | 110.5 | | 86.1 | | 195.4 | | 5 | | 895.2 | | 732.6 | | 28 | | 33.72 |  |
| 954 | 158.4 | 0 | | 194.9 | | 219.7 | | 11 | | 897.7 | | 712.9 | | 28 | | 8.54 |  |
| 955 | 150.7 | 0 | | 185.3 | | 166.7 | | 15.6 | | 1074.5 | | 678 | | 28 | | 13.46 |  |
| 956 | 272.6 | 0 | | 89.6 | | 198.7 | | 10.6 | | 931.3 | | 762.2 | | 28 | | 32.25 |  |
| 957 | 149 | 117.6 | | 91.7 | | 182.9 | | 7.1 | | 953.4 | | 780.3 | | 28 | | 23.52 |  |
| 958 | 143 | 169.4 | | 142.7 | | 190.7 | | 8.4 | | 967.4 | | 643.5 | | 28 | | 29.73 |  |
| 959 | 259.9 | 100.6 | | 78.4 | | 170.6 | | 10.4 | | 935.7 | | 762.9 | | 28 | | 49.77 |  |
| 960 | 312.9 | 160.5 | | 0 | | 177.6 | | 9.6 | | 916.6 | | 759.5 | | 28 | | 52.45 |  |
| 961 | 284 | 119.7 | | 0 | | 168.3 | | 7.2 | | 970.4 | | 794.2 | | 28 | | 40.93 |  |
| 962 | 336.5 | 0 | | 0 | | 181.9 | | 3.4 | | 985.8 | | 816.8 | | 28 | | 44.87 |  |
| 963 | 144.8 | 0 | | 133.6 | | 180.8 | | 11.1 | | 979.5 | | 811.5 | | 28 | | 13.2 |  |
| 964 | 150 | 236.8 | | 0 | | 173.8 | | 11.9 | | 1069.3 | | 674.8 | | 28 | | 37.43 |  |
| 965 | 143.7 | 170.2 | | 132.6 | | 191.6 | | 8.5 | | 814.1 | | 805.3 | | 28 | | 29.87 |  |
| 966 | 330.5 | 169.6 | | 0 | | 194.9 | | 8.1 | | 811 | | 802.3 | | 28 | | 56.62 |  |
| 967 | 154.8 | 0 | | 142.8 | | 193.3 | | 9.1 | | 1047.4 | | 696.7 | | 28 | | 12.46 |  |
| 968 | 154.8 | 183.4 | | 0 | | 193.3 | | 9.1 | | 877.2 | | 867.7 | | 28 | | 23.79 |  |
| 969 | 134.7 | 0 | | 165.7 | | 180.2 | | 10 | | 961 | | 804.9 | | 28 | | 13.29 |  |
| 970 | 266.2 | 112.3 | | 87.5 | | 177.9 | | 10.4 | | 909.7 | | 744.5 | | 28 | | 39.42 |  |
| 971 | 314 | 145.3 | | 113.2 | | 178.9 | | 8 | | 869.1 | | 690.2 | | 28 | | 46.23 |  |
| 972 | 312.7 | 144.7 | | 0 | | 127.3 | | 8 | | 999.7 | | 822.2 | | 28 | | 44.52 |  |
| 973 | 145.7 | 172.6 | | 0 | | 181.9 | | 3.4 | | 985.8 | | 816.8 | | 28 | | 23.74 |  |
| 974 | 143.8 | 136.3 | | 106.2 | | 178.1 | | 7.5 | | 941.5 | | 774.3 | | 28 | | 26.15 |  |
| 975 | 148.1 | 0 | | 182.1 | | 181.4 | | 15 | | 838.9 | | 884.3 | | 28 | | 15.53 |  |
| 976 | 277 | 116.8 | | 91 | | 190.6 | | 7 | | 946.5 | | 665.6 | | 28 | | 43.58 |  |
| 977 | 298.1 | 0 | | 107.5 | | 163.6 | | 12.8 | | 953.2 | | 784 | | 28 | | 35.87 |  |
| 978 | 313.3 | 145 | | 0 | | 178.5 | | 8 | | 1001.9 | | 688.7 | | 28 | | 41.05 |  |
| 979 | 155.2 | 183.9 | | 143.2 | | 193.8 | | 9.2 | | 879.6 | | 698.5 | | 28 | | 28.99 |  |
| 980 | 289 | 133.7 | | 0 | | 194.9 | | 5.5 | | 924.1 | | 760.1 | | 28 | | 46.25 |  |
| 981 | 147.8 | 175.1 | | 0 | | 171.2 | | 2.2 | | 1000 | | 828.5 | | 28 | | 26.92 |  |
| 982 | 145.4 | 0 | | 178.9 | | 201.7 | | 7.8 | | 824 | | 868.7 | | 28 | | 10.54 |  |
| 983 | 312.7 | 0 | | 0 | | 178.1 | | 8 | | 999.7 | | 822.2 | | 28 | | 25.1 |  |
| 984 | 136.4 | 161.6 | | 125.8 | | 171.6 | | 10.4 | | 922.6 | | 764.4 | | 28 | | 29.07 |  |
| 985 | 154.8 | 0 | | 142.8 | | 193.3 | | 9.1 | | 877.2 | | 867.7 | | 28 | | 9.74 |  |
| 986 | 255.3 | 98.8 | | 77 | | 188.6 | | 6.5 | | 919 | | 749.3 | | 28 | | 33.8 |  |
| 987 | 272.8 | 105.1 | | 81.8 | | 209.7 | | 9 | | 904 | | 679.7 | | 28 | | 37.17 |  |
| 988 | 162 | 190.1 | | 148.1 | | 178.8 | | 18.8 | | 838.1 | | 741.4 | | 28 | | 33.76 |  |
| 989 | 153.6 | 144.2 | | 112.3 | | 220.1 | | 10.1 | | 923.2 | | 657.9 | | 28 | | 16.5 |  |
| 990 | 146.5 | 114.6 | | 89.3 | | 201.9 | | 8.8 | | 860 | | 829.5 | | 28 | | 19.99 |  |
| 991 | 151.8 | 178.1 | | 138.7 | | 167.5 | | 18.3 | | 944 | | 694.6 | | 28 | | 36.35 |  |
| 992 | 309.9 | 142.8 | | 111.2 | | 167.8 | | 22.1 | | 913.9 | | 651.2 | | 28 | | 38.22 |  |
| 993 | 143.6 | 0 | | 174.9 | | 158.4 | | 17.9 | | 942.7 | | 844.5 | | 28 | | 15.42 |  |
| 994 | 303.6 | 139.9 | | 0 | | 213.5 | | 6.2 | | 895.5 | | 722.5 | | 28 | | 33.42 |  |
| 995 | 374.3 | 0 | | 0 | | 190.2 | | 6.7 | | 1013.2 | | 730.4 | | 28 | | 39.06 |  |
| 996 | 158.6 | 148.9 | | 116 | | 175.1 | | 15 | | 953.3 | | 719.7 | | 28 | | 27.68 |  |
| 997 | 152.6 | 238.7 | | 0 | | 200 | | 6.3 | | 1001.8 | | 683.9 | | 28 | | 26.86 |  |
| 998 | 310 | 142.8 | | 0 | | 167.9 | | 10 | | 914.3 | | 804 | | 28 | | 45.3 |  |
| 999 | 304.8 | 0 | | 99.6 | | 196 | | 9.8 | | 959.4 | | 705.2 | | 28 | | 30.12 |  |
| 1000 | 150.9 | 0 | | 183.9 | | 166.6 | | 11.6 | | 991.2 | | 772.2 | | 28 | | 15.57 |  |
| 1001 | 141.9 | 166.6 | | 129.7 | | 173.5 | | 10.9 | | 882.6 | | 785.3 | | 28 | | 44.61 |  |
| 1002 | 297.8 | 137.2 | | 106.9 | | 201.3 | | 6 | | 878.4 | | 655.3 | | 28 | | 53.52 |  |
| 1003 | 321.3 | 164.2 | | 0 | | 190.5 | | 4.6 | | 870 | | 774 | | 28 | | 57.22 |  |
| 1004 | 366 | 187 | | 0 | | 191.3 | | 6.6 | | 824.3 | | 756.9 | | 28 | | 65.91 |  |
| 1005 | 279.8 | 128.9 | | 100.4 | | 172.4 | | 9.5 | | 825.1 | | 804.9 | | 28 | | 52.83 |  |
| 1006 | 252.1 | 97.1 | | 75.6 | | 193.8 | | 8.3 | | 835.5 | | 821.4 | | 28 | | 33.4 |  |
| 1007 | 164.6 | 0 | | 150.4 | | 181.6 | | 11.7 | | 1023.3 | | 728.9 | | 28 | | 18.03 |  |
| 1008 | 155.6 | 243.5 | | 0 | | 180.3 | | 10.7 | | 1022 | | 697.7 | | 28 | | 37.36 |  |
| 1009 | 160.2 | 188 | | 146.4 | | 203.2 | | 11.3 | | 828.7 | | 709.7 | | 28 | | 35.31 |  |
| 1010 | 298.1 | 0 | | 107 | | 186.4 | | 6.1 | | 879 | | 815.2 | | 28 | | 42.64 |  |
| 1011 | 317.9 | 0 | | 126.5 | | 209.7 | | 5.7 | | 860.5 | | 736.6 | | 28 | | 40.06 |  |
| 1012 | 287.3 | 120.5 | | 93.9 | | 187.6 | | 9.2 | | 904.4 | | 695.9 | | 28 | | 43.8 |  |
| 1013 | 325.6 | 166.4 | | 0 | | 174 | | 8.9 | | 881.6 | | 790 | | 28 | | 61.24 |  |
| 1014 | 355.9 | 0 | | 141.6 | | 193.3 | | 11 | | 801.4 | | 778.4 | | 28 | | 40.87 |  |
| 1015 | 132 | 206.5 | | 160.9 | | 178.9 | | 5.5 | | 866.9 | | 735.6 | | 28 | | 33.31 |  |
| 1016 | 322.5 | 148.6 | | 0 | | 185.8 | | 8.5 | | 951 | | 709.5 | | 28 | | 52.43 |  |
| 1017 | 164.2 | 0 | | 200.1 | | 181.2 | | 12.6 | | 849.3 | | 846 | | 28 | | 15.09 |  |
| 1018 | 313.8 | 0 | | 112.6 | | 169.9 | | 10.1 | | 925.3 | | 782.9 | | 28 | | 38.46 |  |
| 1019 | 321.4 | 0 | | 127.9 | | 182.5 | | 11.5 | | 870.1 | | 779.7 | | 28 | | 37.27 |  |
| 1020 | 139.7 | 163.9 | | 127.7 | | 236.7 | | 5.8 | | 868.6 | | 655.6 | | 28 | | 35.23 |  |
| 1021 | 288.4 | 121 | | 0 | | 177.4 | | 7 | | 907.9 | | 829.5 | | 28 | | 42.14 |  |
| 1022 | 298.2 | 0 | | 107 | | 209.7 | | 11.1 | | 879.6 | | 744.2 | | 28 | | 31.88 |  |
| 1023 | 264.5 | 111 | | 86.5 | | 195.5 | | 5.9 | | 832.6 | | 790.4 | | 28 | | 41.54 |  |
| 1024 | 159.8 | 250 | | 0 | | 168.4 | | 12.2 | | 1049.3 | | 688.2 | | 28 | | 39.46 |  |
| 1025 | 166 | 259.7 | | 0 | | 183.2 | | 12.7 | | 858.8 | | 826.8 | | 28 | | 37.92 |  |
| 1026 | 276.4 | 116 | | 90.3 | | 179.6 | | 8.9 | | 870.1 | | 768.3 | | 28 | | 44.28 |  |
| 1027 | 322.2 | 0 | | 115.6 | | 196 | | 10.4 | | 817.9 | | 813.4 | | 28 | | 31.18 |  |
| 1028 | 148.5 | 139.4 | | 108.6 | | 192.7 | | 6.1 | | 892.4 | | 780 | | 28 | | 23.7 |  |
| 1029 | 159.1 | 186.7 | | 0 | | 175.6 | | 11.3 | | 989.6 | | 788.9 | | 28 | | 32.77 |  |
| 1030 | 260.9 | 100.5 | | 78.3 | | 200.6 | | 8.6 | | 864.5 | | 761.5 | | 28 | | 32.4 |  |

**Dataset 2**

| number | water | cement | fine_aggregate | coarse_aggregate | superplastic | strength |
| --- | --- | --- | --- | --- | --- | --- |
| 1 | 160 | 533 | 805 | 845 | 1 | 73.6 |
| 2 | 160 | 533 | 805 | 845 | 1.5 | 73.6 |
| 3 | 160 | 533 | 805 | 845 | 2 | 73.6 |
| 4 | 160 | 480 | 786 | 845 | 1 | 73.1 |
| 5 | 160 | 480 | 786 | 845 | 1.5 | 73.1 |
| 6 | 160 | 480 | 786 | 845 | 2 | 73.1 |
| 7 | 160 | 427 | 767 | 845 | 1 | 72.7 |
| 8 | 160 | 427 | 767 | 845 | 1.5 | 72.7 |
| 9 | 160 | 427 | 767 | 845 | 2 | 72.7 |
| 10 | 160 | 533 | 753 | 898 | 1 | 69.4 |
| 11 | 160 | 533 | 753 | 898 | 1.5 | 69.4 |
| 12 | 160 | 533 | 753 | 898 | 2 | 69.4 |
| 13 | 160 | 480 | 734 | 898 | 1 | 70.5 |
| 14 | 160 | 480 | 734 | 898 | 1.5 | 70.5 |
| 15 | 160 | 480 | 734 | 898 | 2 | 70.5 |
| 16 | 160 | 427 | 715 | 898 | 1 | 68.1 |
| 17 | 160 | 427 | 715 | 898 | 1.5 | 68.1 |
| 18 | 160 | 427 | 715 | 898 | 2 | 68.1 |
| 19 | 160 | 533 | 701 | 950 | 1 | 67.8 |
| 20 | 160 | 533 | 701 | 950 | 1.5 | 67.8 |
| 21 | 160 | 533 | 701 | 950 | 2 | 67.8 |
| 22 | 160 | 480 | 682 | 950 | 1 | 67 |
| 23 | 160 | 480 | 682 | 950 | 1.5 | 67 |
| 24 | 160 | 480 | 682 | 950 | 2 | 67 |
| 25 | 160 | 427 | 663 | 950 | 1 | 64.1 |
| 26 | 160 | 427 | 663 | 950 | 1.5 | 64.1 |
| 27 | 160 | 427 | 663 | 950 | 2 | 64.1 |
| 28 | 170 | 567 | 751 | 845 | 1 | 64.6 |
| 29 | 170 | 567 | 751 | 845 | 1.5 | 64.6 |
| 30 | 170 | 567 | 751 | 845 | 2 | 64.6 |
| 31 | 170 | 510 | 731 | 845 | 1 | 64.4 |
| 32 | 170 | 510 | 731 | 845 | 1.5 | 64.4 |
| 33 | 170 | 510 | 731 | 845 | 2 | 64.4 |
| 34 | 170 | 453 | 711 | 845 | 1 | 64.7 |
| 35 | 170 | 453 | 711 | 845 | 1.5 | 64.7 |
| 36 | 170 | 453 | 711 | 845 | 2 | 64.7 |
| 37 | 170 | 567 | 700 | 898 | 1 | 63.9 |
| 38 | 170 | 567 | 700 | 898 | 1.5 | 63.9 |
| 39 | 170 | 567 | 700 | 898 | 2 | 63.9 |
| 40 | 170 | 510 | 679 | 898 | 1 | 63.4 |
| 41 | 170 | 510 | 679 | 898 | 1.5 | 63.4 |
| 42 | 170 | 510 | 679 | 898 | 2 | 63.4 |
| 43 | 170 | 453 | 659 | 898 | 1 | 62 |
| 44 | 170 | 453 | 659 | 898 | 1.5 | 62 |
| 45 | 170 | 453 | 659 | 898 | 2 | 62 |
| 46 | 170 | 567 | 648 | 950 | 1 | 62.4 |
| 47 | 170 | 567 | 648 | 950 | 1.5 | 62.4 |
| 48 | 170 | 567 | 648 | 950 | 2 | 62.4 |
| 49 | 170 | 510 | 628 | 950 | 1 | 61.7 |
| 50 | 170 | 510 | 628 | 950 | 1.5 | 61.7 |
| 51 | 170 | 510 | 628 | 950 | 2 | 61.7 |
| 52 | 170 | 453 | 608 | 950 | 1 | 61.9 |
| 53 | 170 | 453 | 608 | 950 | 1.5 | 61.9 |
| 54 | 170 | 453 | 608 | 950 | 2 | 61.9 |
| 55 | 180 | 600 | 698 | 845 | 0.75 | 59.5 |
| 56 | 180 | 600 | 698 | 845 | 1.25 | 59.5 |
| 57 | 180 | 600 | 698 | 845 | 1.75 | 59.5 |
| 58 | 180 | 540 | 677 | 845 | 0.75 | 61.1 |
| 59 | 180 | 540 | 677 | 845 | 1.25 | 61.1 |
| 60 | 180 | 540 | 677 | 845 | 1.75 | 61.1 |
| 61 | 180 | 480 | 655 | 845 | 0.75 | 60.8 |
| 62 | 180 | 480 | 655 | 845 | 1.25 | 60.8 |
| 63 | 180 | 480 | 655 | 845 | 1.75 | 60.8 |
| 64 | 180 | 600 | 646 | 898 | 0.75 | 60.5 |
| 65 | 180 | 600 | 646 | 898 | 1.25 | 60.5 |
| 66 | 180 | 600 | 646 | 898 | 1.75 | 60.5 |
| 67 | 180 | 540 | 625 | 898 | 0.75 | 59.9 |
| 68 | 180 | 540 | 625 | 898 | 1.25 | 59.9 |
| 69 | 180 | 540 | 625 | 898 | 1.75 | 59.9 |
| 70 | 180 | 480 | 604 | 898 | 0.75 | 57 |
| 71 | 180 | 480 | 604 | 898 | 1.25 | 57 |
| 72 | 180 | 480 | 604 | 898 | 1.75 | 57 |
| 73 | 180 | 600 | 594 | 950 | 0.75 | 59.7 |
| 74 | 180 | 600 | 594 | 950 | 1.25 | 59.7 |
| 75 | 180 | 600 | 594 | 950 | 1.75 | 59.7 |
| 76 | 180 | 540 | 573 | 950 | 0.75 | 60 |
| 77 | 180 | 540 | 573 | 950 | 1.25 | 60 |
| 78 | 180 | 540 | 573 | 950 | 1.75 | 60 |
| 79 | 180 | 480 | 552 | 950 | 0.75 | 59.6 |
| 80 | 180 | 480 | 552 | 950 | 1.25 | 59.6 |
| 81 | 180 | 480 | 552 | 950 | 1.75 | 59.6 |
| 82 | 160 | 457 | 867 | 845 | 0.75 | 62 |
| 83 | 160 | 457 | 867 | 845 | 1.25 | 62 |
| 84 | 160 | 457 | 867 | 845 | 1.75 | 62 |
| 85 | 160 | 411 | 851 | 845 | 0.75 | 62 |
| 86 | 160 | 411 | 851 | 845 | 1.25 | 62 |
| 87 | 160 | 411 | 851 | 845 | 1.75 | 62 |
| 88 | 160 | 366 | 835 | 845 | 0.75 | 60.6 |
| 89 | 160 | 366 | 835 | 845 | 1.25 | 60.6 |
| 90 | 160 | 366 | 835 | 845 | 1.75 | 60.6 |
| 91 | 160 | 457 | 816 | 898 | 0.75 | 62.1 |
| 92 | 160 | 457 | 816 | 898 | 1.25 | 62.1 |
| 93 | 160 | 457 | 816 | 898 | 1.75 | 62.1 |
| 94 | 160 | 411 | 799 | 898 | 0.75 | 61.5 |
| 95 | 160 | 411 | 799 | 898 | 1.25 | 61.5 |
| 96 | 160 | 411 | 799 | 898 | 1.75 | 61.5 |
| 97 | 160 | 366 | 783 | 898 | 0.75 | 57.8 |
| 98 | 160 | 366 | 783 | 898 | 1.25 | 57.8 |
| 99 | 160 | 366 | 783 | 898 | 1.75 | 57.8 |
| 100 | 160 | 457 | 764 | 950 | 0.75 | 61.5 |
| 101 | 160 | 457 | 764 | 950 | 1.25 | 61.5 |
| 102 | 160 | 457 | 764 | 950 | 1.75 | 61.5 |
| 103 | 160 | 411 | 747 | 950 | 0.75 | 60.8 |
| 104 | 160 | 411 | 747 | 950 | 1.25 | 60.8 |
| 105 | 160 | 411 | 747 | 950 | 1.75 | 60.8 |
| 106 | 160 | 366 | 731 | 950 | 0.75 | 57.6 |
| 107 | 160 | 366 | 731 | 950 | 1.25 | 57.6 |
| 108 | 160 | 366 | 731 | 950 | 1.75 | 57.6 |
| 109 | 170 | 486 | 818 | 845 | 0.5 | 58.8 |
| 110 | 170 | 486 | 818 | 845 | 1 | 58.8 |
| 111 | 170 | 486 | 818 | 845 | 1.5 | 58.8 |
| 112 | 170 | 437 | 801 | 845 | 0.5 | 56.8 |
| 113 | 170 | 437 | 801 | 845 | 1 | 56.8 |
| 114 | 170 | 437 | 801 | 845 | 1.5 | 56.8 |
| 115 | 170 | 389 | 783 | 845 | 0.5 | 55.3 |
| 116 | 170 | 389 | 783 | 845 | 1 | 55.3 |
| 117 | 170 | 389 | 783 | 845 | 1.5 | 55.3 |
| 118 | 170 | 486 | 766 | 898 | 0.5 | 57.8 |
| 119 | 170 | 486 | 766 | 898 | 1 | 57.8 |
| 120 | 170 | 486 | 766 | 898 | 1.5 | 57.8 |
| 121 | 170 | 437 | 749 | 898 | 0.5 | 56.6 |
| 122 | 170 | 437 | 749 | 898 | 1 | 56.6 |
| 123 | 170 | 437 | 749 | 898 | 1.5 | 56.6 |
| 124 | 170 | 389 | 732 | 898 | 0.5 | 56.9 |
| 125 | 170 | 389 | 732 | 898 | 1 | 56.9 |
| 126 | 170 | 389 | 732 | 898 | 1.5 | 56.9 |
| 127 | 170 | 486 | 714 | 950 | 0.5 | 56.1 |
| 128 | 170 | 486 | 714 | 950 | 1 | 56.1 |
| 129 | 170 | 486 | 714 | 950 | 1.5 | 56.1 |
| 130 | 170 | 437 | 697 | 950 | 0.5 | 55.9 |
| 131 | 170 | 437 | 697 | 950 | 1 | 55.9 |
| 132 | 170 | 437 | 697 | 950 | 1.5 | 55.9 |
| 133 | 170 | 389 | 680 | 950 | 0.5 | 54.3 |
| 134 | 170 | 389 | 680 | 950 | 1 | 54.3 |
| 135 | 170 | 389 | 680 | 950 | 1.5 | 54.3 |
| 136 | 180 | 514 | 769 | 845 | 0.25 | 54.2 |
| 137 | 180 | 514 | 769 | 845 | 0.75 | 54.2 |
| 138 | 180 | 514 | 769 | 845 | 1.25 | 54.2 |
| 139 | 180 | 463 | 750 | 845 | 0.25 | 52.7 |
| 140 | 180 | 463 | 750 | 845 | 0.75 | 52.7 |
| 141 | 180 | 463 | 750 | 845 | 1.25 | 52.7 |
| 142 | 180 | 411 | 732 | 845 | 0.25 | 51 |
| 143 | 180 | 411 | 732 | 845 | 0.75 | 51 |
| 144 | 180 | 411 | 732 | 845 | 1.25 | 51 |
| 145 | 180 | 514 | 717 | 898 | 0.25 | 54.6 |
| 146 | 180 | 514 | 717 | 898 | 0.75 | 54.6 |
| 147 | 180 | 514 | 717 | 898 | 1.25 | 54.6 |
| 148 | 180 | 463 | 689 | 898 | 0.25 | 50.3 |
| 149 | 180 | 463 | 689 | 898 | 0.75 | 50.3 |
| 150 | 180 | 463 | 689 | 898 | 1.25 | 50.3 |
| 151 | 180 | 411 | 680 | 898 | 0.25 | 47.3 |
| 152 | 180 | 411 | 680 | 898 | 0.75 | 47.3 |
| 153 | 180 | 411 | 680 | 898 | 1.25 | 47.3 |
| 154 | 180 | 514 | 665 | 950 | 0.25 | 52.1 |
| 155 | 180 | 514 | 665 | 950 | 0.75 | 52.1 |
| 156 | 180 | 514 | 665 | 950 | 1.25 | 52.1 |
| 157 | 180 | 463 | 647 | 950 | 0.5 | 45.5 |
| 158 | 180 | 463 | 647 | 950 | 1 | 45.5 |
| 159 | 180 | 463 | 647 | 950 | 1.5 | 45.5 |
| 160 | 180 | 411 | 628 | 950 | 0.5 | 45.7 |
| 161 | 180 | 411 | 628 | 950 | 1 | 45.7 |
| 162 | 180 | 411 | 628 | 950 | 1.5 | 45.7 |
| 163 | 160 | 400 | 914 | 845 | 0.5 | 49.6 |
| 164 | 160 | 400 | 914 | 845 | 1 | 49.6 |
| 165 | 160 | 400 | 914 | 845 | 1.5 | 49.6 |
| 166 | 160 | 360 | 900 | 845 | 0.5 | 48 |
| 167 | 160 | 360 | 900 | 845 | 1 | 48 |
| 168 | 160 | 360 | 900 | 845 | 1.5 | 48 |
| 169 | 160 | 320 | 886 | 845 | 0.5 | 47.7 |
| 170 | 160 | 320 | 886 | 845 | 1 | 47.7 |
| 171 | 160 | 320 | 886 | 845 | 1.5 | 47.7 |
| 172 | 160 | 400 | 863 | 989 | 0.5 | 49.1 |
| 173 | 160 | 400 | 863 | 989 | 1 | 49.1 |
| 174 | 160 | 400 | 863 | 989 | 1.5 | 49.1 |
| 175 | 160 | 360 | 848 | 898 | 0.5 | 48 |
| 176 | 160 | 360 | 848 | 898 | 1 | 48 |
| 177 | 160 | 360 | 848 | 898 | 1.5 | 48 |
| 178 | 160 | 320 | 834 | 898 | 0.5 | 48.5 |
| 179 | 160 | 320 | 834 | 898 | 1 | 48.5 |
| 180 | 160 | 320 | 834 | 898 | 1.5 | 48.5 |
| 181 | 160 | 400 | 811 | 950 | 0.5 | 49.4 |
| 182 | 160 | 400 | 811 | 950 | 1 | 49.4 |
| 183 | 160 | 400 | 811 | 950 | 1.5 | 49.4 |
| 184 | 160 | 360 | 797 | 950 | 0.5 | 48.7 |
| 185 | 160 | 360 | 797 | 950 | 1 | 48.7 |
| 186 | 160 | 360 | 797 | 950 | 1.5 | 48.7 |
| 187 | 160 | 320 | 782 | 950 | 0.5 | 46.1 |
| 188 | 160 | 320 | 782 | 950 | 1 | 46.1 |
| 189 | 160 | 320 | 782 | 950 | 1.5 | 46.1 |
| 190 | 170 | 425 | 868 | 845 | 0 | 47.7 |
| 191 | 170 | 425 | 868 | 845 | 0.5 | 47.7 |
| 192 | 170 | 425 | 868 | 845 | 1 | 47.7 |
| 193 | 170 | 425 | 853 | 845 | 0 | 47.1 |
| 194 | 170 | 425 | 853 | 845 | 0.5 | 47.1 |
| 195 | 170 | 425 | 853 | 845 | 1 | 47.1 |
| 196 | 170 | 340 | 838 | 845 | 0 | 45 |
| 197 | 170 | 340 | 838 | 845 | 0.5 | 45 |
| 198 | 170 | 340 | 838 | 845 | 1 | 45 |
| 199 | 170 | 425 | 816 | 898 | 0 | 46 |
| 200 | 170 | 425 | 816 | 898 | 0.5 | 46 |
| 201 | 170 | 425 | 816 | 898 | 1 | 46 |
| 202 | 170 | 383 | 801 | 898 | 0 | 45.7 |
| 203 | 170 | 383 | 801 | 898 | 0.5 | 45.7 |
| 204 | 170 | 383 | 801 | 898 | 1 | 45.7 |
| 205 | 170 | 340 | 786 | 898 | 0 | 45.1 |
| 206 | 170 | 340 | 786 | 898 | 0.5 | 45.1 |
| 207 | 170 | 340 | 786 | 898 | 1 | 45.1 |
| 208 | 170 | 425 | 764 | 950 | 0 | 46 |
| 209 | 170 | 425 | 764 | 950 | 0.5 | 46 |
| 210 | 170 | 425 | 764 | 950 | 1 | 46 |
| 211 | 170 | 383 | 749 | 950 | 0 | 45 |
| 212 | 170 | 383 | 749 | 950 | 0.5 | 45 |
| 213 | 170 | 383 | 749 | 950 | 1 | 45 |
| 214 | 170 | 340 | 734 | 950 | 0 | 43.3 |
| 215 | 170 | 340 | 734 | 950 | 0.5 | 43.3 |
| 216 | 170 | 340 | 734 | 950 | 1 | 43.3 |
| 217 | 180 | 450 | 821 | 845 | 0 | 44.5 |
| 218 | 180 | 450 | 821 | 845 | 0.5 | 44.5 |
| 219 | 180 | 450 | 821 | 845 | 1 | 44.5 |
| 220 | 180 | 405 | 805 | 845 | 0 | 43.6 |
| 221 | 180 | 405 | 805 | 845 | 0.5 | 43.6 |
| 222 | 180 | 405 | 805 | 845 | 1 | 43.6 |
| 223 | 180 | 360 | 789 | 845 | 0 | 42 |
| 224 | 180 | 360 | 789 | 845 | 0.5 | 42 |
| 225 | 180 | 360 | 789 | 845 | 1 | 42 |
| 226 | 180 | 450 | 770 | 898 | 0 | 43.8 |
| 227 | 180 | 450 | 770 | 898 | 0.5 | 43.8 |
| 228 | 180 | 450 | 770 | 898 | 1 | 43.8 |
| 229 | 180 | 405 | 754 | 898 | 0 | 43 |
| 230 | 180 | 405 | 754 | 898 | 0.5 | 43 |
| 231 | 180 | 405 | 754 | 898 | 1 | 43 |
| 232 | 180 | 360 | 738 | 898 | 0 | 43.2 |
| 233 | 180 | 360 | 738 | 898 | 0.5 | 43.2 |
| 234 | 180 | 360 | 738 | 898 | 1 | 43.2 |
| 235 | 180 | 450 | 718 | 950 | 0 | 43.5 |
| 236 | 180 | 450 | 718 | 950 | 0.5 | 43.5 |
| 237 | 180 | 450 | 718 | 950 | 1 | 43.5 |
| 238 | 180 | 405 | 702 | 950 | 0 | 41.5 |
| 239 | 180 | 405 | 702 | 950 | 0.5 | 41.5 |
| 240 | 180 | 405 | 702 | 950 | 1 | 41.5 |
| 241 | 180 | 360 | 686 | 950 | 0 | 42.4 |
| 242 | 180 | 360 | 686 | 950 | 0.5 | 42.4 |
| 243 | 180 | 360 | 686 | 950 | 1 | 42.4 |
| 244 | 160 | 356 | 951 | 845 | 0 | 46 |
| 245 | 160 | 356 | 951 | 845 | 0.5 | 46 |
| 246 | 160 | 356 | 951 | 845 | 1 | 46 |
| 247 | 160 | 320 | 938 | 845 | 0 | 45 |
| 248 | 160 | 320 | 938 | 845 | 0.5 | 45 |
| 249 | 160 | 320 | 938 | 845 | 1 | 45 |
| 250 | 160 | 284 | 926 | 845 | 0 | 43.7 |
| 251 | 160 | 284 | 926 | 845 | 0.5 | 43.7 |
| 252 | 160 | 284 | 926 | 845 | 1 | 43.7 |
| 253 | 160 | 356 | 899 | 898 | 0 | 44.5 |
| 254 | 160 | 356 | 899 | 898 | 0.5 | 44.5 |
| 255 | 160 | 356 | 899 | 898 | 1 | 44.5 |
| 256 | 160 | 320 | 886 | 898 | 0 | 42.6 |
| 257 | 160 | 320 | 886 | 898 | 0.5 | 42.6 |
| 258 | 160 | 320 | 886 | 898 | 1 | 42.6 |
| 259 | 160 | 284 | 874 | 898 | 0 | 43.8 |
| 260 | 160 | 284 | 874 | 898 | 0.5 | 43.8 |
| 261 | 160 | 284 | 874 | 898 | 1 | 43.8 |
| 262 | 160 | 356 | 847 | 950 | 0 | 43.6 |
| 263 | 160 | 356 | 847 | 950 | 0.5 | 43.6 |
| 264 | 160 | 356 | 847 | 950 | 1 | 43.6 |
| 265 | 160 | 320 | 835 | 950 | 0 | 42.6 |
| 266 | 160 | 320 | 835 | 950 | 0.5 | 42.6 |
| 267 | 160 | 320 | 835 | 950 | 1 | 42.6 |
| 268 | 160 | 284 | 822 | 950 | 0 | 42.9 |
| 269 | 160 | 284 | 822 | 950 | 0.5 | 42.9 |
| 270 | 160 | 284 | 822 | 950 | 1 | 42.9 |
| 271 | 170 | 378 | 907 | 845 | 0 | 44.9 |
| 272 | 170 | 378 | 907 | 845 | 0.5 | 44.9 |
| 273 | 170 | 378 | 907 | 845 | 1 | 44.9 |
| 274 | 170 | 340 | 893 | 845 | 0 | 41.1 |
| 275 | 170 | 340 | 893 | 845 | 0.5 | 41.1 |
| 276 | 170 | 340 | 893 | 845 | 1 | 41.1 |
| 277 | 170 | 302 | 880 | 845 | 0 | 41.5 |
| 278 | 170 | 302 | 880 | 845 | 0.5 | 41.5 |
| 279 | 170 | 302 | 880 | 845 | 1 | 41.5 |
| 280 | 170 | 378 | 855 | 898 | 0 | 42.5 |
| 281 | 170 | 378 | 855 | 898 | 0.5 | 42.5 |
| 282 | 170 | 378 | 855 | 898 | 1 | 42.5 |
| 283 | 170 | 340 | 842 | 898 | 0 | 40.8 |
| 284 | 170 | 340 | 842 | 898 | 0.5 | 40.8 |
| 285 | 170 | 340 | 842 | 898 | 1 | 40.8 |
| 286 | 170 | 302 | 828 | 898 | 0 | 40.8 |
| 287 | 170 | 302 | 828 | 898 | 0.5 | 40.8 |
| 288 | 170 | 302 | 828 | 898 | 1 | 40.8 |
| 289 | 170 | 378 | 803 | 950 | 0 | 41.8 |
| 290 | 170 | 378 | 803 | 950 | 0.5 | 41.8 |
| 291 | 170 | 378 | 803 | 950 | 1 | 41.8 |
| 292 | 170 | 340 | 790 | 950 | 0 | 41.3 |
| 293 | 170 | 340 | 790 | 950 | 0.5 | 41.3 |
| 294 | 170 | 340 | 790 | 950 | 1 | 41.3 |
| 295 | 170 | 302 | 776 | 950 | 0 | 41 |
| 296 | 170 | 302 | 776 | 950 | 0.5 | 41 |
| 297 | 170 | 302 | 776 | 950 | 1 | 41 |
| 298 | 180 | 400 | 863 | 845 | 0 | 41.3 |
| 299 | 180 | 400 | 863 | 845 | 0.5 | 41.3 |
| 300 | 180 | 400 | 863 | 845 | 1 | 41.3 |
| 301 | 180 | 360 | 848 | 845 | 0 | 41.5 |
| 302 | 180 | 360 | 848 | 845 | 0.5 | 41.5 |
| 303 | 180 | 360 | 848 | 845 | 1 | 41.5 |
| 304 | 180 | 320 | 834 | 845 | 0 | 40.3 |
| 305 | 180 | 320 | 834 | 845 | 0.5 | 40.3 |
| 306 | 180 | 320 | 834 | 845 | 1 | 40.3 |
| 307 | 180 | 400 | 811 | 898 | 0 | 41.5 |
| 308 | 180 | 400 | 811 | 898 | 0.5 | 41.5 |
| 309 | 180 | 400 | 811 | 898 | 1 | 41.5 |
| 310 | 180 | 360 | 797 | 898 | 0 | 40 |
| 311 | 180 | 360 | 797 | 898 | 0.5 | 40 |
| 312 | 180 | 360 | 797 | 898 | 1 | 40 |
| 313 | 180 | 320 | 782 | 898 | 0 | 40 |
| 314 | 180 | 320 | 782 | 898 | 0.5 | 40 |
| 315 | 180 | 320 | 782 | 898 | 1 | 40 |
| 316 | 180 | 400 | 759 | 950 | 0 | 42.1 |
| 317 | 180 | 400 | 759 | 950 | 0.5 | 42.1 |
| 318 | 180 | 400 | 759 | 950 | 1 | 42.1 |
| 319 | 180 | 360 | 745 | 950 | 0 | 39.5 |
| 320 | 180 | 360 | 745 | 950 | 0.5 | 39.5 |
| 321 | 180 | 360 | 745 | 950 | 1 | 39.5 |
| 322 | 180 | 320 | 731 | 950 | 0 | 37.5 |
| 323 | 180 | 320 | 731 | 950 | 0.5 | 37.5 |
| 324 | 180 | 320 | 731 | 950 | 1 | 37.5 |
